# Biocatalyzed aza-michael addition *via* continuous flow technology: a game-changer for the facile synthesis of *N-*alkylated pyrazole derivatives

**DOI:** 10.1039/d5ra08312e

**Published:** 2026-01-14

**Authors:** Li-Hua Du, Bing-Lin Yan, Miao-Miao Xue, Lin Wang, Xi-Ping Luo

**Affiliations:** a College of Pharmaceutical Science, ZheJiang University of Technology Hangzhou 310014 Zhejiang China orgdlh@zjut.edu.cn +86 57188320903 +86 18969069399; b Zhejiang Provincial Key Laboratory of Chemical Utilization of Forestry Biomass, Zhejiang A&F University Hangzhou Zhejiang 311300 China

## Abstract

*N*-Alkylated pyrazole derivatives are widely used in the treatment of prostate cancer, depression, epilepsy and sickle cell disease due to their remarkable anti-tumor, anti-depressant and anti-bacterial activity. In this work, a convenient synthesis of *N-*alkylated pyrazole derivatives from pyrazoles and α,β-unsaturated compounds catalyzed by Lipozyme® TL IM/K_2_CO_3_ in a continuous-flow microreactor was studied. Through the study of reaction parameters such as enzyme type, the Lipozyme® TL IM/K_2_CO_3_ mixed catalyst ratio, reaction solvent, substrate molar ratio, temperature, residence time, *etc.*, the best reaction conditions for the enzymatic synthesis of *N-*alkylated pyrazole derivatives were obtained. The effects of electronic effects and steric hindrance of donors and acceptors were explored. The reaction was conducted in a shaker reactor and a continuous-flow microreactor respectively, and their space-time yields were compared. This research provides a novel technology for the facile synthesis of *N-*alkylated pyrazole derivatives and a versatile compound library to support subsequent research in pharmaceuticals and related domains.

## Abstract 2

An efficient continuous-flow biocatalytic synthesis of *N*-alkylated pyrazoles was established, with 25 derivatives prepared to verify applicability and supply pharmaceutical parameters.

## Graphical abstract

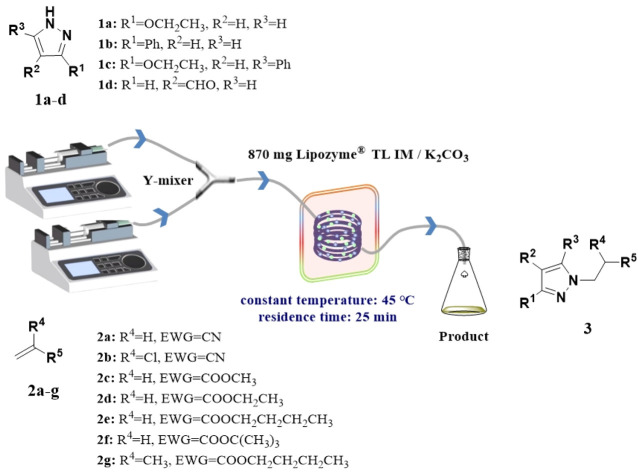

## Introduction

Heterocyclic scaffolds with tunable bioactivity lead global pharmaceutical/agrochemical innovation, and *N-*alkylated pyrazole derivatives stand out as highly impactful functional molecules.^1–3^ Their significance is corroborated by clinical and commercial evidence: over 40 pyrazole-containing pharmaceuticals (anti-inflammatory celecoxib, anticoagulant apixaban, type 2 diabetes therapy dorzagliatin) have U.S. Food and Drug Administration (FDA) approval, with the 2024 global *N-*alkylated pyrazole sector reaching $257.87 billion and projected to hit $420.58 billion by 2030. These data underscore the urgent demand for efficient, scalable synthesis of structurally diverse *N-*alkylated pyrazole derivatives.

**Fig. 1:**
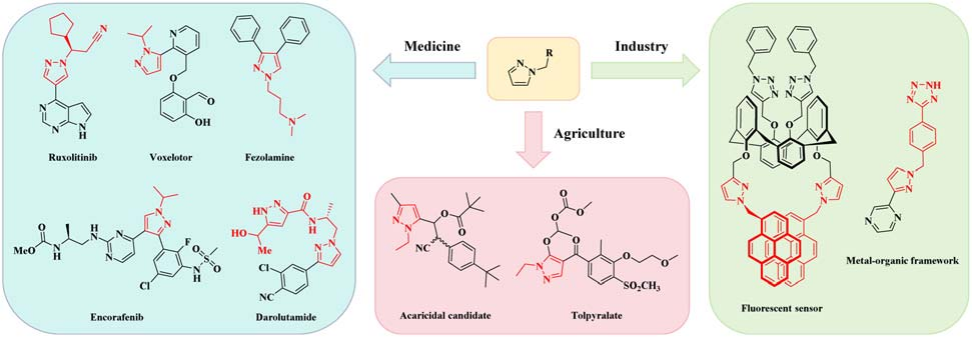
Application of *N-*alkylated pyrazole derivatives.

*N-*alkylated pyrazole derivatives are internationally recognized as a cornerstone class of bioactive scaffolds in modern pharmaceutical chemistry and translational research, exhibiting potent anti-cancer, anti-depressant, and antibacterial activities. A compelling body of literature has documented their therapeutic potential in addressing a spectrum of debilitating diseases, spanning prostate cancer, depression, epilepsy, and sickle cell disease.^1,4–11^ And in terms of agricultural planting, representative herbicides fluazolate and tolpyralate can prevent and control crop diseases, while pyrazole-3(5)-alkyl esters function as high-performance plant growth regulators that reduce chemical fertilizer usage and enhance crop yields, aligning with the global pursuit of sustainable agriculture.^12,13^ The unique chemical properties of *N-*alkylated pyrazole derivatives also driven their widespread adoption in high-value industrial sectors,^14–16^ integration into metal–organic framework (MOF) construction underpins advances in functional polymers, high-performance sensors, and next-generation batteries—bridging basic chemistry and industrial innovation (Fig. 1).

**Fig. 2:**
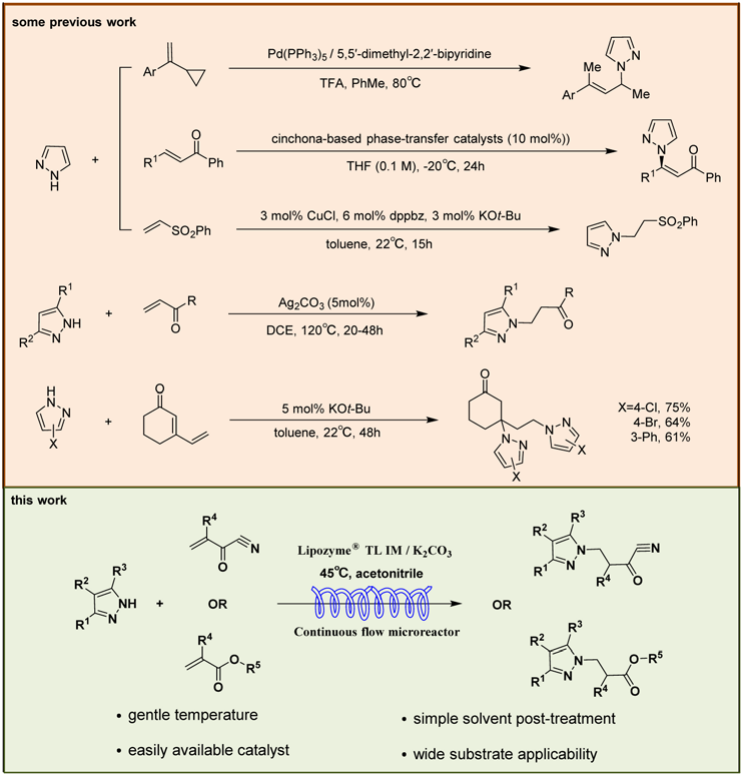
The relevant synthetic routes of *N-*alkylated pyrazole derivatives.

*N-*alkylated pyrazole derivatives serve as pivotal compounds in drug synthesis, agrochemical development, and industrial applications.^17–21^ Some synthetic routes enable the preparation of *N-*alkylated pyrazole derivatives, with routes involving condensation of hydrazines with 1,3-dicarbonyl compounds for C–N bond construction,^22^ relying on intermolecular [3 + 2] cycloaddition reactions^23^ and utilizing cross-coupling reactions between aryl electrophiles and substituted pyrazoles.^17^ The Michael addition reaction has emerged as the current mainstream strategy for synthesizing *N-*alkylated pyrazole derivatives (Fig. 2). Chemical synthetic protocols for *N-*alkylated pyrazole derivatives typically employ catalysts including CuCl,^24^ Ag_2_CO_3_,^25^ Pd(PPh_3_)_4_,^26^ and cinchona phase-transfer catalysts,^27^ with reaction temperatures ranging from −20 °C to 120 °C. 1,2-Dichloroethane (DCE),^25^ toluene,^24,28^ tetrahydrofuran (THF),^27^ and trifluoroacetic acid (TFA)^26^ were commonly used as the reaction solvent.

Biocatalysis serves as a key component of sustainable industrial chemistry and acts as a versatile, green synthesis strategy in organic synthesis,^29,30^ with applications in the preparation of vitamin B5,^31^ antibiotics,^32,33^ and MOF.^34^ Some of the derivatives (*e.g.*, 1,4-benzoxazolinone derivatives,^35^ purine nucleoside esters,^36^ and 2-methyl-3-*n*-butylaminoacyl-1*H*-4-quinone^33^) were synthesized in dimethyl sulfoxide (DMSO) or phosphate buffer, catalyzed by immobilized lipase from *T. lanuginosa*, *Candida antarctica* lipase B, laccases, and catalase@ZIF-L, and these reactions required 24 h or longer to achieve optimal yields in the Michael addition reaction. Some enzyme-catalyzed reactions utilize the peroxidase activity of haemoglobin,^37^ ethylenediamine-*N*,*N′*-disuccinic acid lyase (EDDS lyase),^18^ and proceed *via* the cyclization condensation of hydrazines with 1,3-diketones as well as the addition of arylhydrazines with fumarates to yield *N-*alkylated pyrazole derivatives. The synthesis of *N-*alkylated pyrazoles *via* enzyme-catalyzed Michael addition has few reports and biocatalysis faces challenges including limited enzyme source, longer reaction time to achieve ideal yield, the costs of enzyme that can be applied to the synthesis of *N-*alkylated pyrazole derivatives are relatively high. How to improve the reaction efficiency of biocatalytic Michael addition under the concept of green sustainability?

**Scheme 1:**
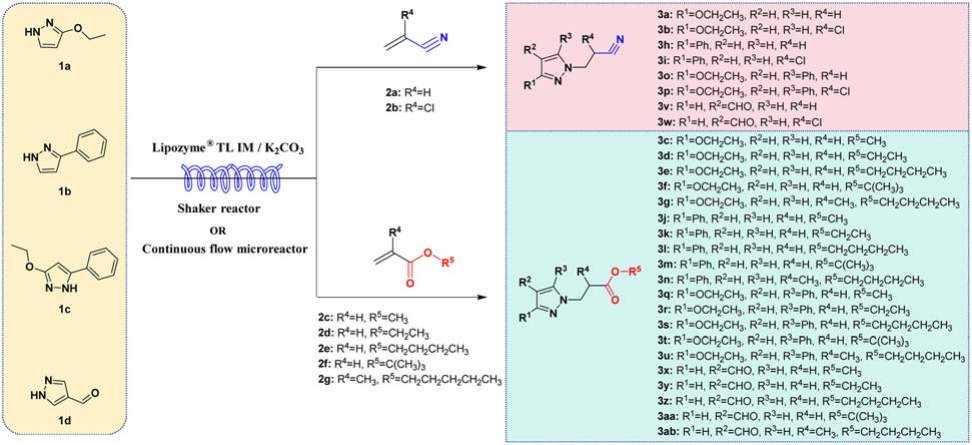
Enzymatic synthesis of *N-*alkylated pyrazole derivatives in continuous-flow microreactors.

Hailed as a “paradigm innovator” in modern manufacturing, continuous flow technology has evolved into a core driver underpinning the transformation of global pharmaceutical, fine chemical, and allied industries—owing to its unparalleled advantages in reaction control precision, process safety, and scalability.^38–41^ Integration of continuous-flow technology with biocatalysis significantly enhances reaction efficiency.^42–48^ In this paper, we aim to develop a biocatalyzed aza-Michael addition *via* continuous flow technology, a game-changer for the facile synthesis of *N-*alkylated pyrazole derivatives. To evaluate the feasibility of this approach, a systematic investigation of enzyme type, the Lipozyme® TL IM/K_2_CO_3_ mixed catalyst ratio, reaction solvent, substrate molar ratio, temperature, and residence time was conducted. The effects of substrates (pyrazoles and α,β-unsaturated compounds) with different electronic and steric effects on the reaction were explored. In order to verify the reaction efficiency of continuous flow biocatalytic technology and traditional biocatalytic technology, we compared the reaction in two different reactors in the same proportion, and obtained the best reaction parameters at the best yield of the reaction in the two reactors respectively. Finally, we conducted a study on the substrate scope, 4 pyrazoles (ethyl pyrazole-3-carboxylate, 3-phenylpyrazole, ethyl 5-phenylpyrazole-3-carboxylate, and 1*H*-pyrazole-4-carbaldehyde) were selected to react with 7 acrylonitrile/acrylate derivatives (acrylonitrile, 2-chloroacrylonitrile, methyl acrylate, ethyl acrylate, butyl acrylate, *tert*-butyl acrylate, and butyl methacrylate) *via* the aza-Michael addition reaction in continuous-flow microreactors, and 25 *N-*alkylated pyrazole derivatives were successfully obtained in parallel. This synthesis technique exhibits broad substrate applicability, providing technical references for the efficient synthesis of *N-*alkylated pyrazole drugs and the construction of related compound libraries (Scheme 1).

## Results and discussion

### Effect of enzyme type and the Lipozyme® TL IM/K_2_CO_3_ mixed catalyst ratio

Ethyl pyrazole-3-carboxylate and butyl acrylate were adopted as model substrates to investigate the synthesis of *N-*alkylated pyrazole derivatives in a continuous-flow microreactor, with reaction conditions involving enzyme-free systems and systems containing three distinct enzymes (Novozym® 435, Lipura Flex, Lipozyme® TL IM) respectively. The results showed that only Lipozyme® TL IM (entry 1–4, Table 1) catalyzed the reaction, while the conversion rate remained moderate (53%). To enhance conversion efficiency while preserving green synthesis characteristics, three weak basic catalysts (Et_3_N, Na_2_CO_3_, and K_2_CO_3_) were screened as promoters for Michael addition reactions in the enzyme-catalyzed mixed catalyst systems. Et_3_N, Na_2_CO_3_ and K_2_CO_3_ facilitated the reaction (entry 5–7, Table 1). Mixed systems of Lipozyme® TL IM with each base at a 9 : 1 mass ratio were compared, Et_3_N was excluded due to separation difficulties from the reaction solution and Na_2_CO_3_ generates by-products, leading to further investigation of the effect of K_2_CO_3_ dosage on reaction performance. Adjusting K_2_CO_3_ mass fraction in the Lipozyme® TL IM/K_2_CO_3_ mixed catalyst from 0% to 100%, a slight excess of K_2_CO_3_ caused decreased *N-*alkylated pyrazole derivatives yields and additional by-products formation. This phenomenon was attributed to substrate hydrolysis induced by excessive K_2_CO_3_, accompanied by transesterification. The optimal Lipozyme® TL IM/K_2_CO_3_ mixed catalyst ratio was achieved at a 20% K_2_CO_3_ mass fraction (Table 1).

**Table 1:** The effect of enzyme type and Lipozyme® TL IM/K_2_CO_3_ mixed catalyst ratio on the synthesis of *N-*alkylated pyrazole derivatives in continuous-flow microreactors

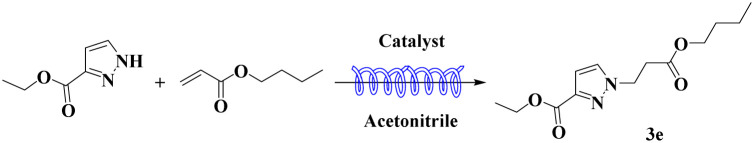		
Entry	Catalyst	Yield (%)
1	None	n.d.
2	Novozym® 435	n.d.
3	Lipura Flex	n.d.
4	Lipozyme® TL IM	53.68 ± 1.11
5	Et_3_N	50.28 ± 0.87
6	Na_2_CO_3_	43.79 ± 0.33
7	K_2_CO_3_	53.92 ± 0.62
8	Lipozyme® TL IM/Et_3_N = 9 : 1	62.15 ± 0.75
9	Lipozyme® TL IM/Na_2_CO_3_ = 9 : 1	55.31 ± 0.74
10	Lipozyme® TL IM/K_2_CO_3_ = 9 : 1	65.84 ± 0.43
11	Novozym® 435/K_2_CO_3_ = 9 : 1	12.63 ± 0.72
12	Lipozyme® TL IM/K_2_CO_3_ = 8 : 2	72.52 ± 1.04
13	Lipozyme® TL IM/K_2_CO_3_ = 7 : 3	69.16 ± 0.17
14	Lipozyme® TL IM/K_2_CO_3_ = 6 : 4	67.69 ± 0.55
15	Lipozyme® TL IM/K_2_CO_3_ = 5 : 5	66.70 ± 0.60
16	Lipozyme® TL IM/K_2_CO_3_ = 4 : 6	65.53 ± 0.82
17	Lipozyme® TL IM/K_2_CO_3_ = 3 : 7	62.81 ± 0.38
18	Lipozyme® TL IM/K_2_CO_3_ = 2 : 8	61.98 ± 0.39
19	Lipozyme® TL IM/K_2_CO_3_ = 1 : 9	58.53 ± 0.66

### Effect of reaction solvent

The selection of organic solvents is typically crucial for enzymatic reactions, and lipases are known to exhibit high activity in hydrophobic solvents. log *P* refers to the logarithm of the concentration ratio of a substance in an octanol–water mixture. The dielectric constant represents the ability to separate opposite charges in a solution and reflects the polarity of solvent molecules. The initial reaction rate of lipases increases in hydrophobic solvents owing to their low log *P* values and high polarity. It is necessary to select a solvent that can effectively dissolve substrates while maintaining sufficient enzyme activity. DMSO and acetonitrile, which possess low log *P* values and high dielectric constants, achieve high conversion rates in this reaction system. DMSO is not suitable for large-scale industrial production due to difficulties in separation, and thus acetonitrile was chosen as the optimal solvent for this experiment (Table 2).

**Table 2:** The effect of reaction solvent on the enzymatic synthesis of *N-*alkylated pyrazole derivatives in continuous-flow microreactors

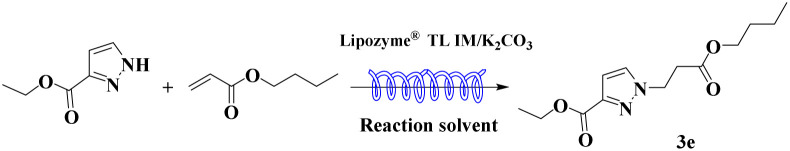				
Entry	Reaction solvent	log *P*	Dielectric constant	Yield (%)
1	Acetonitrile	−0.34	37.5	71.23 ± 0.83
2	DMSO	−1.35	46.7	72.77 ± 0.62
3	DMF	0.34	36.7	28.06 ± 1.18
4	THF	0.80	7.5	50.48 ± 0.57
5	Methanol	−0.77	32.7	<5
6	Ethanol	−0.19	24.5	42.61 ± 0.42
7	Isopropanol	−0.16	19.9	40.61 ± 0.89
8	*tert*-Amyl alcohol	0.89	5.8	21.87 ± 0.94

### Effect of substrate ratio

**Fig. 3:**
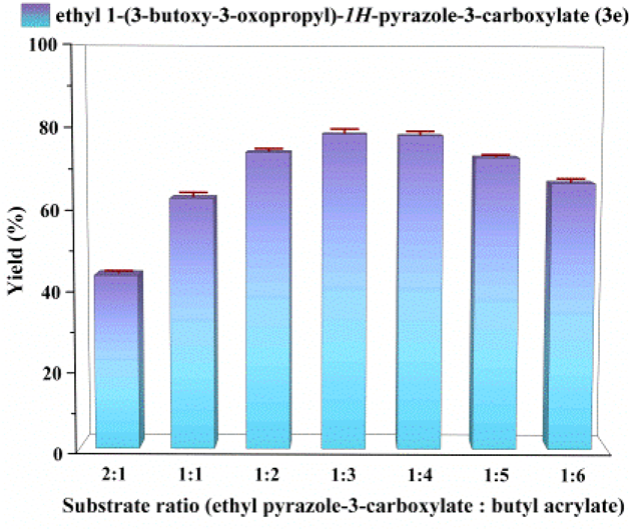
The effect of substrate molar ratio (ethyl pyrazole-3-carboxylate: butyl acrylate) on the enzymatic synthesis of *N-*alkylated pyrazole derivatives in continuous-flow microreactors.

Using ethyl pyrazole-3-carboxylate and butyl acrylate as the template reaction, the effect of substrate molar ratio on the reaction in a continuous flow microreactor was examined, with seven ratios (ethyl pyrazole-3-carboxylate: butyl acrylate) ranging from 2 : 1 to 1 : 6 investigated. Fig. 3 shows that increasing the proportion of butyl acrylate led to an increase in the yield of the target product. Optimal reaction performance was achieved at a ratio of 1 : 3. Further increase in the proportion of butyl acrylate beyond this point resulted in a decrease in conversion. This phenomenon may arise from elevated substrate concentrations increasing solution viscosity, which impairs substrate migration and diffusion rates within the carrier, thereby reducing reaction efficiency. A molar ratio of ethyl pyrazole-3-carboxylate to butyl acrylate of 1 : 3 was thus selected.

### Effect of reaction temperature

**Fig. 4:**
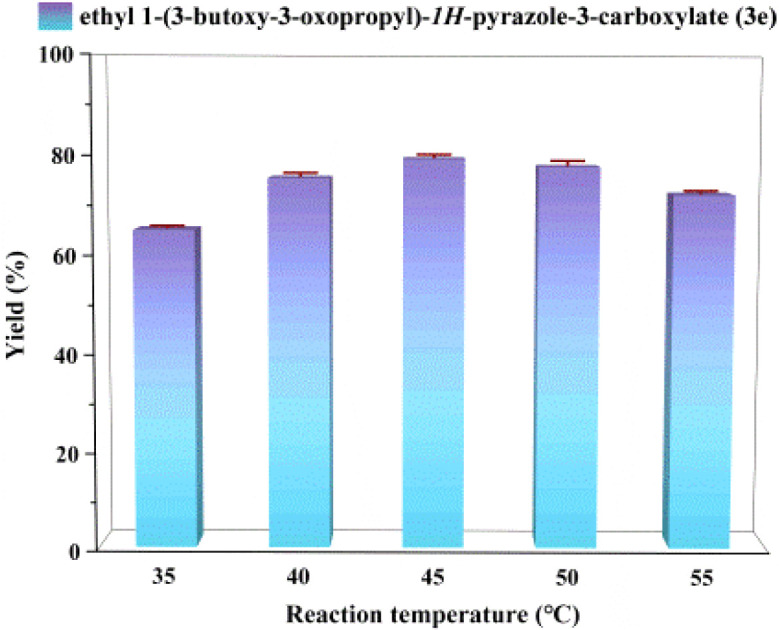
The effect of reaction temperature on the enzymatic synthesis of *N-*alkylated pyrazole derivatives in continuous-flow microreactors.

The effect of temperature was investigated using a Lipozyme® TL IM/K_2_CO_3_ mixture (20% K_2_CO_3_ by mass) as catalyst, acetonitrile as reaction medium, and a molar ratio of ethyl pyrazole-3-carboxylate to butyl acrylate of 1 : 3, with temperatures ranging from 35 °C to 55 °C. As shown in Fig. 4, reaction efficiency increased with temperature at lower ranges, while exceeding 45 °C led to a gradual decrease in efficiency. This trend may arise from enhanced substrate solubility and intermolecular collisions promoting enzymatic reaction at elevated temperatures. Temperatures exceeding 45 °C induce structural and property alterations in enzyme molecules, leading to enzyme denaturation and inactivation. A temperature of 45 °C emerged as optimal for subsequent studies.

### Effect of residence time

**Fig. 5:**
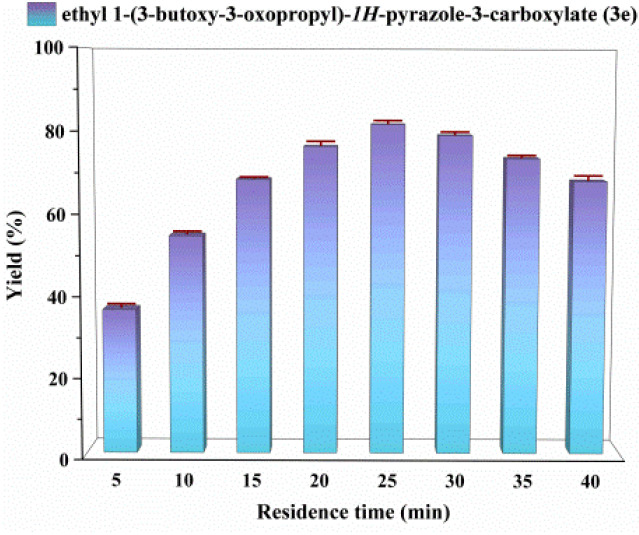
The effect of residence time on the enzymatic synthesis of *N-*alkylated pyrazole derivatives in continuous-flow microreactors.

In continuous-flow microreactors, maintaining a fixed length of the microporous tube while increasing the flow rate facilitated conversion and mass transfer, enabling sufficient contact between the enzyme active center and substrate molecules for enhanced conversion efficiency. As shown in Fig. 5, maximum yield was achieved at a residence time of 25 minutes (flow rate: 24.96 µL min^−1^). Prolonged reaction time beyond this point did not significantly increase yield but induced a decrease, attributed to further transesterification of the product with extended duration. A residence time of 25 minutes was thus selected as optimal for the study.

### The effect of enzyme reusability

**Fig. 6:**
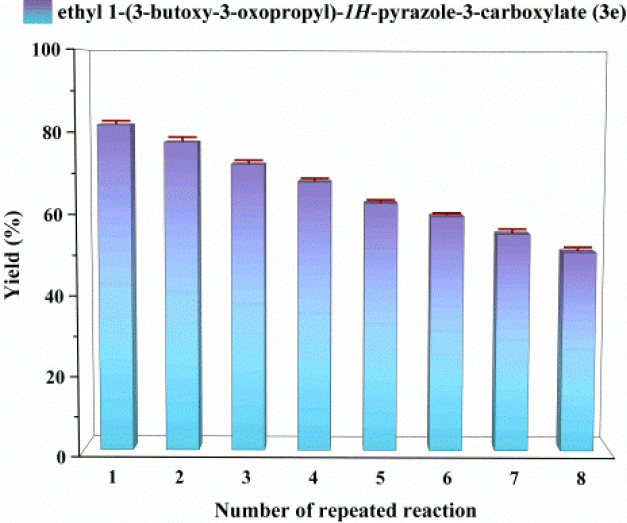
The effect of enzyme reusability on the enzymatic synthesis of *N-*alkylated pyrazole derivatives in continuous-flow microreactors.

Continuous-flow biocatalysis is known to be predominantly dependent on enzyme longevity, and the recovery and reuse of immobilized lipase are crucial for reducing operational costs. We evaluated the reusability of the enzyme in continuous-flow microreactors. The enzyme was recovered and reused for 8 consecutive cycles, with the catalytic yield remaining at 50% in the 8th run (Fig. 6). These results demonstrate that the enzyme exhibits satisfactory stability and reusability under continuous-flow conditions, indicating that the developed system has favorable economic benefits and practical applicability for the synthesis of *N-*alkylated pyrazole derivatives.

### Effect of substrate structure

**Fig. 7:**
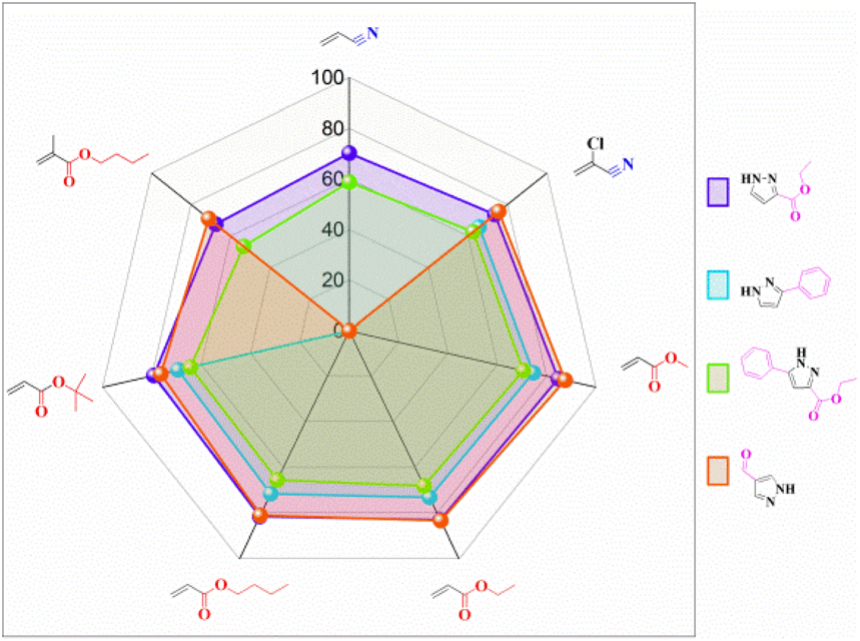
The effect of substrate structure on the enzymatic synthesis of *N-*alkylated pyrazole derivatives in continuous-flow microreactors.

The effect of pyrazoles with different substituents on the reaction was investigated. Pyrazoles bearing electron-withdrawing groups (EWGs) exhibited higher reactivity than those with electron-donating groups (EDGs). Under identical reaction conditions (with butyl acrylate as the acceptor), 1*H*-pyrazole-4-carbaldehyde achieved the highest yield (81.02%), while ethyl 5-phenylpyrazole-3-carboxylate showed the lowest yield (65.56%). This phenomenon was attributed to the presence of an aldehyde group in 1*H*-pyrazole-4-carbaldehyde, which enhanced its reactivity. In contrast, ethyl 5-phenylpyrazole-3-carboxylate contained two EDGs, and the phenyl group introduced steric hindrance. For acceptors in the Michael addition reaction, EWGs promoted the reaction, whereas acceptors with EDGs and longer aliphatic chains exhibited relatively lower reaction efficiency. Using ethyl pyrazole-3-carboxylate as the donor, the yield of the target product decreased with increasing aliphatic chain length in acrylates. Butyl methacrylate afforded the lowest yield (67.49%), likely due to steric hindrance imposed by the methyl group near the reaction site. Acrylonitrile consistently showed lower yields than acrylates, which may have resulted from the significant steric hindrance of the cyano group, exerting an influence on the reaction site (Fig. 7).

Differences in geometric configurations between batch reactors and continuous flow microreactors preclude direct comparison based solely on conversion rates. To investigate the influence of distinct reaction systems on enzymatic reactions, space-time yield (STY, units: g h^−1^ L^−1^) was employed to evaluate the productivity of each system, as its parameters enable fair comparison across different reaction setups. As shown in Table 4, the STY achieved in continuous flow microreactors was significantly higher than that in batch reactors. Batch reactors required 20 hours to reach the target yield, whereas continuous flow microreactors achieved not only the target yield but also higher productivity within just 25 minutes.1
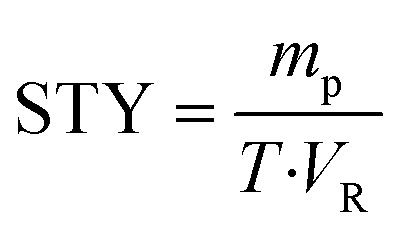
In the formula, *m*_p_ is the mass of the generated product (g), *T* is the residence time (h), and *V*_R_ is the reactor volume (L).

Following optimization of reaction conditions for the enzymatic Michael addition synthesis of *N-*alkylated pyrazole derivatives, the substrate scope of this methodology was further evaluated. Michael addition reactions between pyrazoles (ethyl pyrazole-3-carboxylate, 3-phenylpyrazole, ethyl 5-phenylpyrazole-3-carboxylate, and 1*H*-pyrazole-4-carbaldehyde) and α,β-unsaturated compounds (acrylonitrile, 2-chloroacrylonitrile, methyl acrylate, ethyl acrylate, butyl acrylate, *tert*-butyl acrylate, and butyl methacrylate) were investigated in both shake reactors and continuous-flow microreactors, with comparative analysis of their yields. Results confirm that synthesis of *N-*alkylated pyrazole derivatives in continuous-flow microreactors reduces reaction time to the minute scale while achieving higher conversion efficiency (Table 3).

**Table 3:** Comparison of *N-*alkylated pyrazole derivatives synthesized by continuous flow microreactors and shake reactors^a^

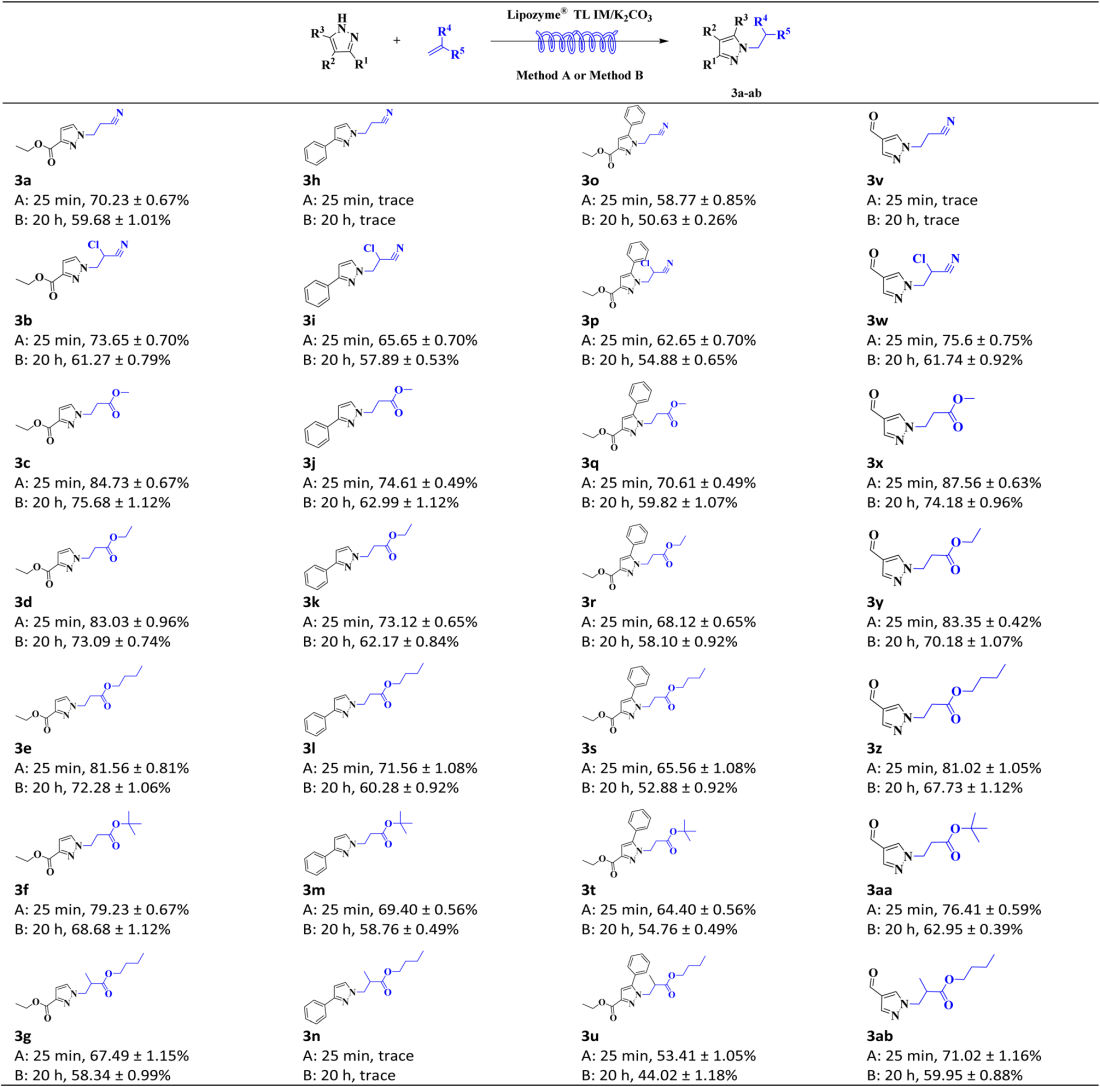

a General experimental conditions: Method A: continuous flow microreactors, feed 1, dissolve 5 mmol of pyrazoles in 10 mL of acetonitrile; feed 2, dissolve 15 mmol of α,β-unsaturated compounds in 10 mL of acetonitrile at a flow rate of 24.96 µL min^−1^ and a residence time of 25 min. Lipozyme® TL IM/K_2_CO_3_ 870 mg, 45 °C. Method B: shaker reactors, add 5 mmol of pyrazoles, 15 mmol of α,β-unsaturated compounds, and 20 mL of acetonitrile to a conical flask. Lipozyme® TL IM/K_2_CO_3_ 870 mg, 136 rpm, 50 °C, 20 h. Isolated yield. Yield: 100 × (actual amount obtained/calculated amount). The data is expressed as the mean ± standard deviation of three repeated experiment.

**Table 4:** Enzymatic synthesis of *N-*alkylated pyrazole derivatives in continuous-flow microreactors or shaker reactors^a^

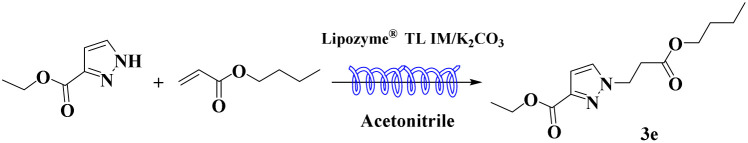			
Entry	Method	STY (g L^−1^ h^−1^)	Yield^b^ (%)
1	A	261.70	81.56 ± 0.81
2	B	0.98	72.28 ± 1.06

a General experimental conditions: Method A: continuous flow microreactors, feed 1, dissolve 5 mmol of ethyl pyrazole-3-carboxylate in 10 mL of acetonitrile; feed 2, dissolve 15 mmol of butyl acrylate in 10 mL of acetonitrile at a flow rate of 24.96 µL min^−1^, residence time of 25 min, Lipozyme® TL IM/K_2_CO_3_ 870 mg, 45 °C. Method B: shaker reactors, add 5 mmol of ethyl pyrazole-3-carboxylate, 15 mmol of butyl acrylate, and 20 mL of acetonitrile to a 50 mL conical flask, Lipozyme® TL IM/K_2_CO_3_ 870 mg, 136 rpm, 50 °C, 20 h.

b Isolated yield. Yield: 100 × (actual amount obtained/calculated amount). The data is expressed as the mean ± standard deviation of three repeated experiments.

## Experimental section

**Fig. 8:**
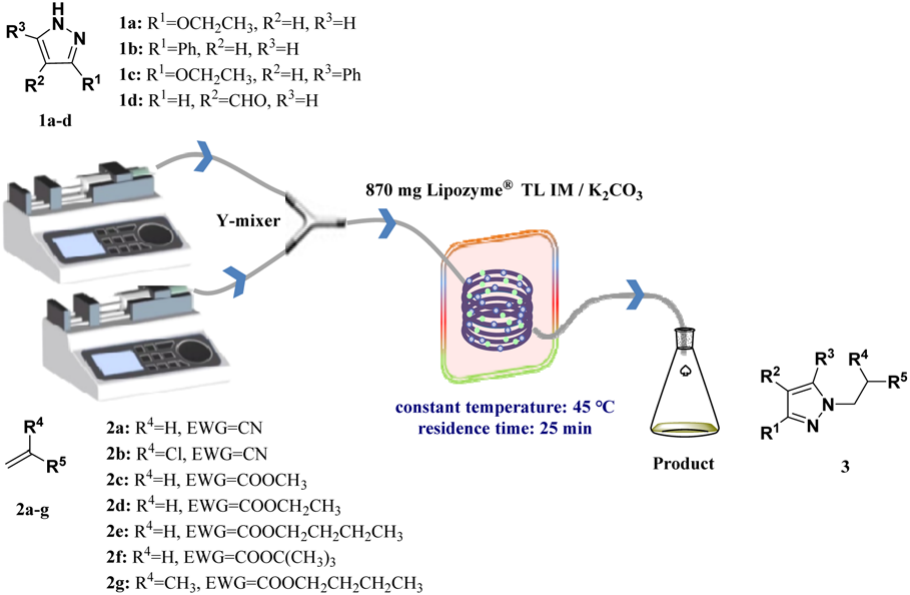
The equipment diagram for the synthesis of *N-*alkylated pyrazole derivatives in continuous-flow microreactors catalysed by Lipozyme® TL IM/K_2_CO_3_.

Fig. 8 shows the relevant equipment diagram for synthesizing *N-*alkylated pyrazole derivatives in a continuous flow microreactor. The experimental setup consists of a syringe pump (Harvard Instrumental Dr 2000), two substrate injectors, a Y-mixer, and a 100 cm × 2 mm PFA tubing filled with a uniform mixture of 870 mg of Lipozyme® TL IM/K_2_CO_3_, a constant temperature water bath, and a product collector. Dissolve 5 mmol of pyrazole derivative in 10 mL acetonitrile (feed 1) and 15 mmol of α,β-unsaturated compounds in 10 mL acetonitrile (feed 2). Mix the two at a flow rate of 24.96 mL min^−1^ in a Y-mixer with a residence time of 25 min. The PFA tubing filled with the Lipozyme® TL IM/K_2_CO_3_ mixed catalyst in a constant temperature water bath at 45 °C, and collect the effluent at the end using a conical flask. Separate the collected solution by silica gel chromatography to obtain the final product, and confirm its structure by ^1^H NMR and ^13^C NMR.

## Conclusions

*N-*alkylated pyrazole derivatives have significant biological activities, and their efficient construction is core to advancing drug synthesis and innovative development. In summary, a facile synthesis of *N-*alkylated pyrazole derivatives from pyrazoles and α,β-unsaturated compounds catalyzed by Lipozyme® TL IM/K_2_CO_3_ in a continuous-flow microreactor was developed. Investigation of reaction solvent, substrate molar ratio, temperature, and residence time enabled the determination of optimal reaction conditions for the enzymatic synthesis of *N-*alkylated pyrazole derivatives. Systematic studies were conducted on enzyme types and the Lipozyme® TL IM/K_2_CO_3_ mixed catalyst ratio, and 20% mass fraction of K_2_CO_3_ can significantly improve the reaction efficiency. A ratio of Lipozyme® TL IM to K_2_CO_3_ of 8 : 2 was determined to be applicable to this synthetic research, which also marks the first time that the Lipozyme® TL IM/K_2_CO_3_ mixed catalyst has been used for the synthesis of *N-*alkylated pyrazole derivatives. The prominent advantages of this method include relatively mild reaction conditions (45 °C), short reaction time (25 minutes), simple post-reaction processing (acetonitrile), readily available catalysts and excellent atom economy. The mechanistic studies were performed by examining pyrazoles and α,β-unsaturated compounds with different substituents, which enabled analysis of how electronic effects and steric hindrance influence the reaction. Reactions were carried out in shaker reactors and continuous-flow microreactors respectively, space-time yields were calculated and compared. The results showed that conducting the reaction under continuous-flow conditions could improve reaction efficiency and shorten reaction time. In parallel, substrate scope studies were performed, and 25 *N-*alkylated pyrazole derivatives were successfully synthesized *via* the aza-Michael addition reaction between 4 pyrazoles (ethyl pyrazole-3-carboxylate, 3-phenylpyrazole, ethyl 5-phenylpyrazole-3-carboxylate, and 1*H*-pyrazole-4-carbaldehyde) and 7 acrylonitrile/acrylate derivatives (acrylonitrile, 2-chloroacrylonitrile, methyl acrylate, ethyl acrylate, butyl acrylate, *tert*-butyl acrylate, and butyl methacrylate) in continuous-flow microreactors. This synthetic technology exhibits broad substrate applicability and provides valuable technical parameters for the efficient construction of active *N-*alkylated pyrazole-based drugs and their industrialization.

## Conflicts of interest

There are no conflicts to declare.

## Supplementary Material

RA-016-D5RA08312E-s001

## Data Availability

The authors confirm that the data supporting the findings of this study are available within its supplementary information (SI). Supplementary information is available. See DOI: https://doi.org/10.1039/d5ra08312e.
