# Supplementary material for: Biocatalyzed aza-michael addition via continuous flow technology: a game-changer for the facile synthesis of N-alkylated pyrazole derivatives
Source: RSC Adv. 2026 Jan 14;16(3):2850–7. doi: 10.1039/d5ra08312e (PMC12801137; doi:10.1039/d5ra08312e)
Supplement: RA-016-D5RA08312E-s001 [file RA-016-D5RA08312E-s001.pdf]

Supplementary Information (SI) for RSC Advances.
This journal is © The Royal Society of Chemistry 2026

Supporting Information for

Biocatalyzed Aza-Michael Addition via Continuous Flow Technology: A Game-Changer for the Facile Synthesis of *N*-Alkylated Pyrazole Derivatives

Bing-Lin Yan, [a] Li-Hua Du, *[a] Miao-Miao Xue, [a] Lin Wang [a] and Xi-Ping Luo *[b]

[a] College of Pharmaceutical Science, ZheJiang University of Technology, Hangzhou, 310014, China.

[b] Zhejiang Provincial Key Laboratory of Chemical Utilization of Forestry Biomass, Zhejiang A&F University, Hangzhou, 311300, China.

*Corresponding author. Fax: +86 18969069399. E-mail: orgdlh@zjut.edu.cn; orgdlh@gmail.com; luoxiping@zafu.edu.cn

Materials

All chemicals in this study were obtained from commercial sources and did not require further purification. Lipozyme® TL IM (immobilized *Thermomyces lanuginosus*) was purchased from Novo Nordisk (Copenhagen, Denmark). Ethyl pyrazole-3-carboxylate, 3-phenylpyrazole, 5-phenylpyrazole-3-carboxylate, and *1H*-pyrazole-4-carboxaldehyde were purchased from BidePharmatech Co., Ltd. (Shanghai, China). K_2CO_3, 2-chloroacrylonitrile, acrylonitrile, methyl acrylate, ethyl acrylate, *tert*-butyl acrylate, and butyl methacrylate were purchased from Titan (Shanghai, China). Butyl acrylate was purchased from Macklin (Shanghai, China). Harvard Instrument PHD 2000 syringe pump was purchased from Harvard University (Holliston, Massachusetts, USA). The flow reactor and Y-mixer were purchased from Beijing Haigui Medical Engineering Design Co., Ltd. (Beijing China).

Purification of the product

When the conversion of the *N*-alkylated pyrazole derivatives reaches a maximum (determined by TLC), the reaction is terminated by filtering the catalyst, and acetonitrile solvent is rotary evaporated under reduced pressure. The product is separated by silica gel chromatography (mobile phase petroleum ether / ethyl acetate, 20/1 to 1/1). Purification was monitored by TLC. The graded fractions containing the major product were combined, the solvent evaporated and the residue analyzed by ^{1}H NMR, ^{13}C NMR.

Experimental setup

Figure 1 illustrates a continuous flow microreactor device used for the synthesis of *N*-alkylated pyrazole compounds from pyrazoles and α,β- unsaturated compounds catalyzed by Lipozyme® TL IM/K_2CO_3. The experimental setup consists of a syringe pump, two substrate injectors, Y-shaped mixers (φ= 1.8 mm) and a product collector. Syringe pumps (Harvard apparatus PHD 2000) were used to introduce separate feed streams to the flow reactor with 100cm × 2 mm PFA tubing. Silica gel tubes were filled with Lipozyme® TL IM/K_2CO_3, and immersed in a constant temperature water bath to control the temperature. A total of 5 mmol of pyrazole in 10 mL of acetonitrile (feed 1), and 15 mmol of α,β- unsaturated compounds in 10 mL of acetonitrile (feed 2). Place feed 1 and 2 in separate 10 mL feeders and mix at a flow rate of 24.96 μL min^{-1} in a Y-type mixer at 45 °C. Connect the obtained stream (24.96 μL min^{-1}) to a sample bottle to collect the final mixture.

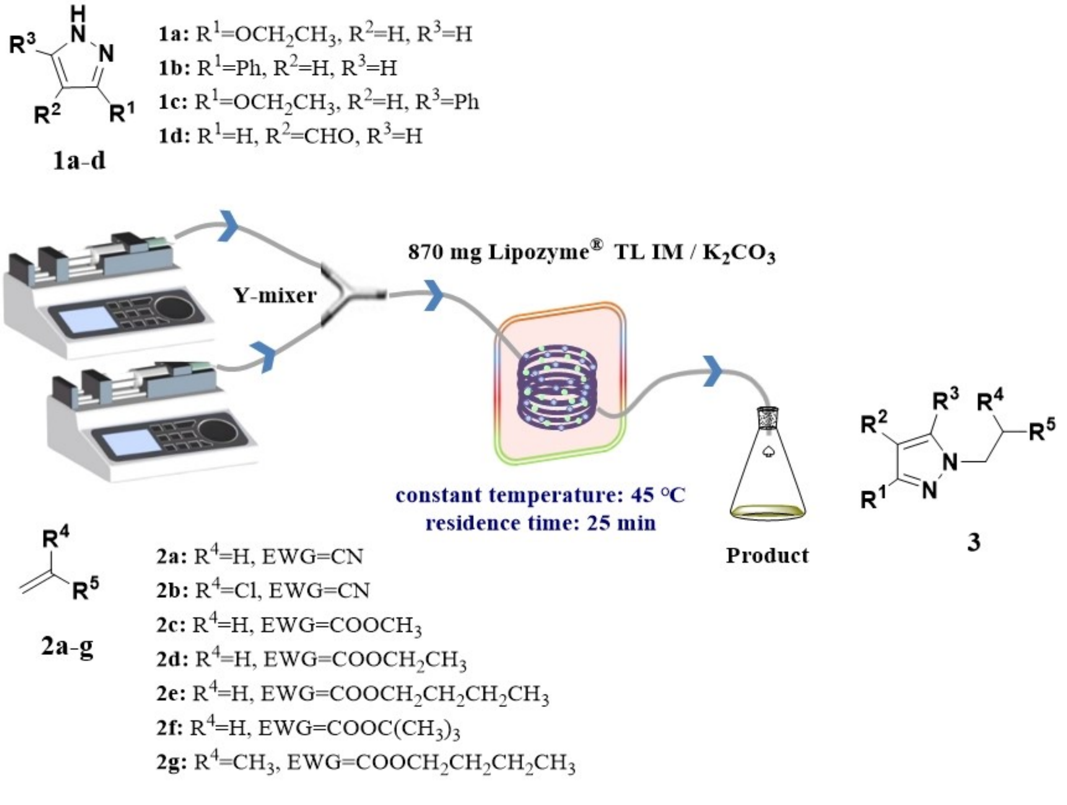

Figure 1. The equipment diagram for the synthesis of *N*-alkylated pyrazole compounds in continuous-flow microreactors catalysed by Lipozyme® TL IM/K_2CO_3.

General Procedure for the synthesis of pyrazinamide derivatives from pyrazoles

and α,β-unsaturated compounds catalyzed by Lipozyme® TL IM/K_2CO_3 in Continuous-Flow Microreactors

Method A: 5.0 mmol of pyrazole was dissolved in 10 mL acetonitrile (feed A, ~0.5 M) and 15.0 mmol α,β- unsaturated compounds were dissolved in 10 mL acetonitrile (feed B; ~1.5 M). Lipozyme® TL IM/K_2CO_3 (0.87 g) were filled in PFA reactor coil (inner diameter ID= 2.0 mm, length = 100cm.). Streams A and B were mixed together at a flow rate of 12.48 μL min^{-1} in a Y-mixer at 45 °C and the resulting stream (24.96 μL min^{-1}) was connected to a sample vial which was used to collect the final mixture. The final mixture was then evaporated, and the residue was submitted to column chromatography on silica gel (200-300 mesh). The crude product was purified by silica gel column chromatography with a petroleum ether / ethyl acetate gradient from 20:1 to 1:1. The purification was monitored by TLC. The fractions containing the main products were pooled, the solvent evaporated, and the residue analyzed by ^{1}H NMR, and ^{13}C NMR.

General Procedure for the synthesis of pyrazinamide derivatives from pyrazoles and α,β- unsaturated compounds catalyzed by Lipozyme® TL IM/K_2CO_3 under Shaker Conditions

Method B: pyrazole (5.0 mmol) and α,β- unsaturated compounds (15.0 mmol) were added to 20 mL acetonitrile. Lipozyme® TL IM/K_2CO_3 (0.87 g) were then added, and the suspension maintained at 50 °C for 20 h under Shaker Conditions (136 r·min^{-1}). The mixture was cooled and filtered. Then evaporated under reduced pressure and the residue was submitted to column chromatography on silica gel (200-300 mesh). The crude product was purified by silica gel column chromatography with a petroleum ether /ethyl acetate gradient from 20:1 to 1:1. The purification was monitored by TLC. The fractions containing the main products were pooled, the solvent evaporated, and the residue analyzed by ^{1}H NMR, and ^{13}C NMR.

Experimental data of products

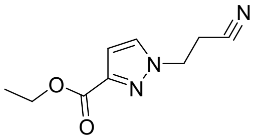

ethyl 1-(2-cyanoethyl)-1H-pyrazole-3-carboxylate (3a). White powder, 70.23% yield. ^{1}H NMR (400 MHz, Chloroform-d) δ 7.56 (d, J = 2.4 Hz, 1H), 6.83 (d, J = 2.6 Hz, 1H), 4.48 (t, J = 6.5 Hz, 2H), 4.40 (q, J = 7.1 Hz, 2H), 3.00 (t, J = 6.5 Hz, 2H), 1.39 (t, J = 7.1 Hz, 3H). ^{13}C NMR (101 MHz, Chloroform-d) δ 161.93, 145.11, 131.50, 116.68, 109.38, 61.15, 48.25, 19.45, 14.36.

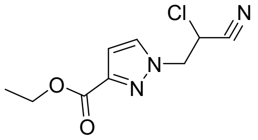

ethyl 1-(2-chloro-2-cyanoethyl)-1H-pyrazole-3-carboxylate (3b). Colorless oily liquid, 73.65% yield. ^{1}H NMR (400 MHz, DMSO-d6) δ 7.73 (d, J = 2.0 Hz, 1H), 6.99 (d, J = 2.0 Hz, 1H), 5.73 (t, J = 6.4 Hz, 1H), 5.22 – 5.08 (m, 2H), 4.37 – 4.27 (m, 2H), 1.32 (t, J = 7.1 Hz, 3H). ^{13}C NMR (101 MHz, DMSO-d6) δ 159.49, 140.05, 133.46, 116.66, 112.38, 61.73, 53.21, 42.19, 14.42.

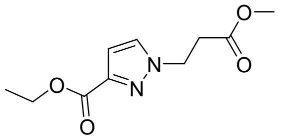

ethyl 1-(3-methoxy-3-oxopropyl)-1H-pyrazole-3-carboxylate (3c). Colorless liquid, 84.73% yield. ^{1}H NMR (400 MHz, Chloroform-*d*) δ 7.48 (d, *J* = 1.9 Hz, 1H), 6.73 (d, *J* = 1.8 Hz, 1H), 4.47 (t, *J* = 6.5 Hz, 2H), 4.36 (q, *J* = 6.6, 5.9 Hz, 2H), 3.65 (s, 3H), 2.92 (t, *J* = 6.5 Hz, 2H), 1.36 (t, *J* = 6.5 Hz, 3H). ^{13}C NMR (101 MHz, Chloroform-*d*) δ 171.26, 162.24, 144.08, 131.49, 108.72, 60.90, 51.97, 48.07, 34.52, 14.35.

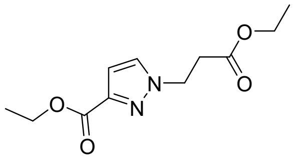

ethyl 1-(3-ethoxy-3-oxopropyl)-1H-pyrazole-3-carboxylate (3d). Colorless liquid, 83.03% yield. ^{1}H NMR (400 MHz, Chloroform-*d*) δ 7.48 (d, *J* = 2.4 Hz, 1H), 6.74 (d, *J* = 2.4 Hz, 1H), 4.47 (t, *J* = 6.5 Hz, 2H), 4.37 (q, *J* = 7.1 Hz, 2H), 4.11 (q, *J* = 7.1 Hz, 2H), 2.91 (t, *J* = 6.5 Hz, 2H), 1.36 (t, *J* = 7.1 Hz, 3H), 1.20 (t, *J* = 7.2 Hz, 3H). ^{13}C NMR (101 MHz, Chloroform-*d*) δ 170.81, 162.27, 144.04, 131.47, 108.71, 60.97, 60.90, 48.13, 34.80, 14.36, 14.07.

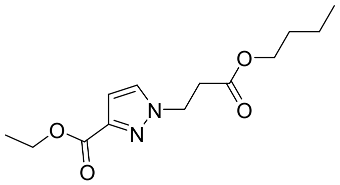

ethyl 1-(3-propoxy-3-oxopropyl) -1H-pyrazole-3-carboxylate (3e). Colorless liquid, 81.56% yield. ^{1}H NMR (400 MHz, Chloroform-*d*) δ 7.47 (d, *J* = 2.2 Hz, 1H), 6.72 (d, *J* = 2.5 Hz, 1H), 4.45 (t, *J* = 5.3 Hz, 2H), 4.35 (q, *J* = 7.1 Hz, 2H), 4.03 (t, *J* = 5.3 Hz, 2H), 2.89 (t, *J* = 5.3 Hz, 2H),

1.59 – 1.44 (m, 2H), 1.41 – 1.16 (m, 5H), 0.86 (t, *J* = 6.1 Hz, 3H). ^{13}C NMR (101 MHz, Chloroform-*d*) δ 170.84, 162.21, 144.01, 131.43, 108.68, 64.80, 60.85, 48.14, 34.76, 30.45, 18.97, 14.34, 13.57.

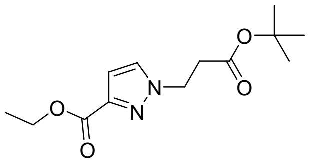

ethyl 1-(3-(tert-butoxy)-3-oxopropyl)-1H-pyrazole-3-carboxylate (3f). Colorless liquid, 79.23% yield. ^{1}H NMR (400 MHz, DMSO-d_6) δ 7.84 (d, *J* = 2.4 Hz, 1H), 6.71 (d, *J* = 2.4 Hz, 1H), 4.39 (t, *J* = 6.6 Hz, 2H), 4.25 (q, *J* = 7.1 Hz, 2H), 2.80 (t, *J* = 6.6 Hz, 2H), 1.36 (s, 9H), 1.28 (t, *J* = 7.1 Hz, 3H). ^{13}C NMR (101 MHz, DMSO-d_6) δ 170.10, 162.11, 143.00, 132.52, 108.59, 80.77, 60.48, 48.40, 35.81, 28.06, 14.64.

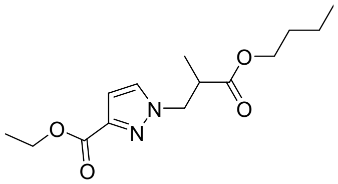

ethyl 1-(2-methyl-3-oxo-3-(pentyloxy)propyl)-1H-pyrazole-3-carboxylate (3g). Colorless liquid, 67.49% yield. ^{1}H NMR (400 MHz, Chloroform-*d*) δ 7.41 (d, *J* = 2.3 Hz, 1H), 6.72 (d, *J* = 1.9 Hz, 1H), 4.46 (dd, *J* = 13.6, 8.1 Hz, 1H), 4.36 (q, *J* = 8.0 Hz, 2H), 4.18 (dd, *J* = 13.6, 6.2 Hz, 1H), 4.03 (t, *J* = 6.6 Hz, 2H), 3.22 – 3.03 (m, 1H), 1.53 (q, *J* = 7.1 Hz, 2H), 1.41 – 1.20 (m, 5H), 1.15 (d, *J* = 7.2 Hz, 3H), 0.86 (t, *J* = 7.4 Hz, 3H). ^{13}C NMR (101 MHz, Chloroform-*d*) δ 174.07, 162.23, 144.05, 131.57, 108.60, 64.75, 60.86, 54.87, 40.67, 30.47, 18.97, 14.96, 14.36, 13.58.

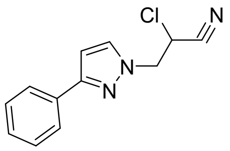

2-chloro-3-(3-phenyl-1H-pyrazol-1-yl)propanenitrile (3i). Yellow liquid, 65.65% yield. ^{1}H NMR (400 MHz, DMSO-d_6) δ 7.91 (d, *J* = 2.4 Hz, 1H), 7.87 – 7.80 (m, 2H), 7.43 (t, *J* = 7.7 Hz, 2H), 7.37 – 7.28 (m, 1H), 6.80 (d, *J* = 2.4 Hz, 1H), 5.76 (t, *J* = 6.1 Hz, 1H), 4.93 – 4.80 (m, 2H). ^{13}C NMR (101 MHz, DMSO-d_6) δ 151.88, 133.86, 133.41, 129.15, 128.26, 125.75, 116.84, 103.64, 54.08, 42.74.

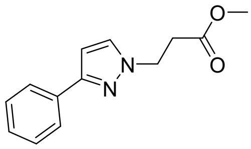

methyl 3-(3-phenyl-1H-pyrazol-1-yl)propanoate (3j). Golden yellow oily liquid, 74.61% yield. ^{1}H NMR (400 MHz, Chloroform-*d*) δ 7.85 – 7.77 (m, 2H), 7.48 (d, *J* = 2.3 Hz, 1H), 7.46 – 7.37 (m, 2H), 7.36 – 7.27 (m, 1H), 6.54 (d, *J* = 2.4 Hz, 1H), 4.47 (t, *J* = 6.6 Hz, 2H), 3.71 (s, 3H), 2.98 (t, *J* = 6.6 Hz, 2H). ^{13}C NMR (101 MHz, Chloroform-*d*) δ 171.64, 151.86, 133.51, 131.26, 128.62, 127.63, 125.59, 102.68, 51.95, 47.52, 34.81.

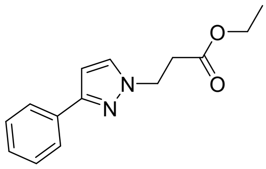

ethyl 3-(3-phenyl-1H-pyrazol-1-yl)propanoate (3k). Golden yellow oily liquid, 73.12% yield. ^{1}H NMR (400 MHz, Chloroform-*d*) δ 7.85 – 7.77 (m, 2H), 7.49 (d, *J* = 2.3 Hz, 1H), 7.47 – 7.37 (m, 2H), 7.36 – 7.26 (m, 1H), 6.54 (d, *J* = 2.3 Hz, 1H), 4.48 (t, *J* = 6.6 Hz, 2H), 4.17 (q, *J* = 7.2 Hz, 2H), 2.97 (t, *J* = 6.6 Hz, 2H), 1.26 (t, *J* = 7.2 Hz, 3H). ^{13}C NMR (101 MHz, Chloroform-*d*) δ 171.19, 151.81, 133.53, 131.24, 128.61, 127.61, 125.59, 102.64, 60.88, 47.57, 35.08, 14.16.

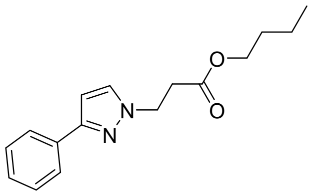

butyl 3-(3-phenyl-1H-pyrazol-1-yl)propanoate (3l). Golden yellow oily liquid, 71.56% yield. ^{1}H NMR (400 MHz, Chloroform-*d*) δ 7.85 – 7.77 (m, 2H), 7.48 (d, *J* = 2.3 Hz, 1H), 7.45 – 7.37 (m, 2H), 7.36 – 7.26 (m, 1H), 6.54 (d, *J* = 2.3 Hz, 1H), 4.48 (t, *J* = 6.6 Hz, 2H), 4.11 (t, *J* = 6.7 Hz, 2H), 2.97 (t, *J* = 6.6 Hz, 2H), 1.66 – 1.53 (m, 2H), 1.44 – 1.29 (m, 2H), 0.92 (t, *J* = 7.4 Hz, 3H). ^{13}C NMR (101 MHz, Chloroform-*d*) δ 171.27, 151.82, 133.53, 131.21, 128.60, 127.60, 125.59, 102.65, 64.78, 47.61, 35.07, 30.57, 19.08, 13.69.

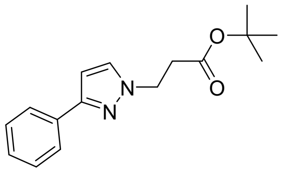

tert-butyl 3-(3-phenyl-1H-pyrazol-1-yl)propanoate (3m). Golden yellow oily liquid, 69.40% yield. ^{1}H NMR (400 MHz, Chloroform-*d*) δ 7.85 – 7.78 (m, 2H), 7.48 (d, *J* = 2.3 Hz, 1H), 7.45 – 7.37 (m, 2H), 7.36 – 7.26 (m, 1H), 6.54 (d, *J* = 2.3 Hz, 1H), 4.44 (t, *J* = 6.7 Hz, 2H), 2.88 (t, *J* = 6.7 Hz, 2H), 1.45 (s, 9H). ^{13}C NMR (101 MHz, Chloroform-*d*) δ 170.40, 151.67, 133.58, 131.10, 128.60, 127.57, 125.59, 102.60, 81.20, 47.82, 36.28, 28.05.

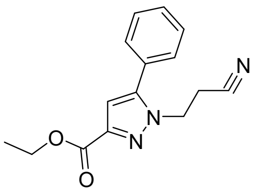

ethyl 1-(2-cyanoethyl)-5-phenyl-1H-pyrazole-3-carboxylate (3o). White solid, 58.77% yield. ^{1}H NMR (400 MHz, Chloroform-*d*) δ 7.57 – 7.39 (m, 5H), 6.84 (s, 1H), 4.49 – 4.39 (m, 4H), 3.01 (t, *J* = 6.9 Hz, 2H), 1.42 (t, *J* = 7.1 Hz, 3H). ^{13}C NMR (101 MHz, Chloroform-*d*) δ 162.07, 145.91, 144.19, 129.67, 129.25, 129.16, 128.74, 116.58, 109.29, 61.21, 45.11, 18.85, 14.41.

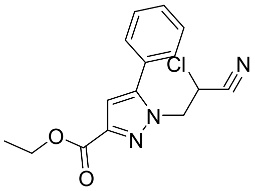

ethyl 1-(2-chloro-2-cyanoethyl)-5-phenyl-1H-pyrazole-3-carboxylate (3p). White solid, 62.65% yield. ^{1}H NMR (400 MHz, DMSO-d_6) δ 7.95 – 7.87 (m, 2H), 7.52 – 7.42 (m, 3H), 7.42 – 7.33 (m, 1H), 5.80 (t, *J* = 6.3 Hz, 1H), 5.30 – 5.13 (m, 2H), 4.41 – 4.31 (m, 2H), 1.35 (t, *J* = 7.1

Hz, 3H). ^{13}C NMR (101 MHz, DMSO-d_6) δ 159.42, 150.83, 135.00, 132.04, 129.30, 128.98, 125.93, 116.72, 109.16, 61.86, 53.37, 42.25, 14.47.

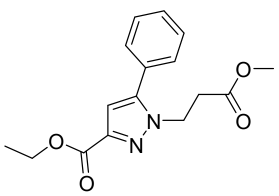

ethyl 1-(3-methoxy-3-oxopropyl)-5-phenyl-1H-pyrazole-3-carboxylate (3q). Pale yellow nearly colorless liquid, 70.61% yield. ^1H NMR (400 MHz, Chloroform-*d*) δ 7.85 – 7.77 (m, 2H), 7.46 – 7.38 (m, 2H), 7.38 – 7.30 (m, 1H), 7.15 (s, 1H), 4.92 (t, *J* = 7.3 Hz, 2H), 4.40 (q, *J* = 7.1 Hz, 2H), 3.71 (s, 3H), 2.98 (t, *J* = 7.3 Hz, 2H), 1.43 (t, *J* = 7.1 Hz, 3H). ^{13}C NMR (101 MHz, Chloroform-*d*) δ 171.30, 159.69, 150.15, 133.54, 132.46, 128.70, 128.10, 125.54, 108.13, 61.22, 51.86, 47.30, 34.71, 14.27.

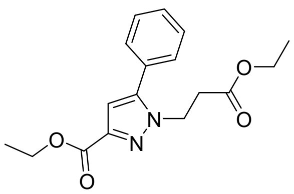

ethyl 1-(3-ethoxy-3-oxopropyl)-5-phenyl-1H-pyrazole-3-carboxylate (3r). Pale yellow nearly colorless liquid, 68.12% yield. ^1H NMR (400 MHz, Chloroform-*d*) δ 7.85 – 7.78 (m, 2H), 7.45 – 7.38 (m, 2H), 7.38 – 7.26 (m, 1H), 7.15 (s, 1H), 4.92 (t, *J* = 7.3 Hz, 2H), 4.40 (q, *J* = 7.1 Hz, 2H), 4.17 (q, *J* = 7.1 Hz, 2H), 2.97 (t, *J* = 7.3 Hz, 2H), 1.43 (t, *J* = 7.1 Hz, 3H), 1.26 (t, *J* = 7.1 Hz, 3H). ^{13}C NMR (101 MHz, Chloroform-*d*) δ 170.85, 159.68, 150.10, 133.55, 132.49, 128.69, 128.07, 125.54, 108.12, 61.20, 60.73, 47.36, 34.95, 14.27, 14.17.

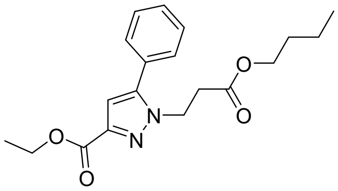

ethyl 1-(3-butoxy-3-oxopropyl)-5-phenyl-1H-pyrazole-3-carboxylate (3s). Pale yellow nearly colorless liquid, 65.56% yield. ^1H NMR (400 MHz, Chloroform-*d*) δ 7.87 – 7.78 (m, 2H), 7.47 – 7.29 (m, 3H), 7.15 (s, 1H), 4.92 (t, *J* = 7.3 Hz, 2H), 4.40 (q, *J* = 7.1 Hz, 2H), 4.11 (t, *J* = 6.7 Hz, 2H), 2.98 (t, *J* = 7.3 Hz, 2H), 1.66 – 1.55 (m, 2H), 1.47 – 1.22 (m, 5H), 0.92 (t, *J* = 7.4 Hz, 3H). ^{13}C NMR (101 MHz, Chloroform-*d*) δ 170.94, 159.68, 150.10, 133.53, 132.48, 128.68, 128.07, 125.54, 108.12, 64.65, 61.20, 47.38, 34.92, 30.60, 19.10, 14.27, 13.69.

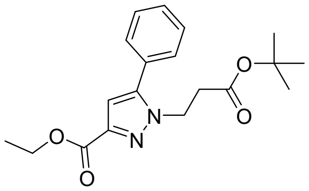

ethyl 1-(3-(tert-butoxy)-3-oxopropyl)-5-phenyl-1H-pyrazole-3-carboxylate (3t). Pale yellow nearly colorless liquid, 64.40% yield. ^1H NMR (400 MHz, Chloroform-*d*) δ 7.85 – 7.80 (m, 2H), 7.47 – 7.31 (m, 3H), 7.15 (s, 1H), 4.88 (t, *J* = 7.3 Hz, 2H), 4.40 (q, *J* = 7.2, 6.8 Hz, 2H),

2.90 (t, J = 7.3 Hz, 2H), 1.46 – 1.43 (m, 12H). ^{13}C NMR (101 MHz, Chloroform-d) δ 170.04, 159.67, 149.99, 133.50, 132.50, 128.68, 128.04, 125.55, 108.11, 80.93, 61.16, 47.62, 36.08, 28.04, 14.27.

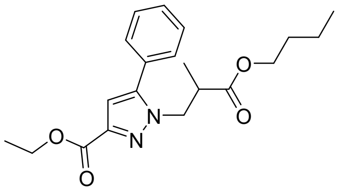

ethyl 1-(3-butoxy-2-methyl-3-oxopropyl)-5-phenyl-1H-pyrazole-3-carboxylate (3u). Pale yellow nearly colorless liquid, 53.41% yield. ^{1}H NMR (400 MHz, Chloroform-d) δ 9.22 (s, 1H), 8.37 (s, 1H), 8.08 (br s, 1H, -NH), 3.70 (t, J = 4.5 Hz, 4H), 3.55 (q, J = 6.0 Hz, 2H), 2.61 (s, 3H), 2.58 (t, J = 4.1 Hz, 2H), 2.48 (t, J = 6.0 Hz, 4H). ^{13}C NMR (101 MHz, Chloroform-d) δ 163.35, 156.89, 143.28, 142.40, 141.93, 66.97, 57.11, 53.42, 35.80, 21.81.

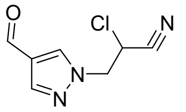

2-chloro-3-(4-formyl-1H-pyrazol-1-yl)propanenitrile (3w). Yellow liquid, 75.65% yield. ^{1}H NMR (400 MHz, DMSO-d_6) δ 9.85 (s, 1H), 8.58 (s, 1H), 8.13 (s, 1H), 5.78 (t, J = 5.9 Hz, 1H), 4.93 (d, J = 6.0 Hz, 2H). ^{13}C NMR (101 MHz, DMSO-d_6) δ 185.47, 141.27, 136.93, 124.51, 116.53, 54.12, 42.39.

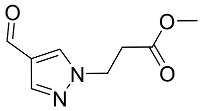

methyl 3-(4-formyl-1H-pyrazol-1-yl)propanoate (3x). Colorless oily liquid, 87.56% yield. ^{1}H NMR (400 MHz, DMSO-d_6) δ 9.79 (s, 1H), 8.47 (s, 1H), 7.99 (s, 1H), 4.43 (t, J = 6.7 Hz, 2H), 3.59 (s, 3H), 2.94 (t, J = 6.7 Hz, 2H). ^{13}C NMR (101 MHz, DMSO-d_6) δ 185.17, 171.39, 140.44, 135.45, 124.11, 52.04, 47.82, 33.98.

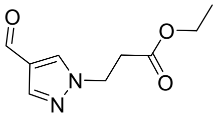

ethyl 3-(4-formyl-1H-pyrazol-1-yl)propanoate (3y). Colorless oily liquid, 83.35% yield. ^{1}H NMR (400 MHz, DMSO-d_6) δ 9.79 (s, 1H), 8.46 (s, 1H), 7.99 (s, 1H), 4.43 (t, J = 6.6 Hz, 2H), 4.05 (q, J = 7.1 Hz, 2H), 2.92 (t, J = 6.6 Hz, 2H), 1.14 (t, J = 7.1 Hz, 3H). ^{13}C NMR (101 MHz, DMSO-d_6) δ 185.14, 170.87, 140.42, 135.46, 124.10, 60.67, 47.87, 34.21, 14.40.

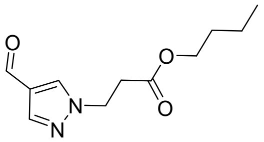

butyl 3-(4-formyl-1H-pyrazol-1-yl)propanoate (3z). Colorless oily liquid, 81.02% yield. ^{1}H NMR (400 MHz, DMSO-d_6) δ 9.79 (s, 1H), 8.46 (s, 1H), 7.99 (s, 1H), 4.43 (t, J = 6.6 Hz, 2H), 4.01 (t, J = 6.6 Hz, 2H), 2.92 (t, J = 6.6 Hz, 2H), 1.56 – 1.44 (m, 2H), 1.33 – 1.19 (m, 2H), 0.85 (t, J = 7.4 Hz, 3H). ^{13}C NMR (101 MHz, DMSO-d_6) δ 185.12, 170.93, 140.41, 135.44, 124.11, 64.33, 47.91, 34.21, 30.54, 18.97, 13.94.

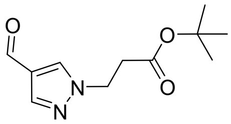

tert-butyl 3-(4-formyl-1H-pyrazol-1-yl)propanoate (3aa). Colorless oily liquid, 76.41% yield. ^{1}H NMR (400 MHz, DMSO-d_6) δ 9.79 (s, 1H), 8.45 (s, 1H), 7.99 (s, 1H), 4.38 (t, J = 6.6 Hz, 2H), 2.82 (t, J = 6.6 Hz, 2H), 1.35 (s, 9H). ^{13}C NMR (101 MHz, DMSO-d_6) δ 185.14, 170.07, 140.38, 135.43, 124.06, 80.82, 48.10, 35.41, 28.07.

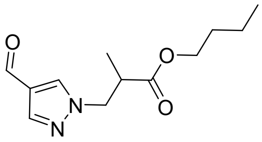

butyl 3-(4-formyl-1H-pyrazol-1-yl)-2-methylpropanoate (3ab). White solid, 71.02% yield. ^{1}H NMR (400 MHz, DMSO-d_6) δ 9.80 (s, 1H), 8.45 (s, 1H), 7.99 (s, 1H), 4.41 (dd, J = 13.6, 7.7 Hz, 1H), 4.29 (dd, J = 13.6, 6.3 Hz, 1H), 4.00 (t, J = 6.5 Hz, 2H), 3.18 – 3.00 (m, 1H), 1.55 – 1.43 (m, 2H), 1.32 – 1.18 (m, 2H), 1.07 (d, J = 7.1 Hz, 3H), 0.84 (t, J = 7.4 Hz, 3H). ^{13}C NMR (101 MHz, DMSO-d_6) δ 185.11, 173.71, 140.48, 135.80, 124.10, 64.39, 54.25, 40.04, 30.51, 18.97, 14.69, 13.92.

3a

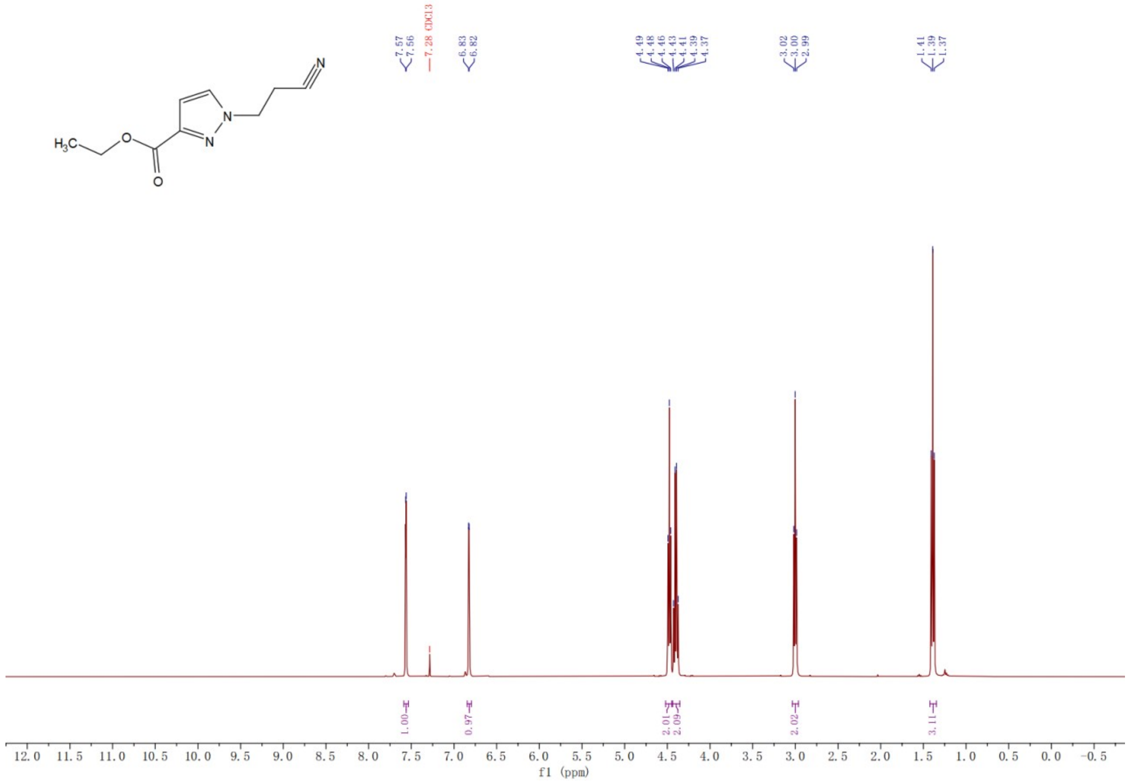
7.57
7.56
7.28 CDCl3
6.83
6.82
4.49
4.48
4.46
4.43
4.41
4.39
4.37
3.02
3.00
2.99
1.41
1.39
1.37
H3C
O
O
N
N
N
1.00
0.97
2.01
2.09
2.02
3.11
12.0 11.5 11.0 10.5 10.0 9.5 9.0 8.5 8.0 7.5 7.0 6.5 6.0 5.5 5.0 4.5 4.0 3.5 3.0 2.5 2.0 1.5 1.0 0.5 0.0 -0.5
f1 (ppm)

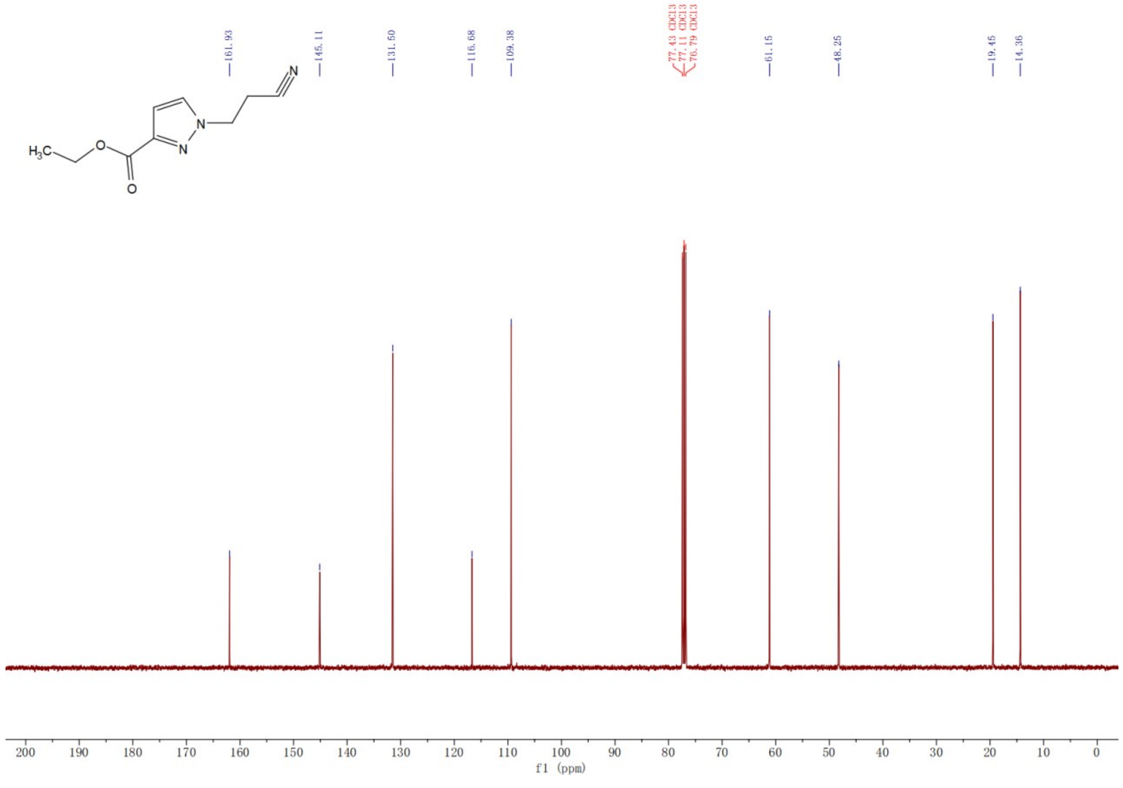
161.93
145.11
131.50
116.68
109.38
77.43 CDCl3
77.11 CDCl3
76.79 CDCl3
61.15
48.25
19.45
14.36
H3C
O
O
N
N
N
200 190 180 170 160 150 140 130 120 110 100 90 80 70 60 50 40 30 20 10 0
f1 (ppm)

3b

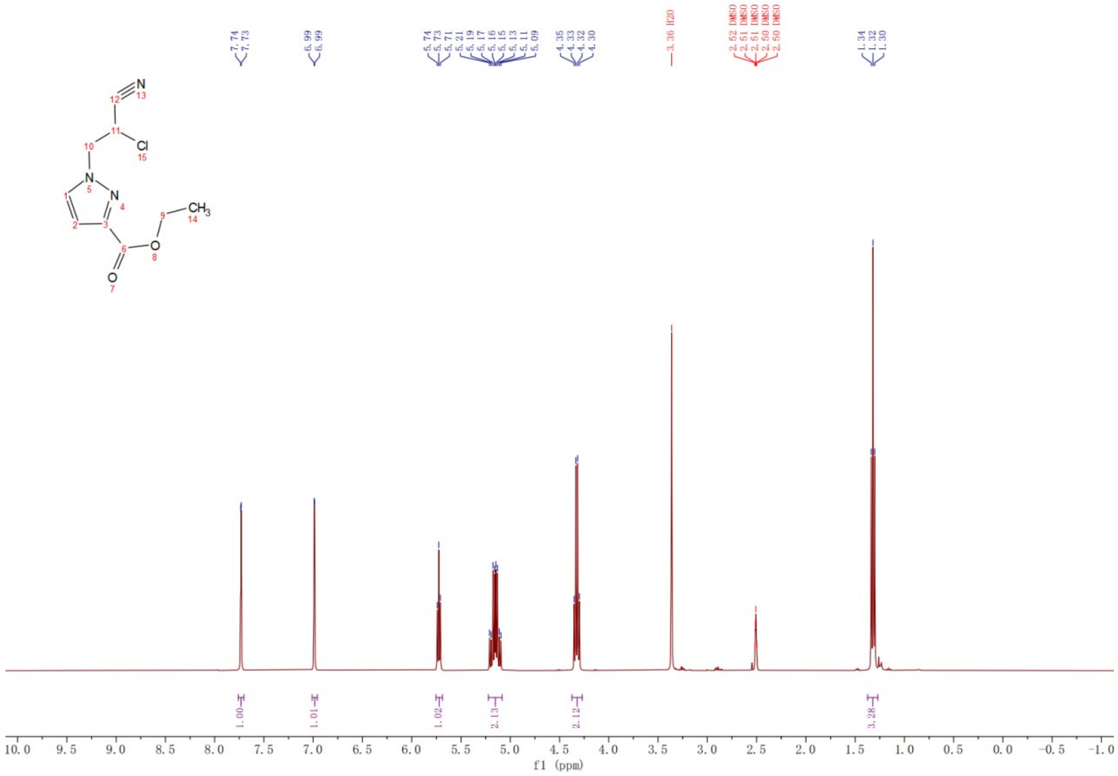
7.74
7.73
6.99
6.99
5.74
5.73
5.71
5.21
5.19
5.17
5.16
5.15
5.13
5.11
5.09
4.35
4.33
4.32
4.30
3.36 H2O
2.52 DMSO
2.51 DMSO
2.51 DMSO
2.50 DMSO
2.50 DMSO
1.34
1.32
1.30
1.00
1.01
1.02
2.13
2.12
3.28
f1 (ppm)

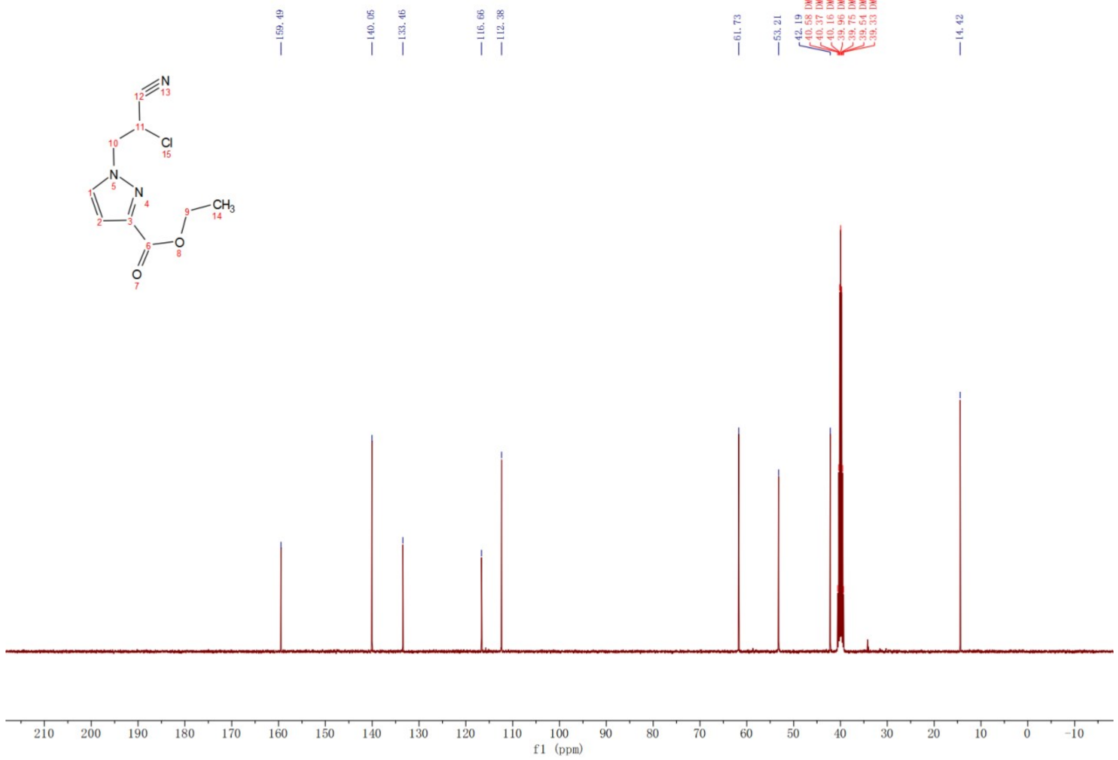
159.49
140.05
133.46
116.66
112.38
61.73
53.21
42.19
40.58 DMSO
40.37 DMSO
40.16 DMSO
39.96 DMSO
39.75 DMSO
39.54 DMSO
39.33 DMSO
14.42
f1 (ppm)

3c

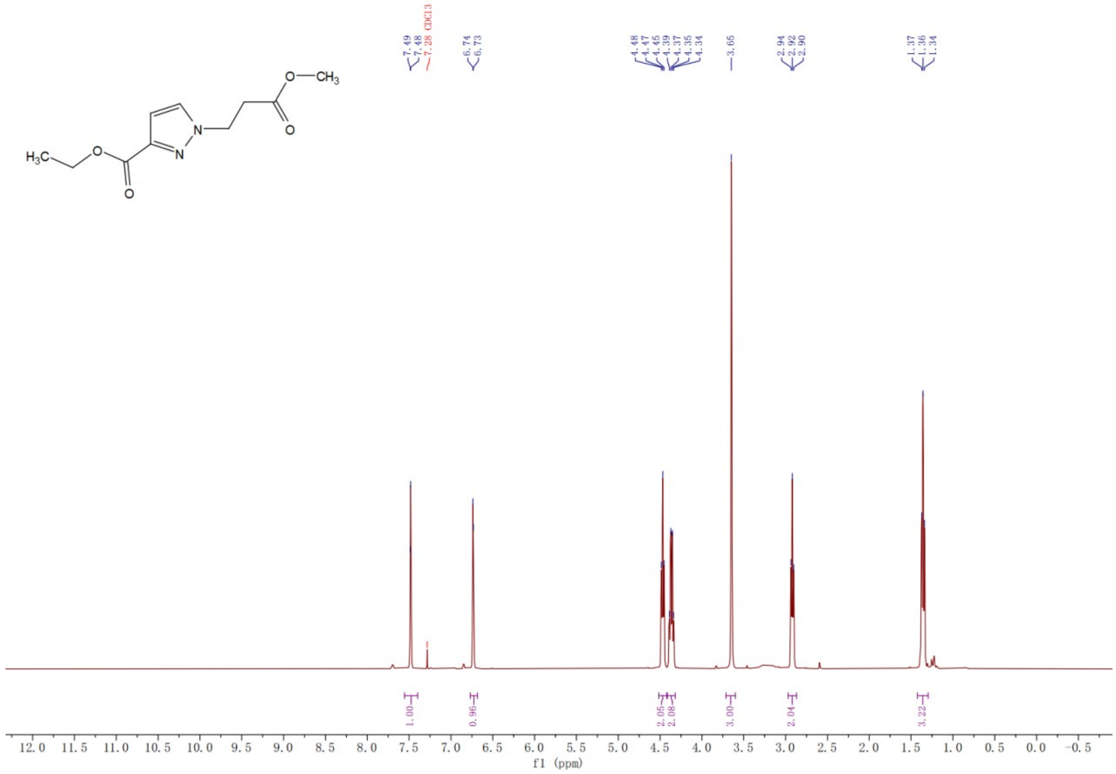

7.49
7.48
7.28 CDCl3
6.74
6.73
4.48
4.47
4.45
4.39
4.37
4.35
4.34
3.65
2.94
2.92
2.90
1.37
1.36
1.34
1.00
0.96
2.05
2.08
3.00
2.04
3.22
f1 (ppm)

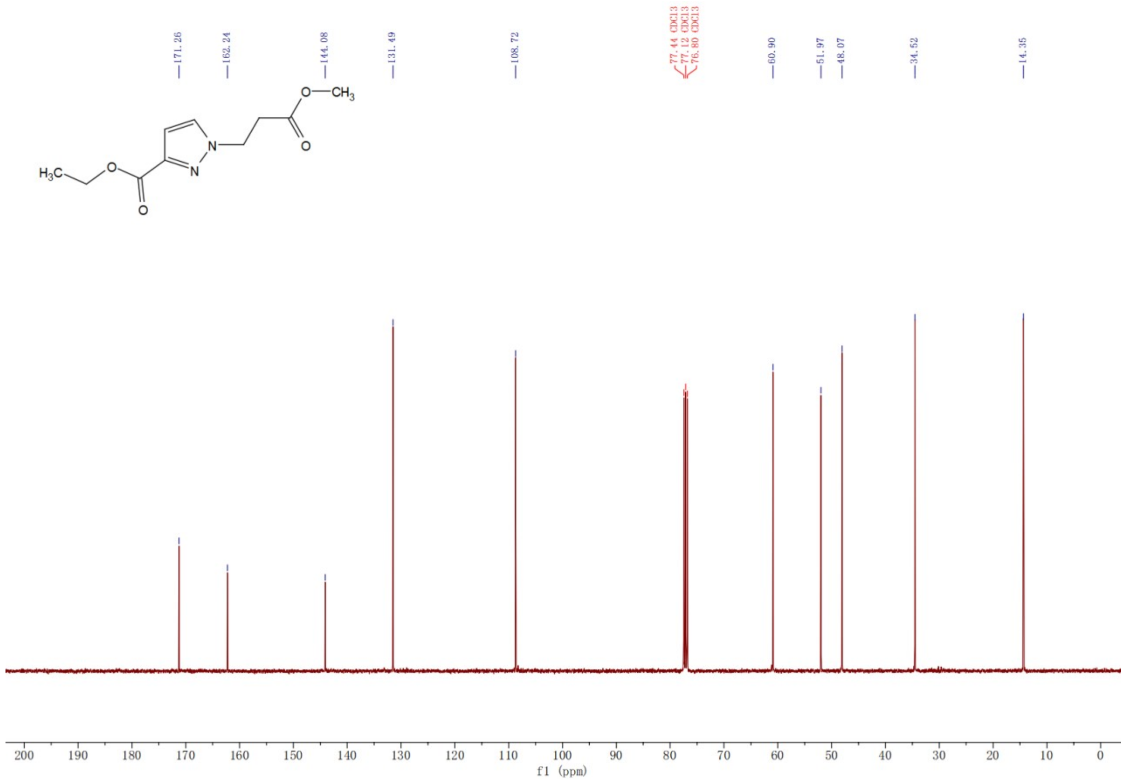

171.26
162.24
144.08
131.49
108.72
77.44 CDCl3
77.12 CDCl3
76.80 CDCl3
60.90
51.97
48.07
34.52
14.35
f1 (ppm)

3d

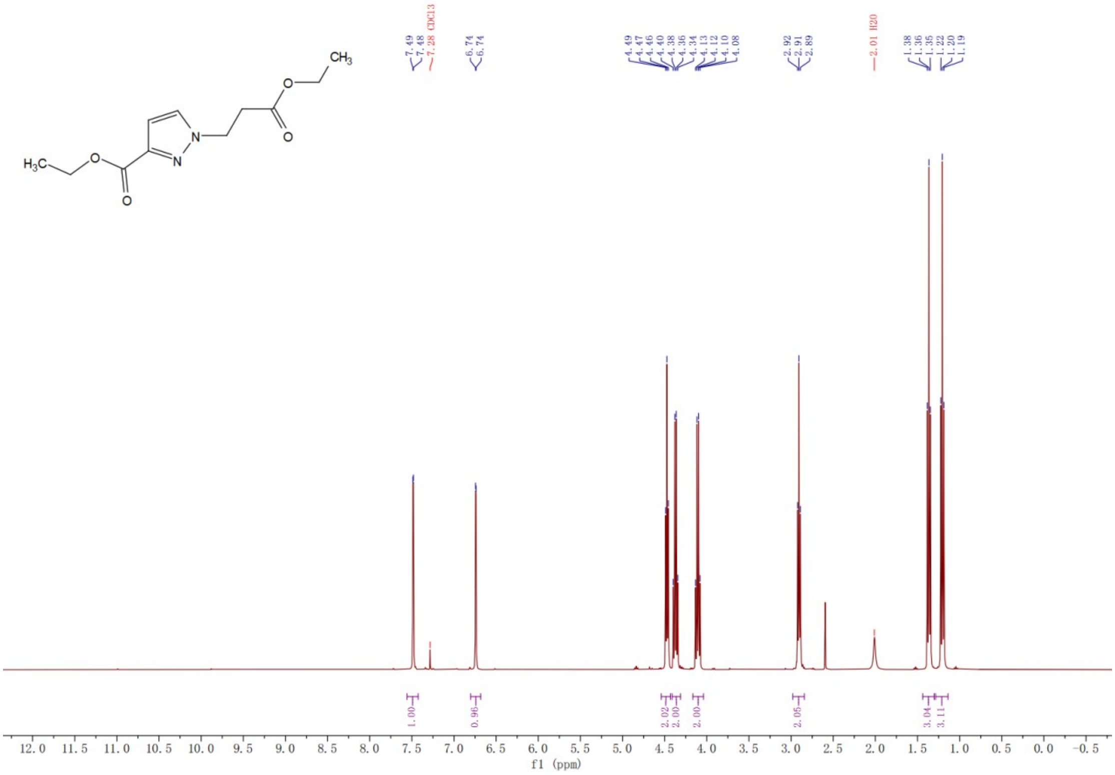

7.49
7.48
7.28 CDCl3
6.74
6.74
4.49
4.47
4.46
4.40
4.38
4.36
4.34
4.13
4.12
4.10
4.08
2.92
2.91
2.89
2.01 H2O
1.38
1.36
1.35
1.22
1.20
1.19
1.00
0.96
2.02
2.00
2.00
2.05
3.04
3.11
f1 (ppm)

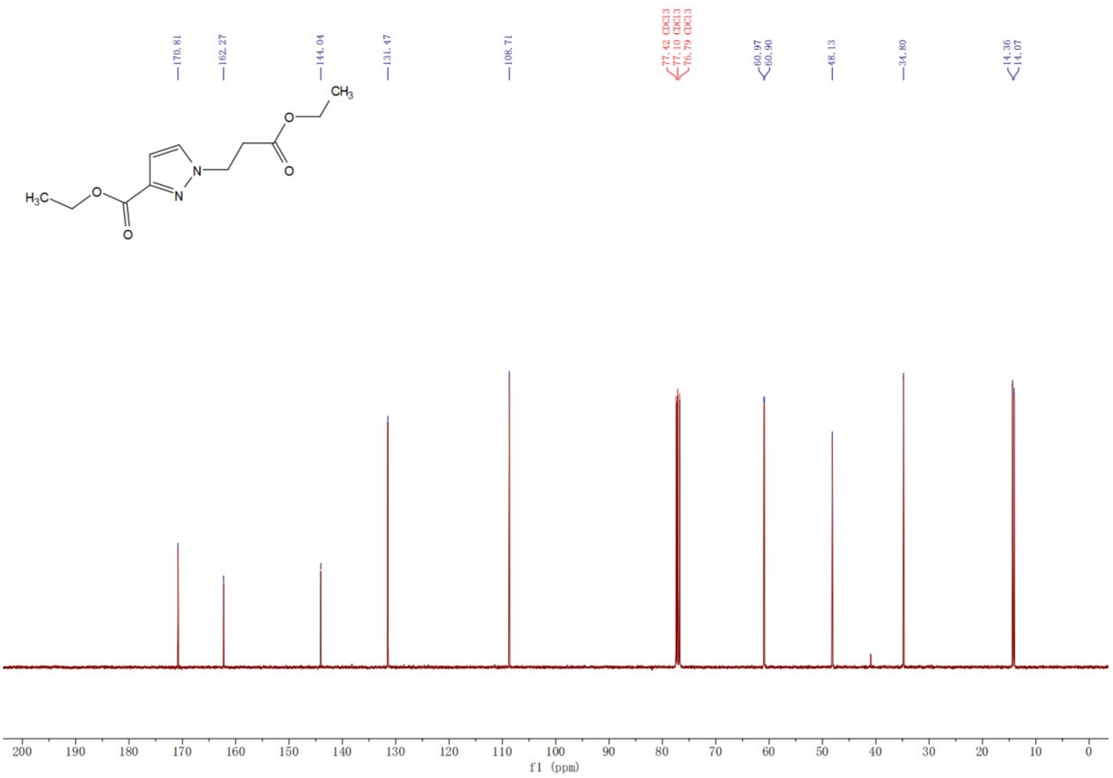

170.81
162.27
144.04
131.47
108.71
77.42 CDCl3
77.10 CDCl3
76.79 CDCl3
60.97
60.90
48.13
34.80
14.36
14.07
f1 (ppm)

3e

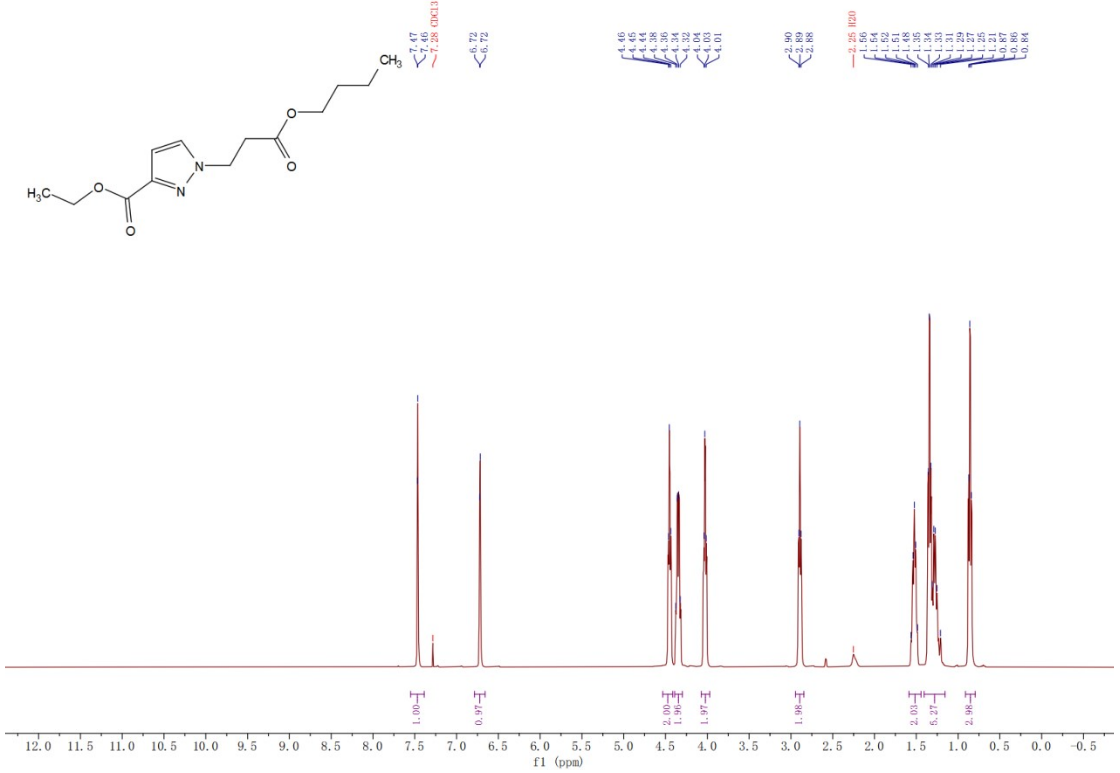

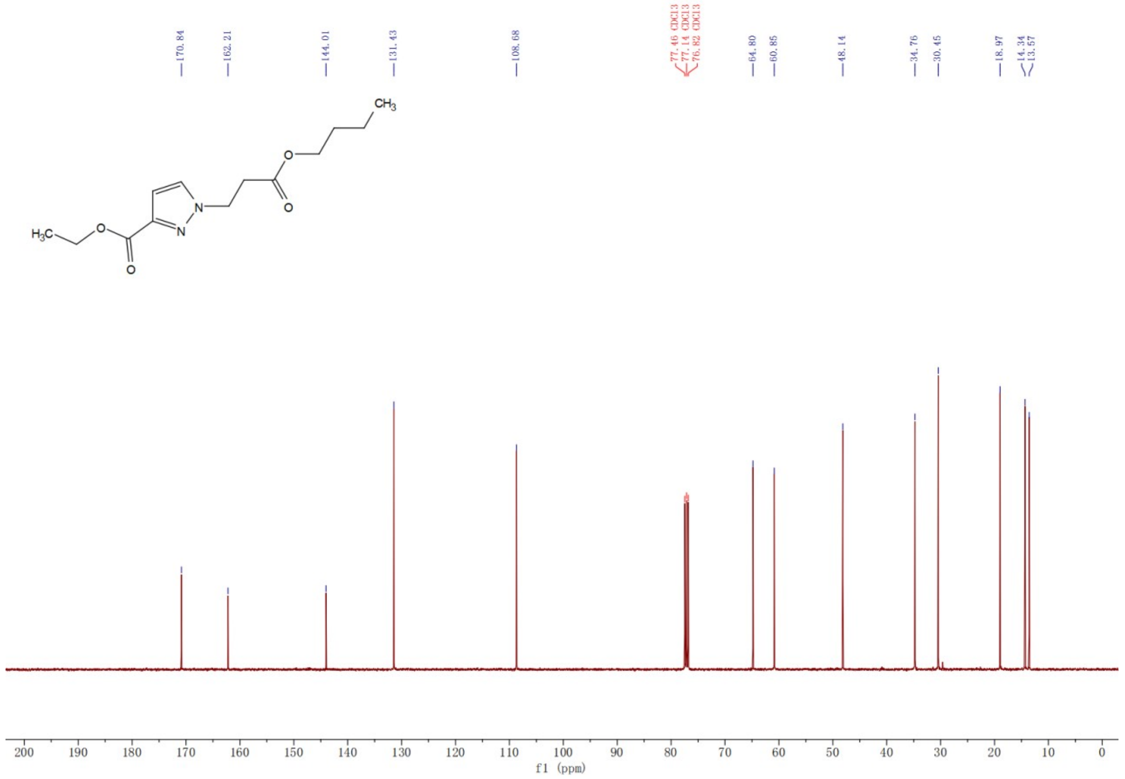

3f

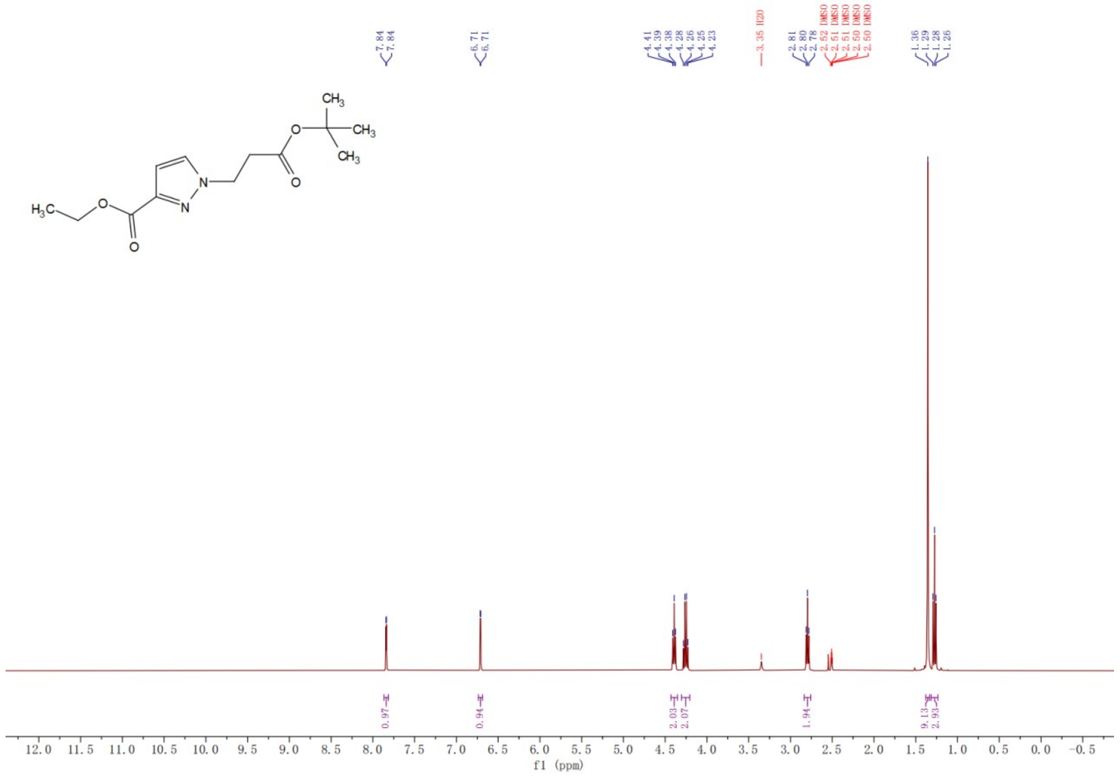

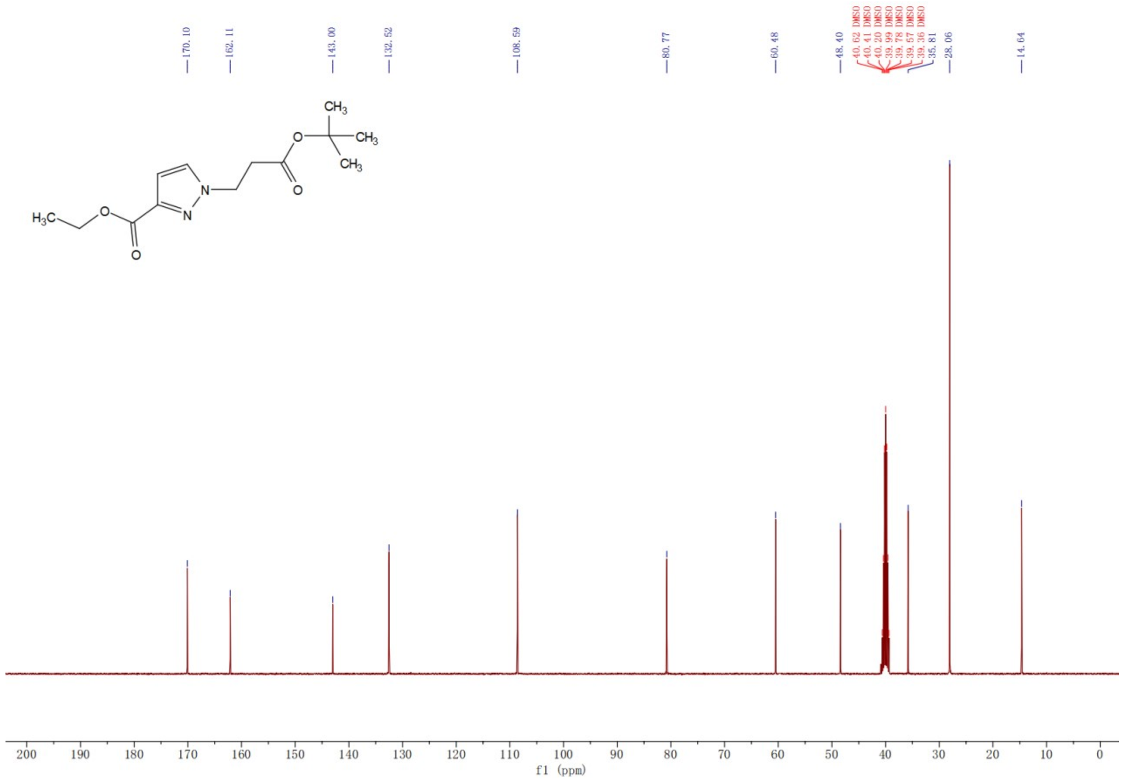

3g

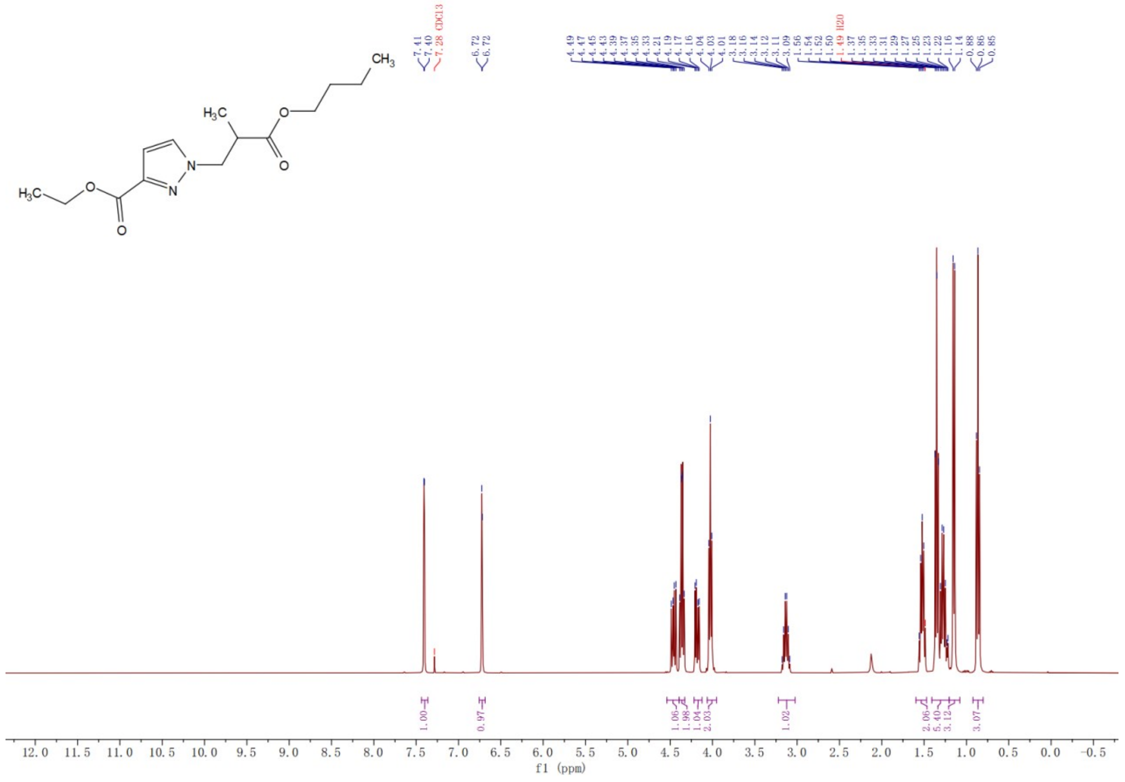

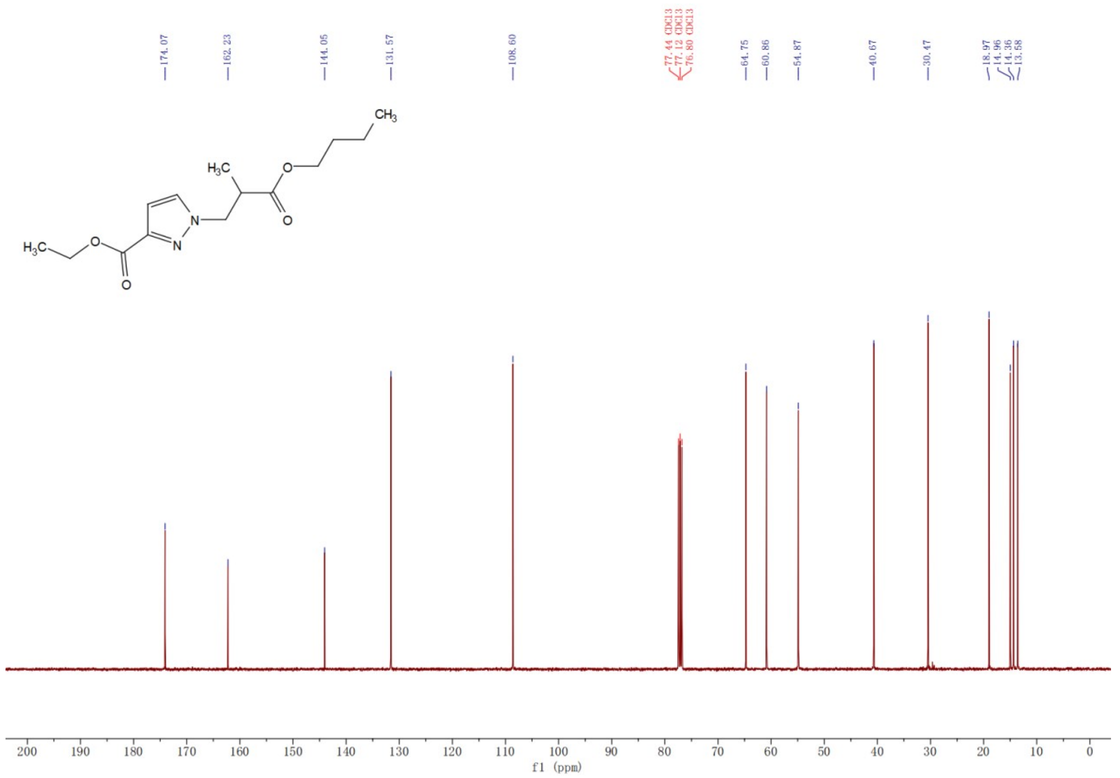

3i

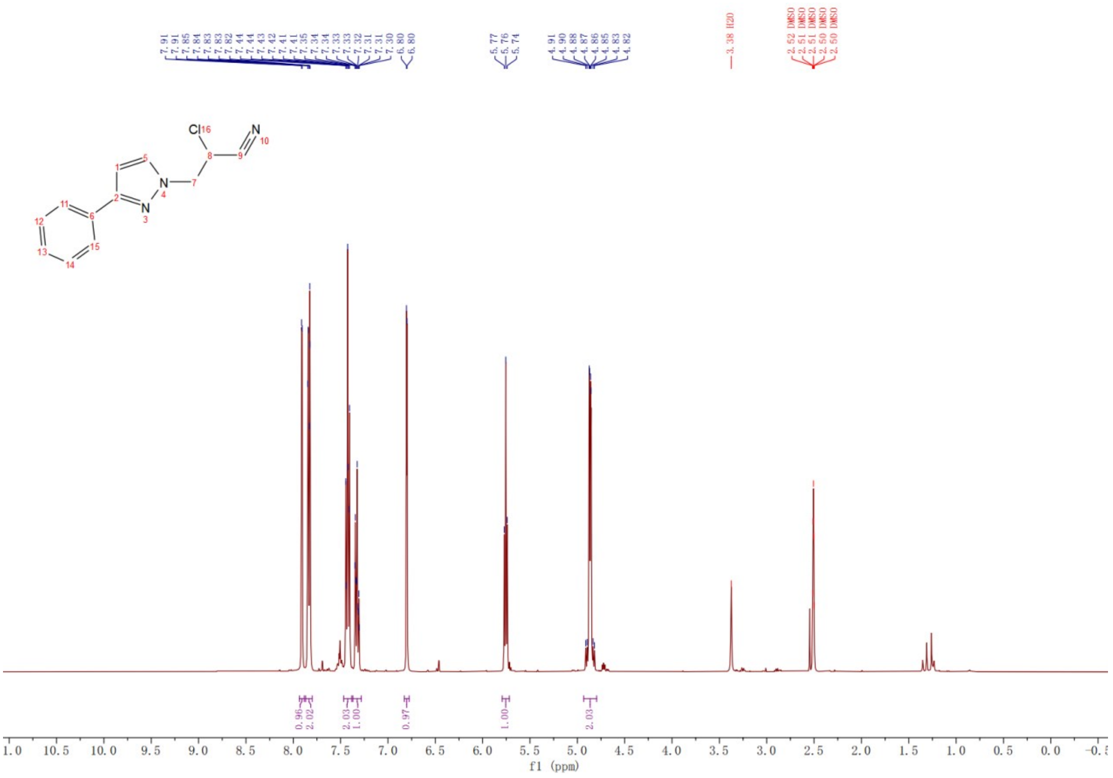

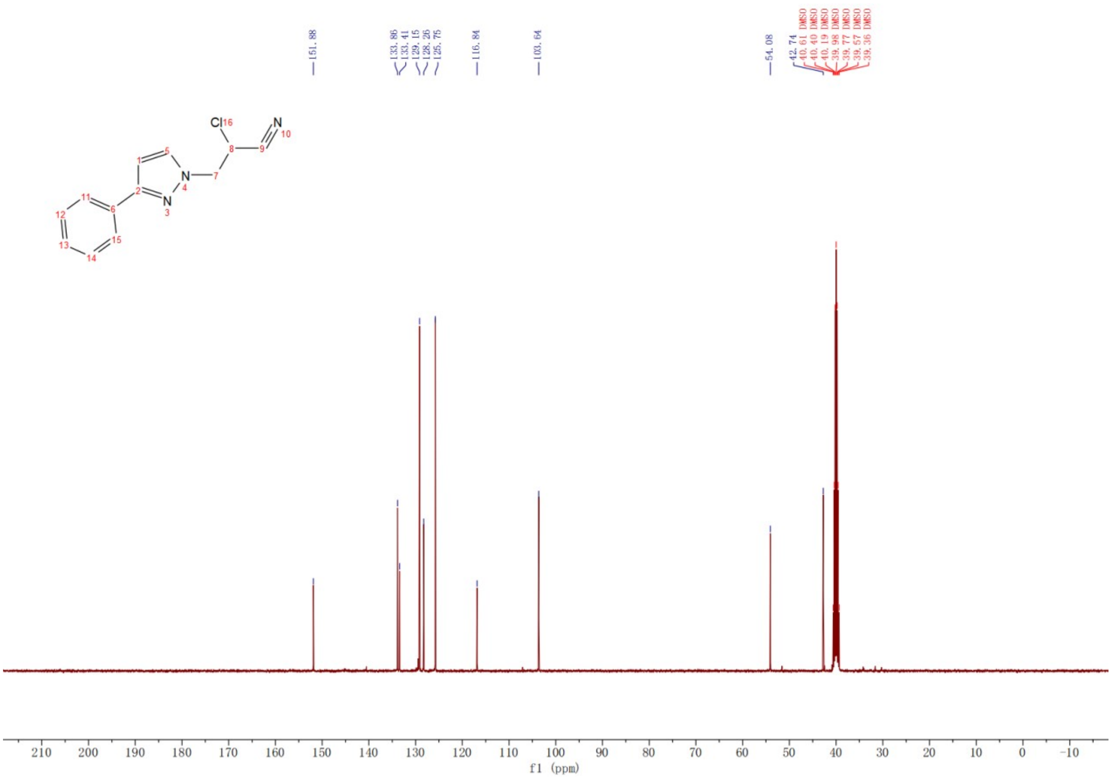

3j

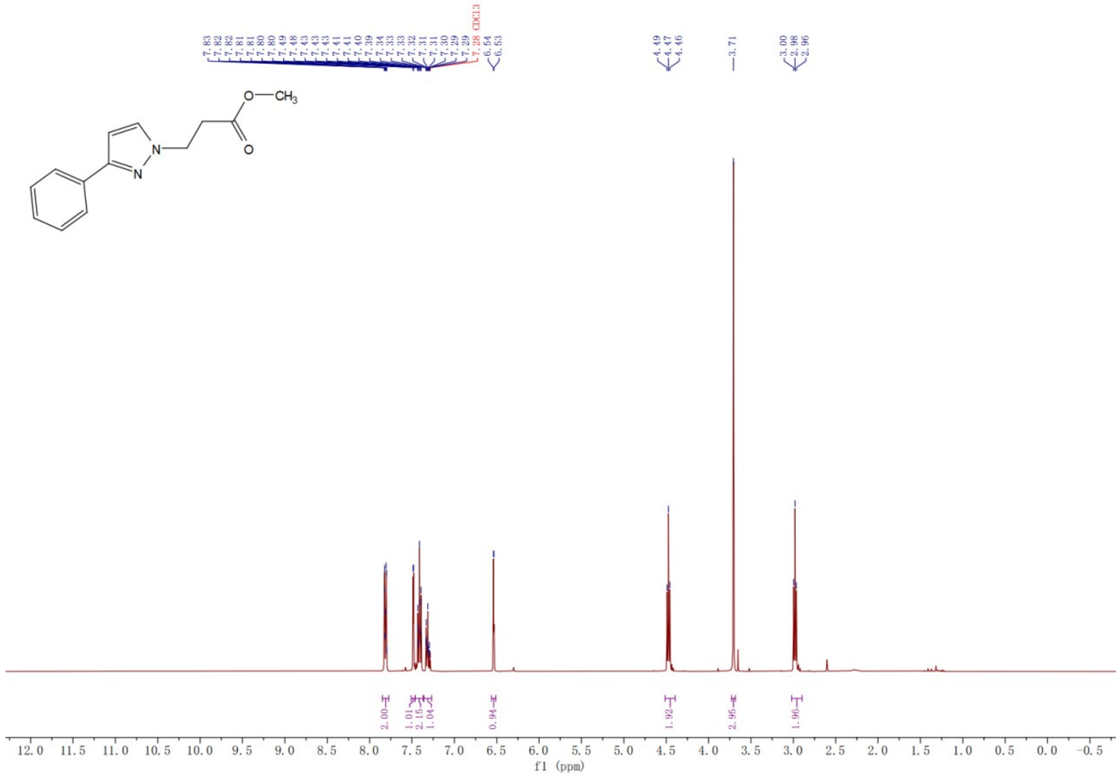

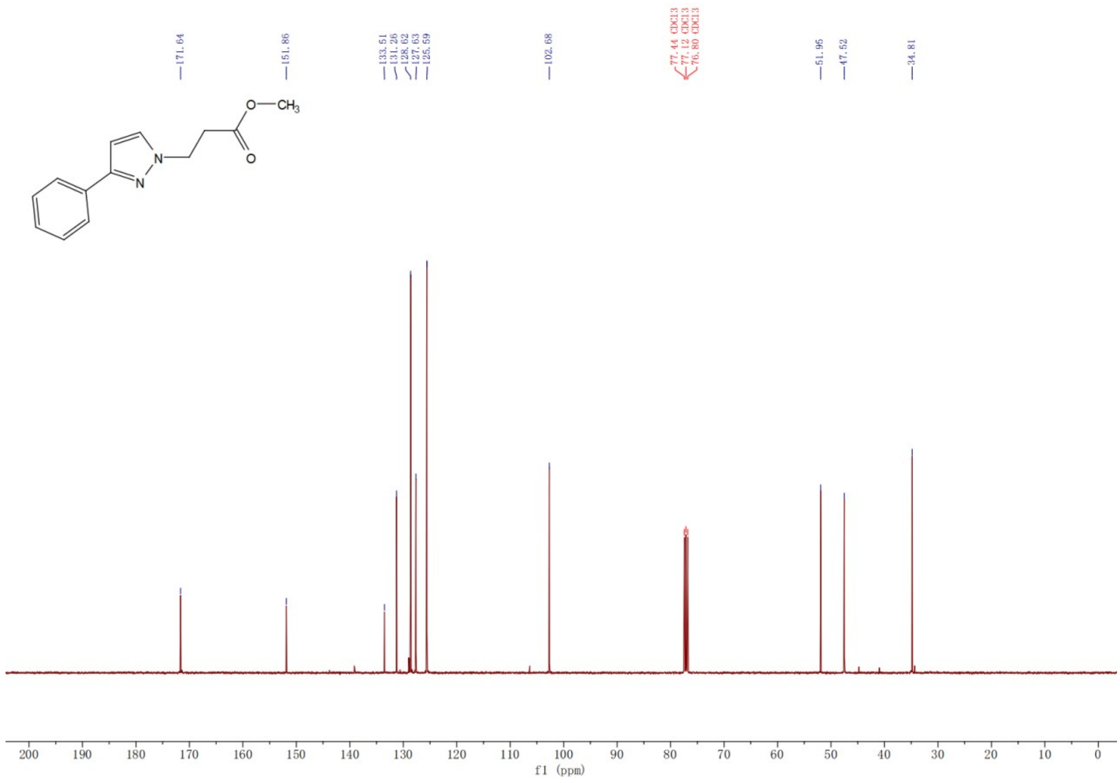

3k

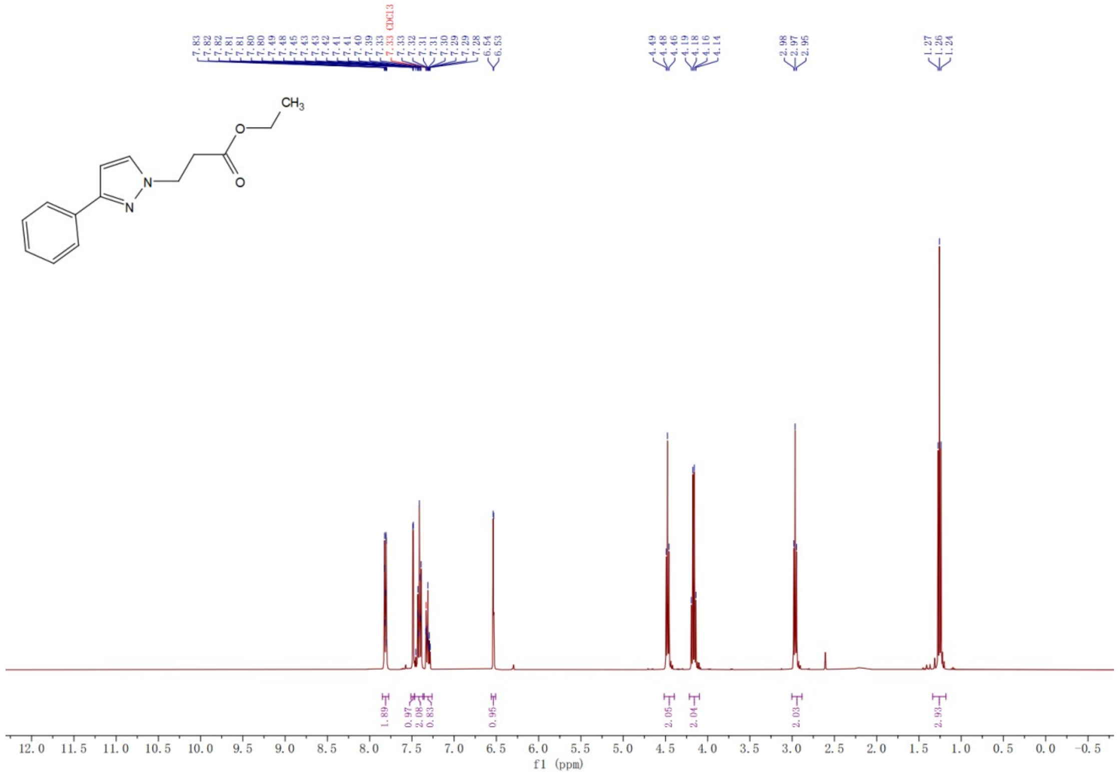

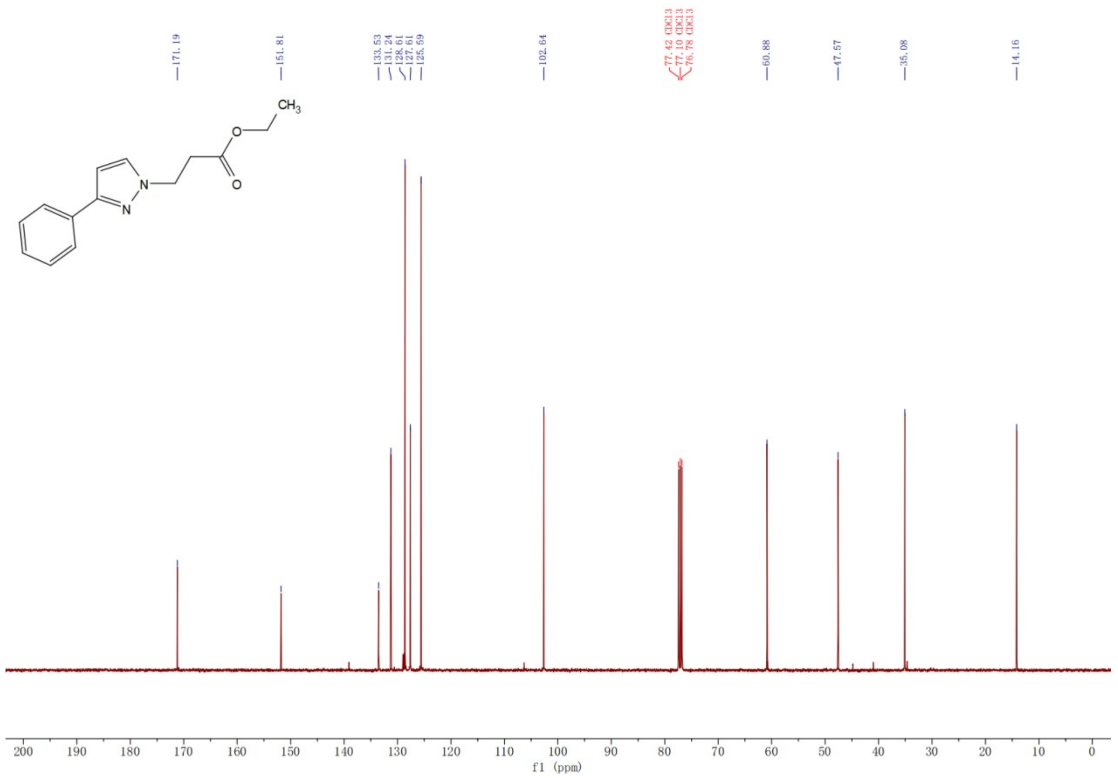

31

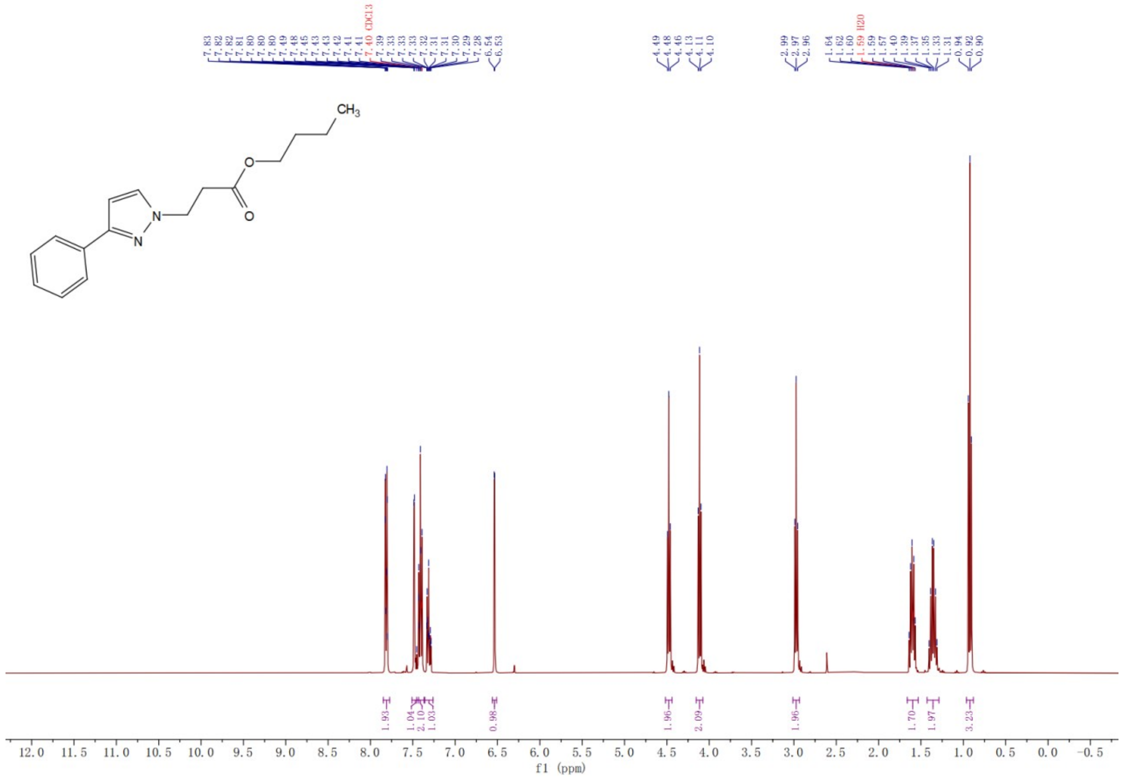
7.83
7.82
7.82
7.81
7.80
7.80
7.80
7.49
7.48
7.45
7.43
7.43
7.42
7.41
7.41
7.40 CDCl3
7.39
7.33
7.33
7.33
7.33
7.32
7.31
7.31
7.30
7.29
7.28
6.54
6.53
4.49
4.48
4.46
4.13
4.11
4.10
2.99
2.97
2.96
1.64
1.62
1.60
1.59 H2O
1.59
1.57
1.40
1.39
1.37
1.35
1.33
1.31
0.94
0.92
0.90
CH3
O
N
N
1.93
1.04
2.10
1.03
0.98
1.96
2.09
1.96
1.70
1.97
3.23
f1 (ppm)

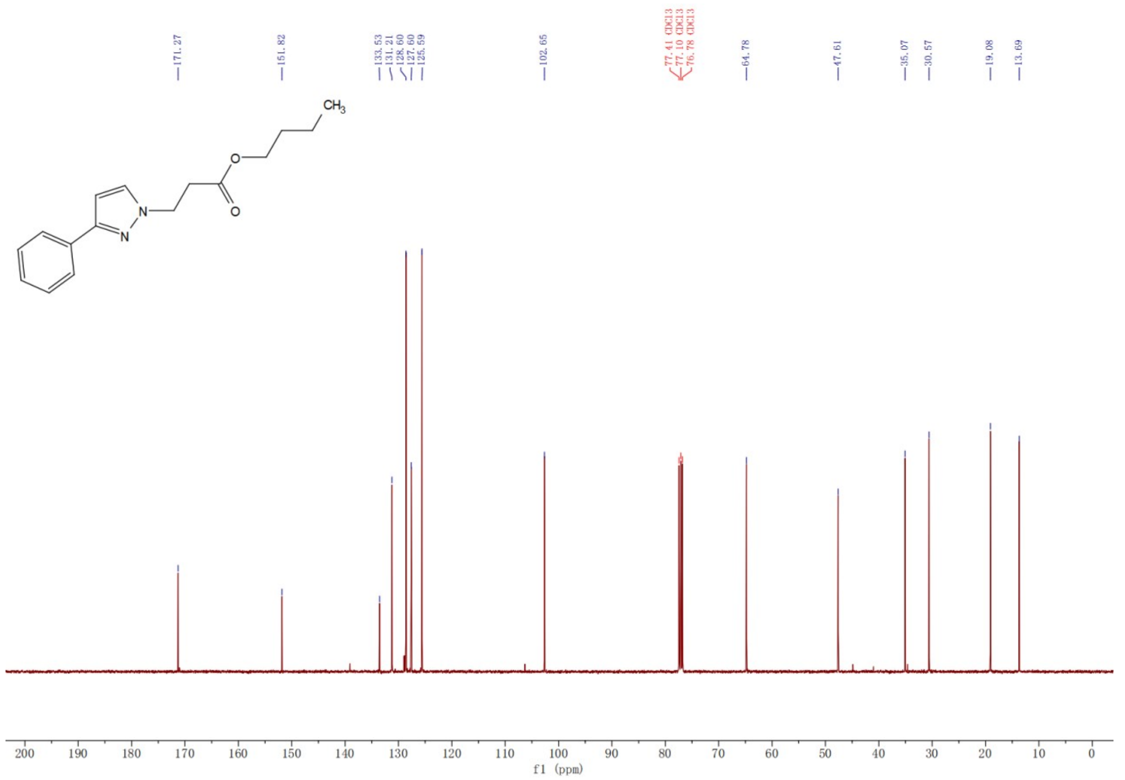
171.27
151.82
133.53
131.21
128.60
127.60
125.59
102.65
77.41 CDCl3
77.10 CDCl3
76.78 CDCl3
64.78
47.61
35.07
30.57
19.08
13.69
CH3
O
N
N
f1 (ppm)

3m

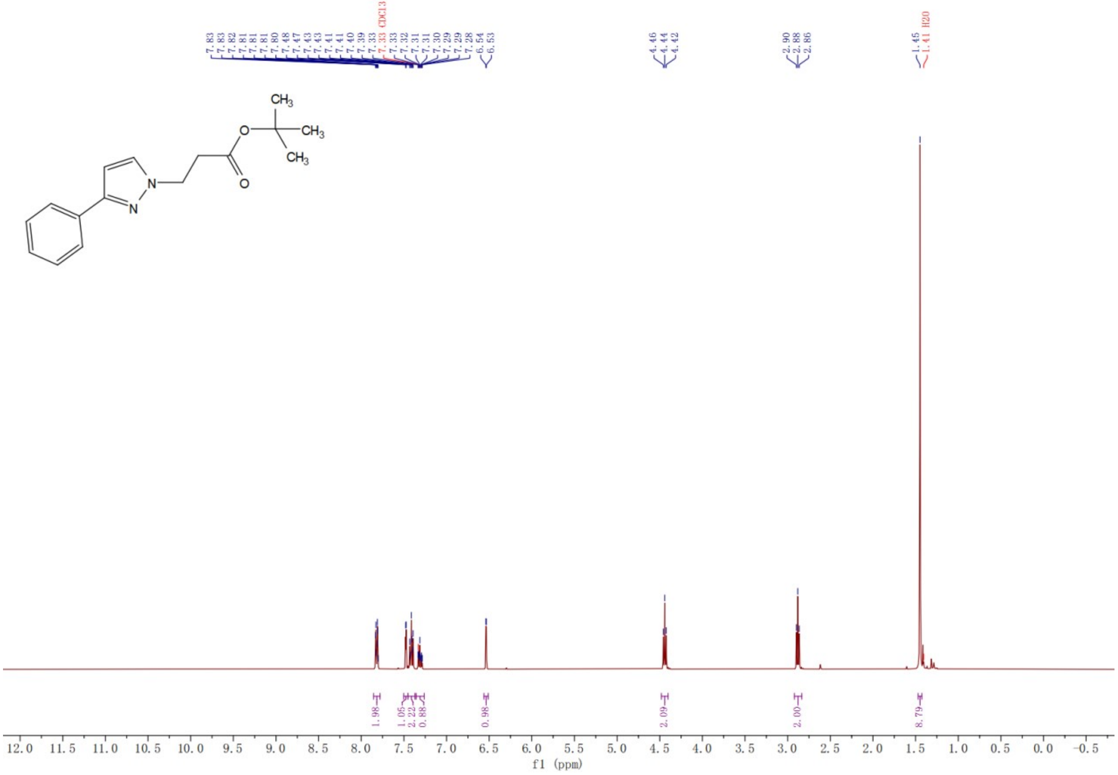
7.83
7.83
7.82
7.81
7.81
7.81
7.80
7.48
7.47
7.43
7.43
7.41
7.41
7.40
7.39
7.33
7.33 CDCl3
7.33
7.32
7.31
7.31
7.30
7.29
7.29
7.28
6.54
6.53
4.46
4.44
4.42
2.90
2.88
2.86
1.45
1.41 H2O
1.98
1.05
2.22
0.88
0.98
2.09
2.00
8.79
f1 (ppm)

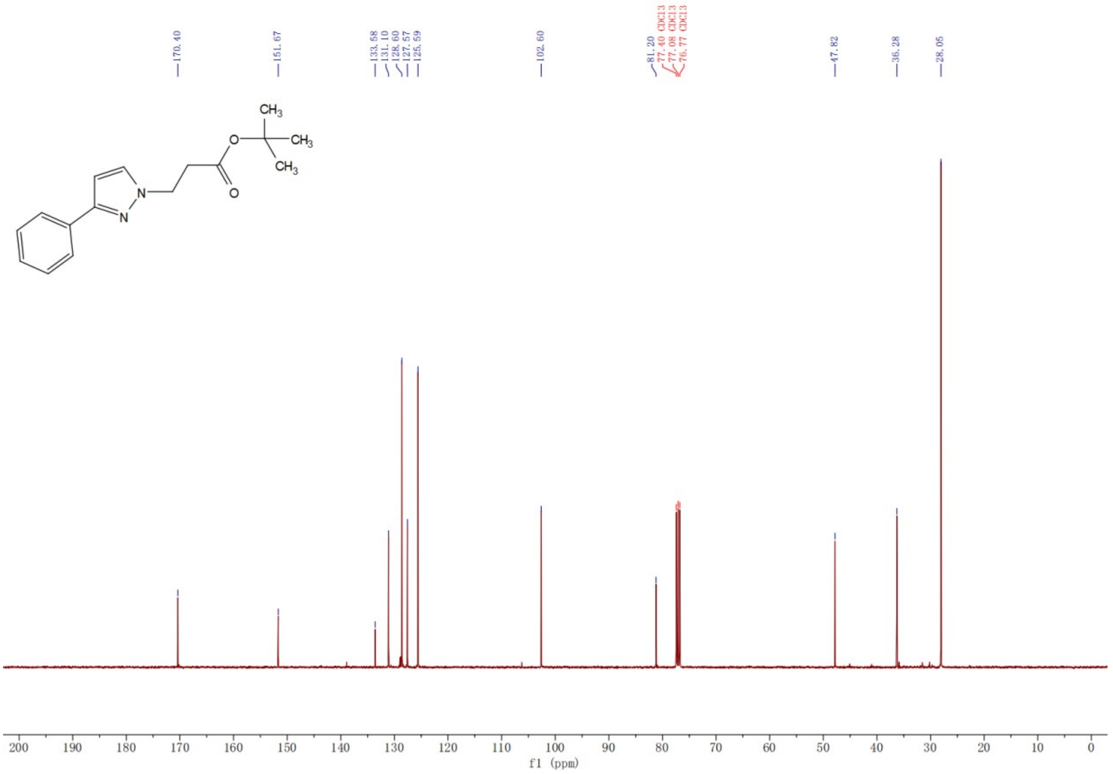
170.40
151.67
133.58
131.10
128.60
127.57
125.59
102.60
81.20
77.40 CDCl3
77.08 CDCl3
76.77 CDCl3
47.82
36.28
28.05
f1 (ppm)

3o

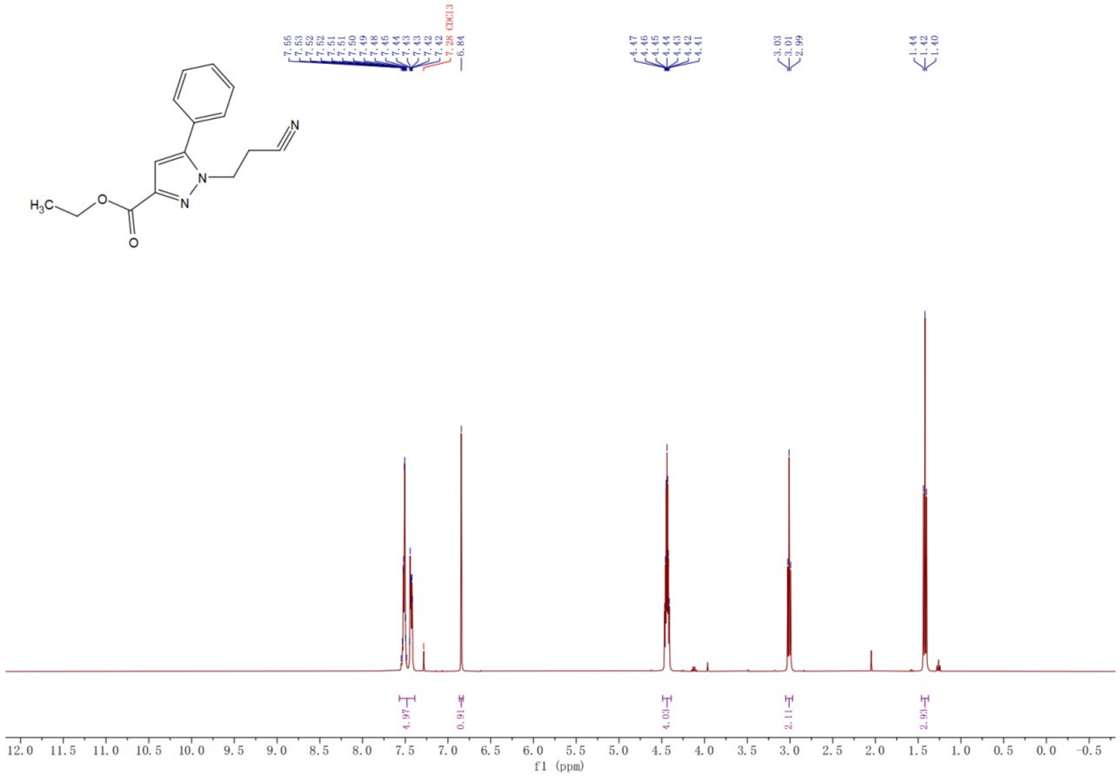

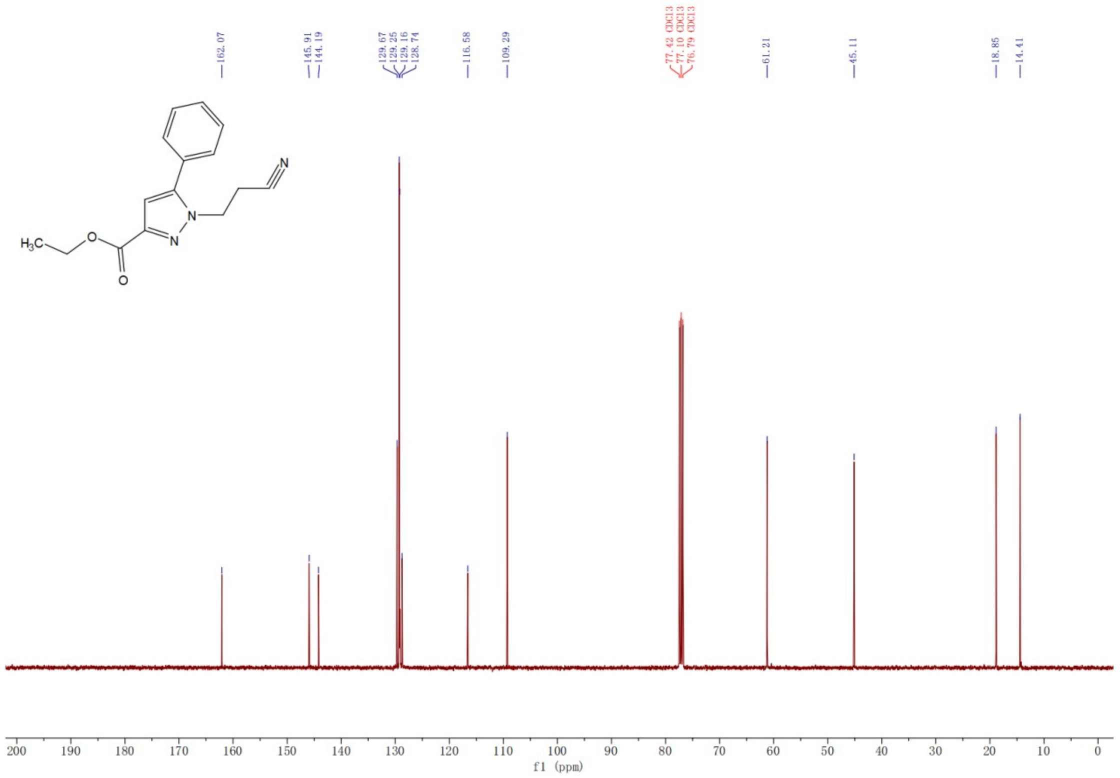

3p

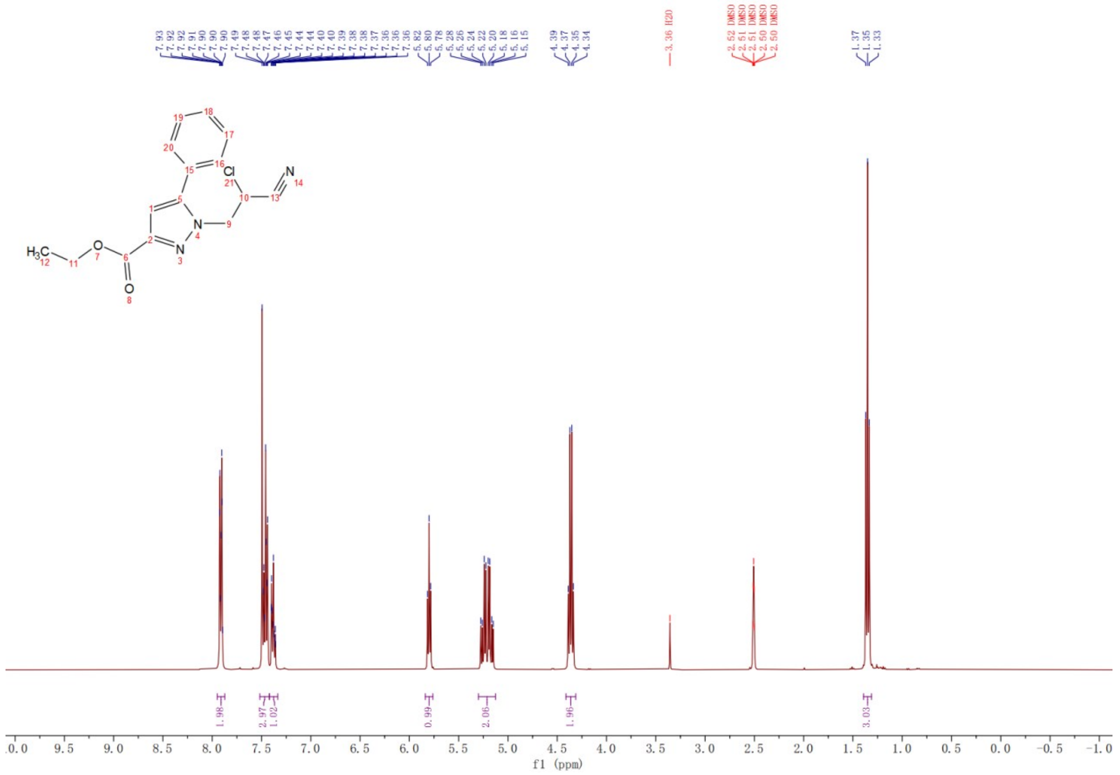

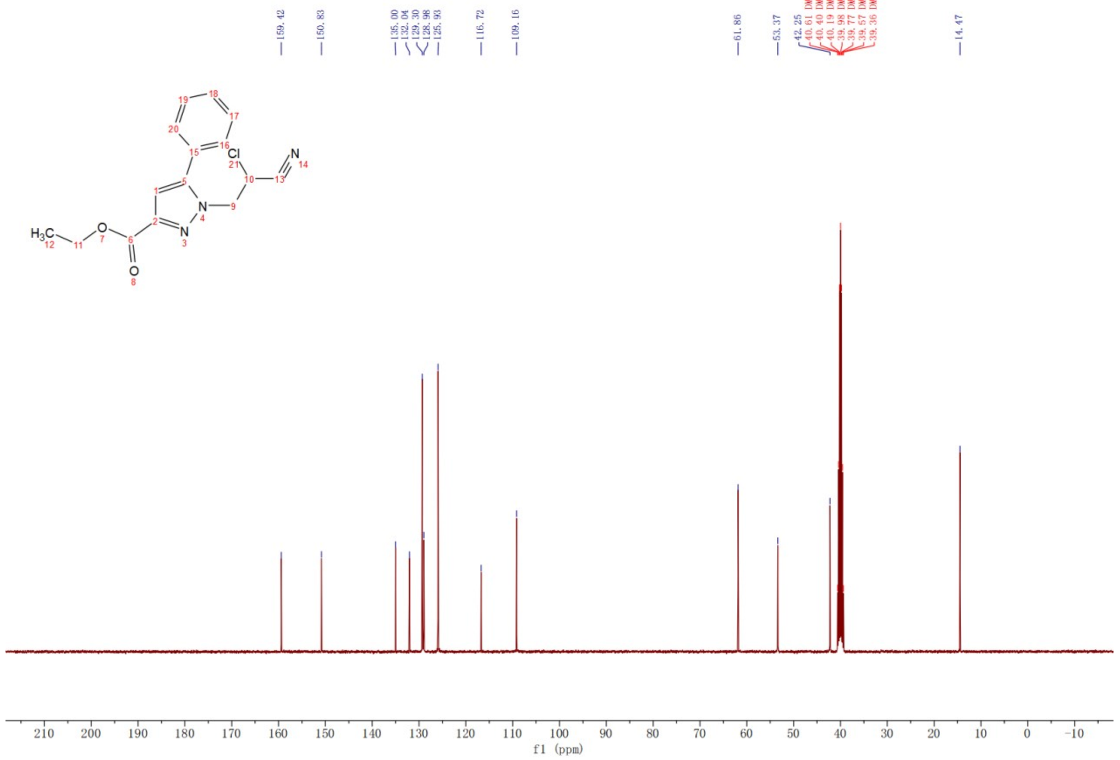

3q

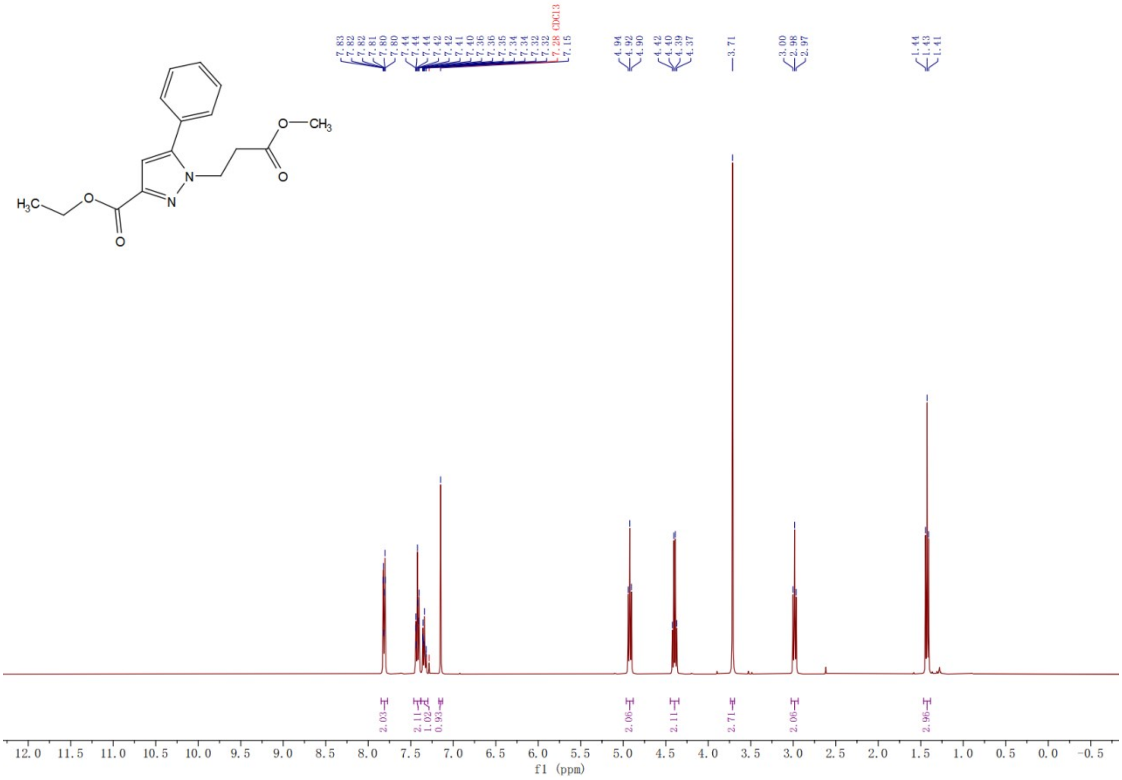
7.83
7.82
7.81
7.80
7.80
7.44
7.44
7.44
7.42
7.42
7.41
7.40
7.36
7.36
7.35
7.34
7.34
7.32
7.32
7.28 CDCl3
7.15
4.94
4.92
4.90
4.42
4.40
4.39
4.37
3.71
3.00
2.98
2.97
1.44
1.43
1.41
2.03
2.11
1.02
0.93
2.06
2.11
2.71
2.06
2.96
f1 (ppm)

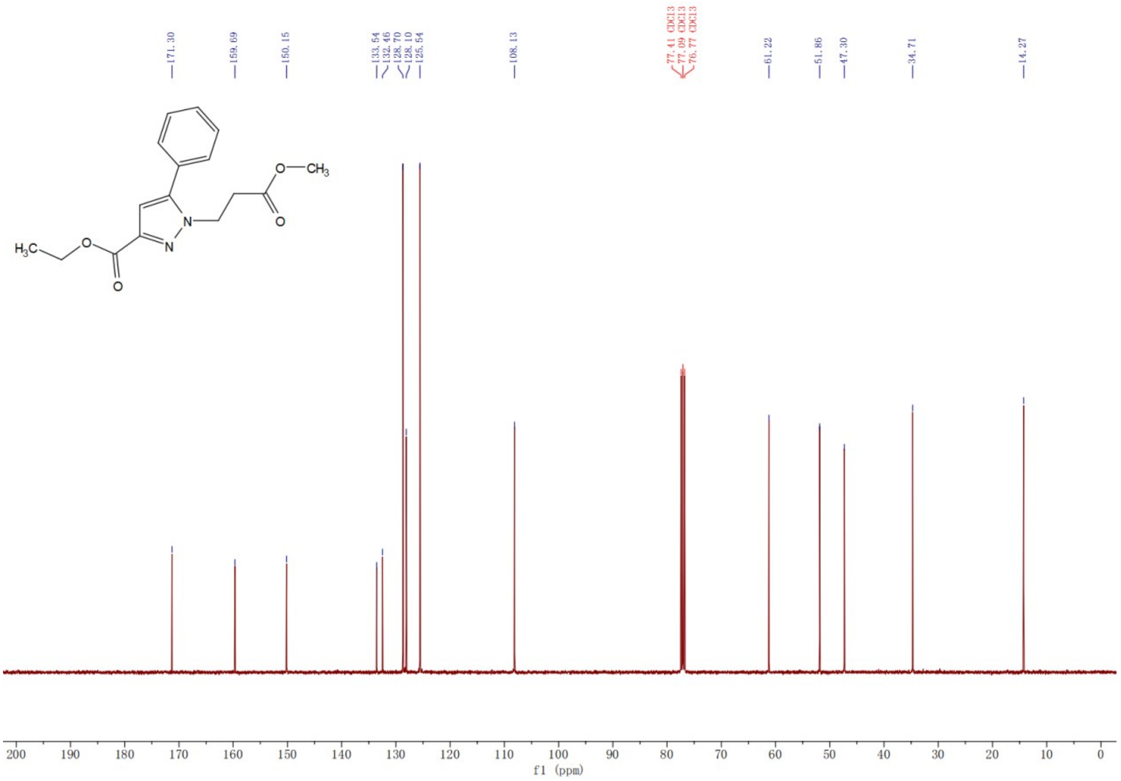
171.30
159.69
150.15
133.54
132.46
128.70
128.10
125.54
108.13
77.41 CDCl3
77.09 CDCl3
76.77 CDCl3
61.22
51.86
47.30
34.71
14.27
f1 (ppm)

3r

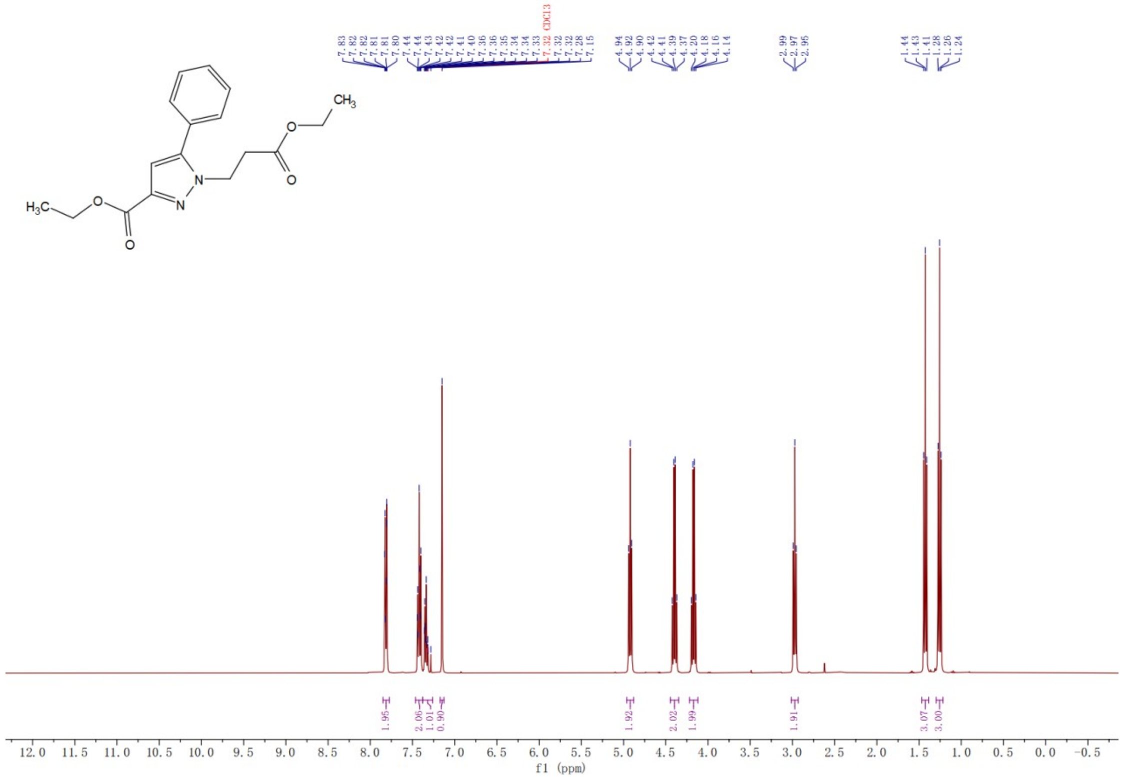

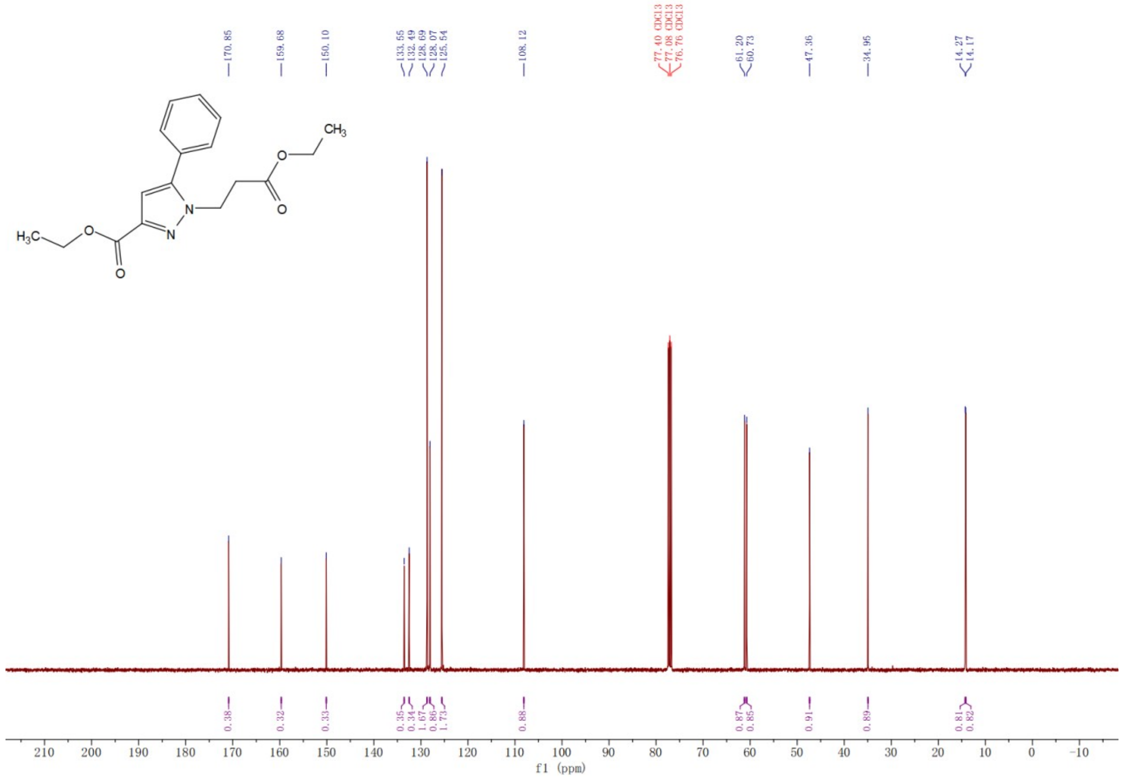

3s

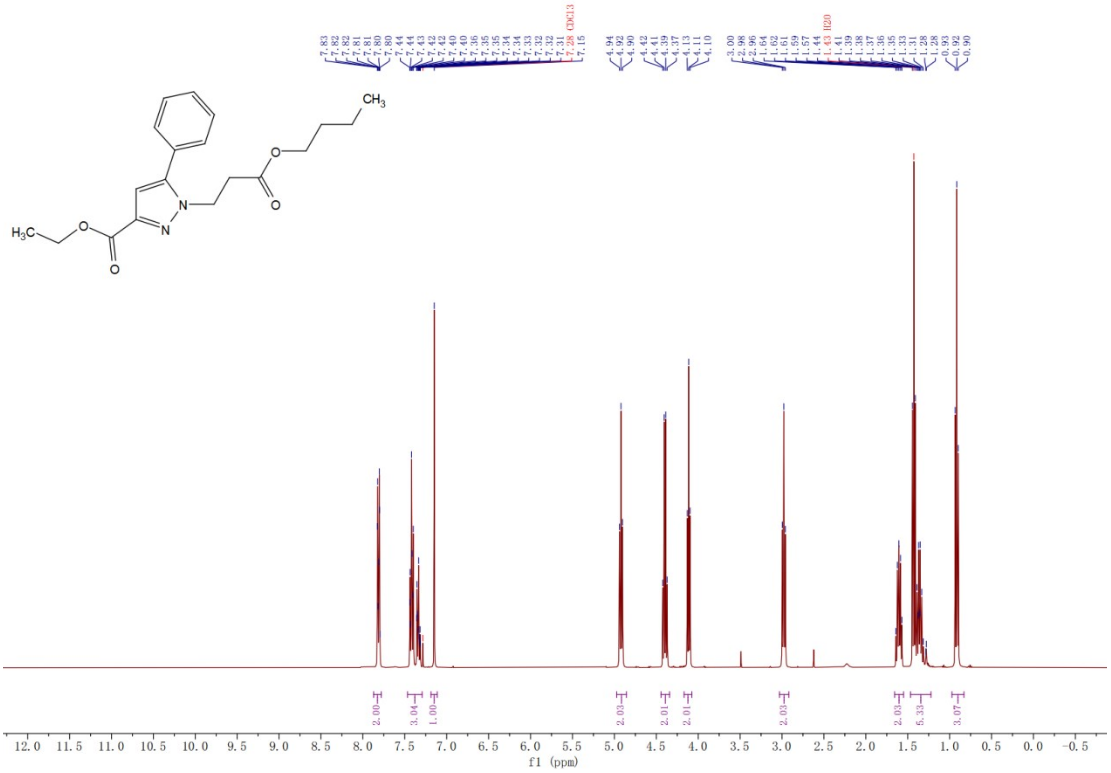

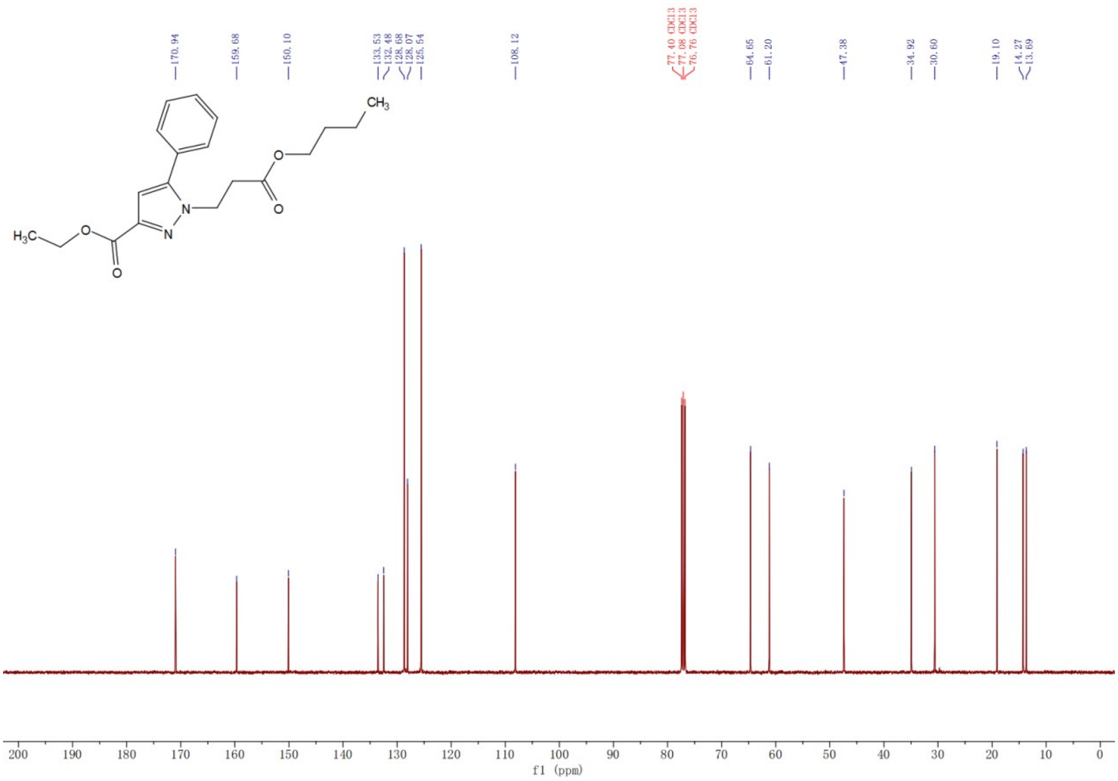

3t

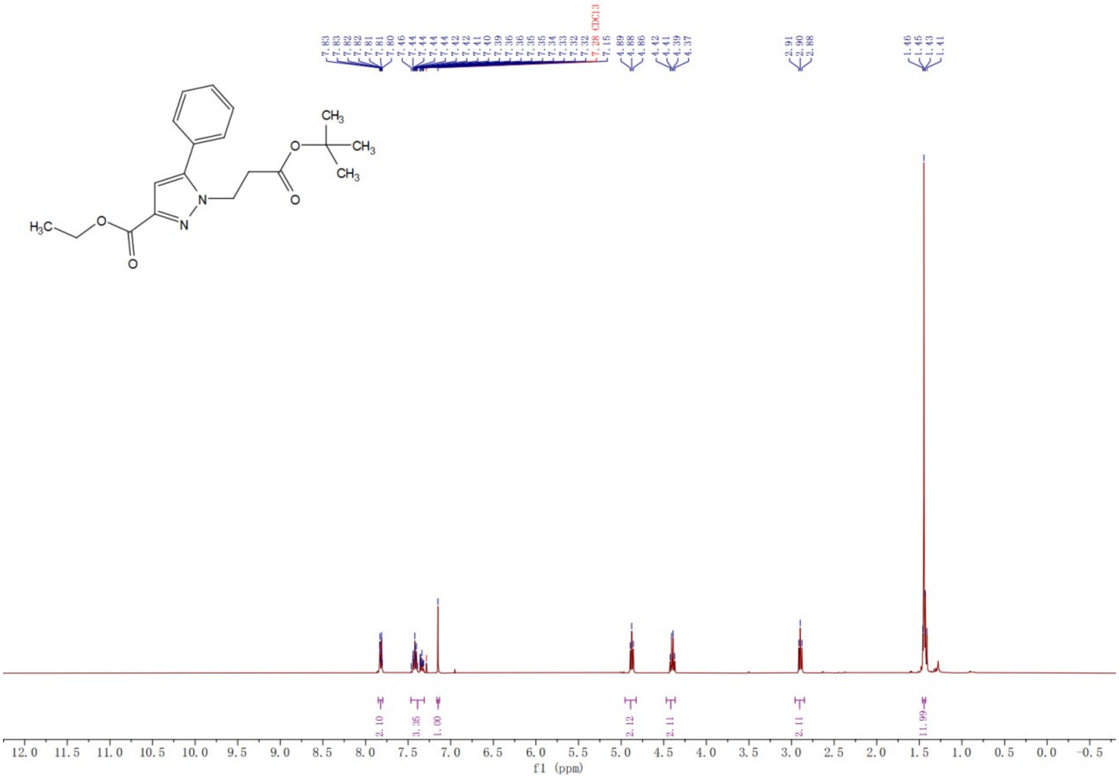

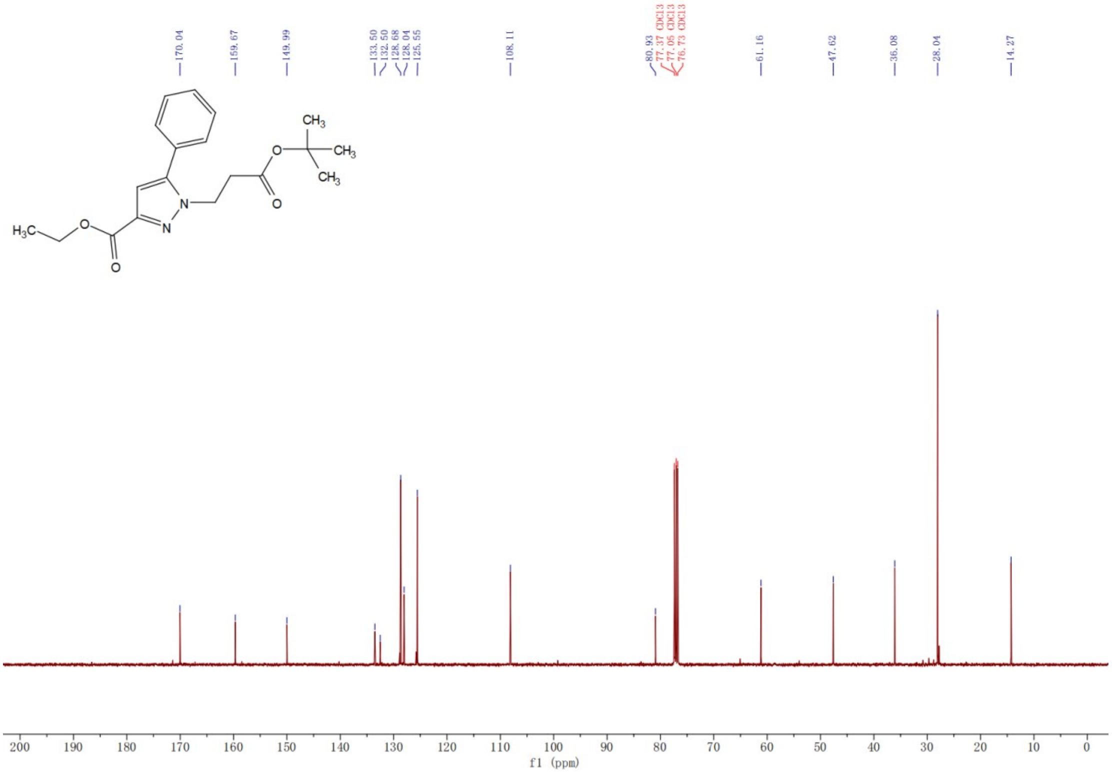

3u

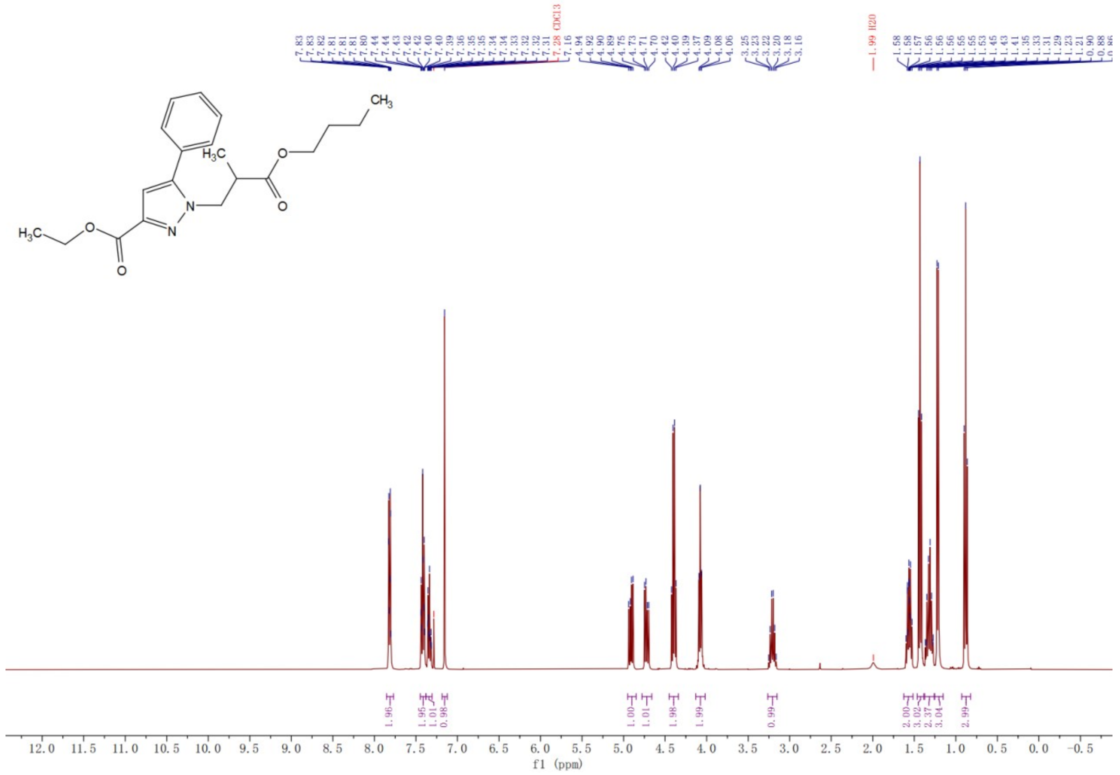
7.83
7.83
7.82
7.81
7.81
7.80
7.44
7.44
7.43
7.42
7.42
7.40
7.40
7.39
7.36
7.35
7.35
7.34
7.34
7.33
7.32
7.32
7.31
7.28 CDCl3
7.16
4.94
4.92
4.90
4.89
4.75
4.73
4.71
4.70
4.42
4.40
4.39
4.37
4.09
4.08
4.06
3.25
3.23
3.22
3.20
3.18
3.16
1.99 H2O
1.58
1.58
1.57
1.56
1.56
1.56
1.55
1.55
1.53
1.45
1.43
1.41
1.35
1.33
1.31
1.29
1.23
1.21
0.90
0.88
CH3
H3C
O
O
N
N
H3C
O
O
1.96
1.95
1.01
0.98
1.00
1.01
1.98
1.99
0.99
2.00
3.02
2.37
3.04
2.99
12.0
11.5
11.0
10.5
10.0
9.5
9.0
8.5
8.0
7.5
7.0
6.5
6.0
5.5
5.0
4.5
4.0
3.5
3.0
2.5
2.0
1.5
1.0
0.5
0.0
-0.5
f1 (ppm)

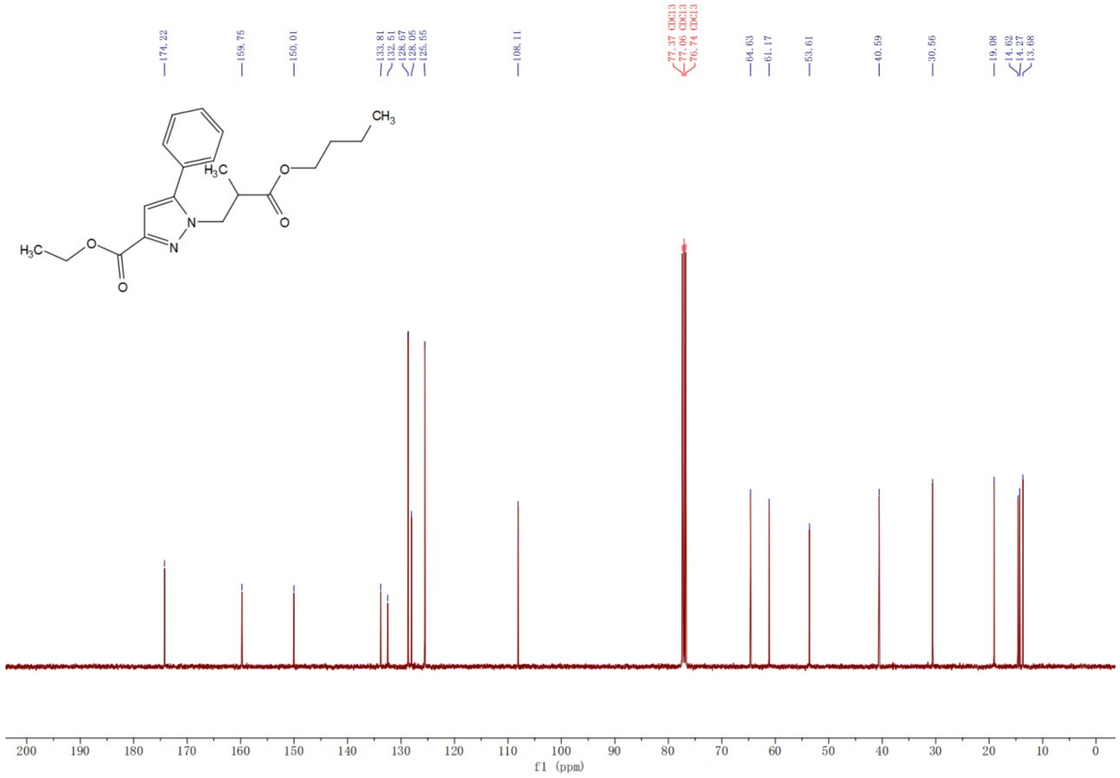
174.22
159.75
150.01
133.81
132.51
128.67
128.05
125.55
108.11
77.37 CDCl3
77.06 CDCl3
76.74 CDCl3
64.63
61.17
53.61
40.59
30.56
19.08
14.62
14.27
13.68
CH3
H3C
O
O
N
N
H3C
O
O
200
190
180
170
160
150
140
130
120
110
100
90
80
70
60
50
40
30
20
10
0
f1 (ppm)

3w

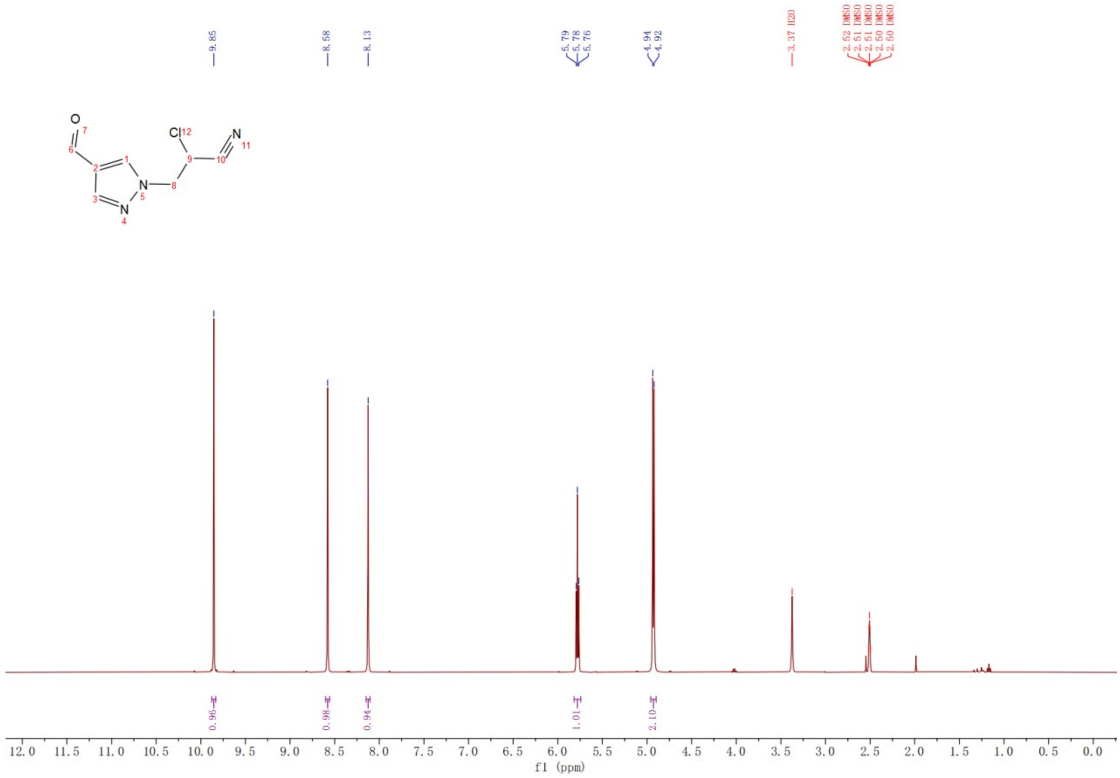

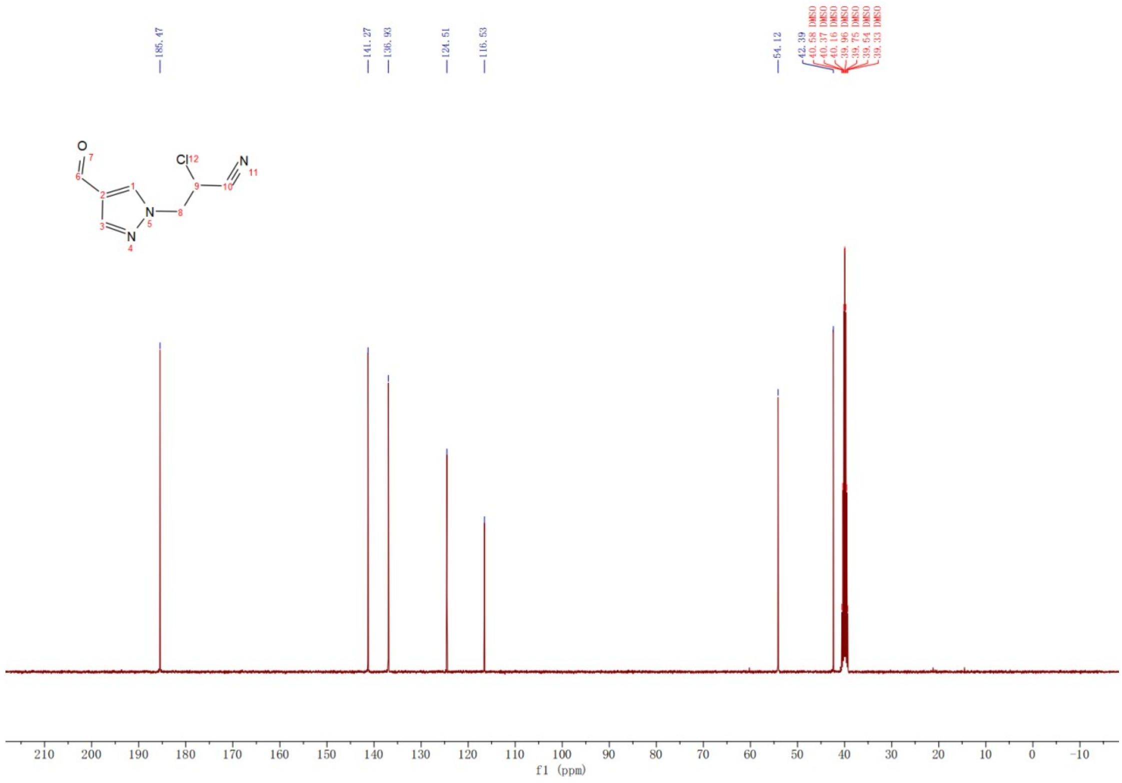

3x

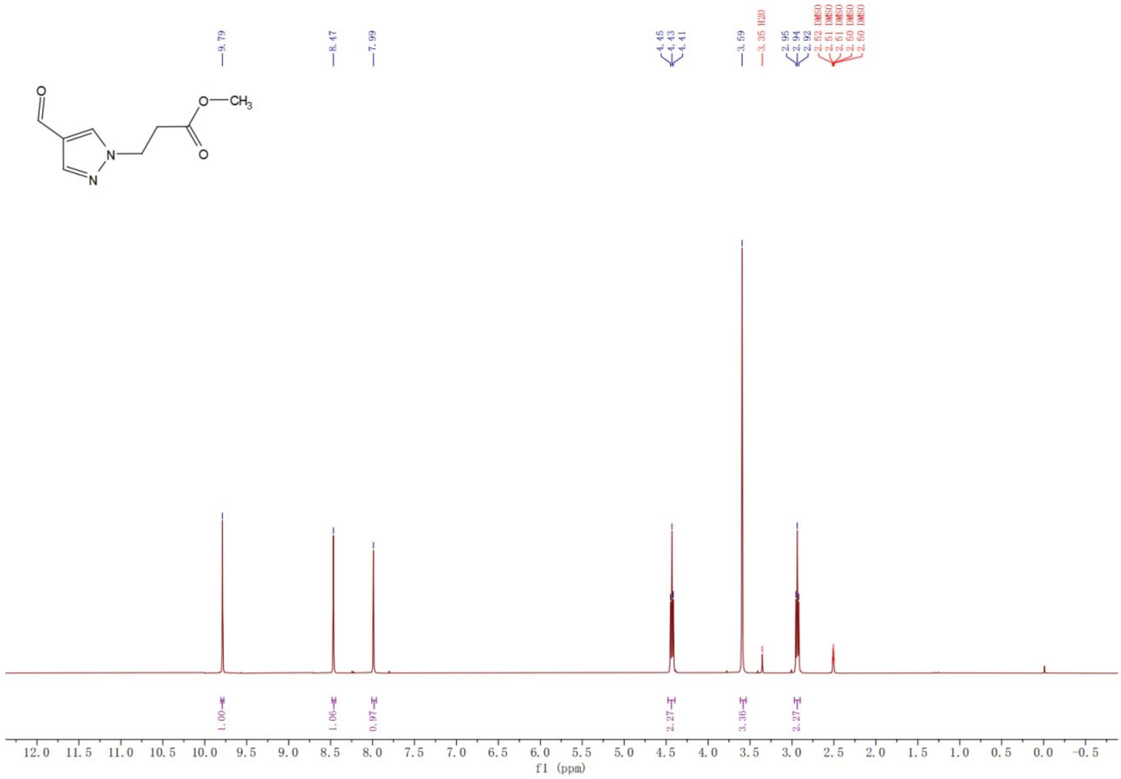

9.79
8.47
7.99
4.45
4.43
4.41
3.59
3.35 H2O
2.95
2.94
2.92
2.52 DMSO
2.51 DMSO
2.51 DMSO
2.50 DMSO
2.50 DMSO
O
O
CH3
N
N
O
1.00
1.06
0.97
2.27
3.36
2.27
f1 (ppm)

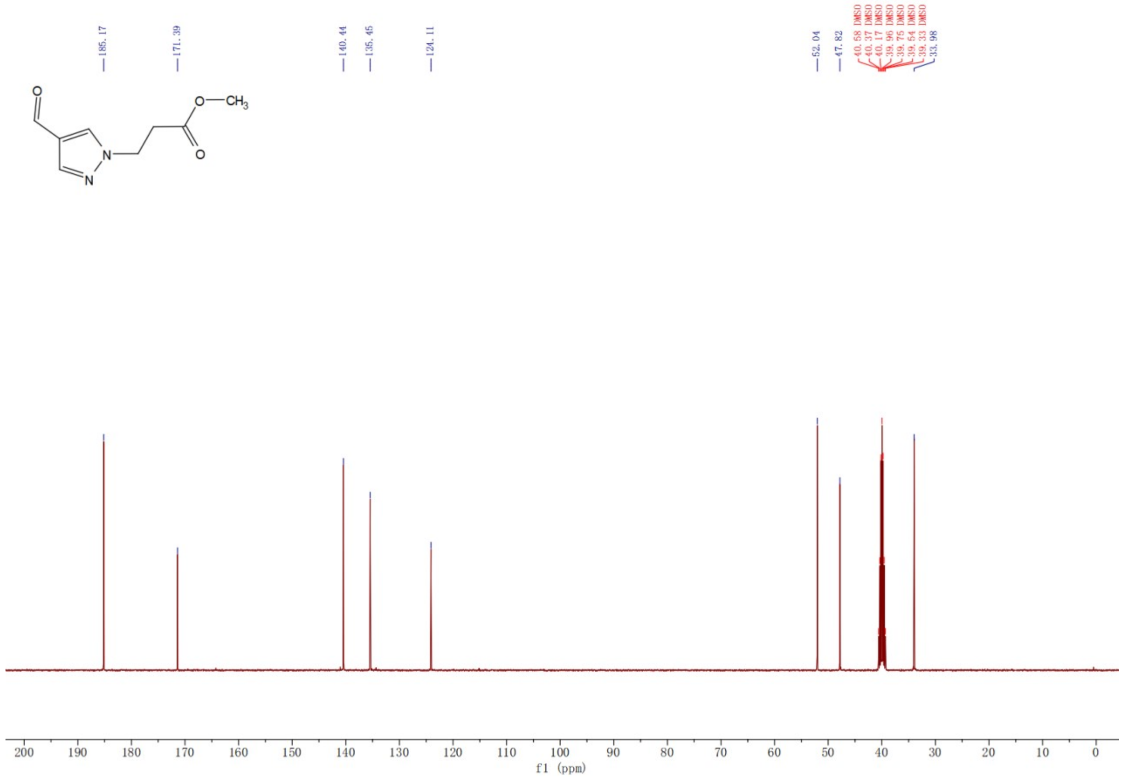

185.17
171.39
140.44
135.45
124.11
52.04
47.82
40.58 DMSO
40.37 DMSO
40.17 DMSO
39.96 DMSO
39.75 DMSO
39.54 DMSO
39.33 DMSO
33.98
O
O
CH3
N
N
O
f1 (ppm)

3y

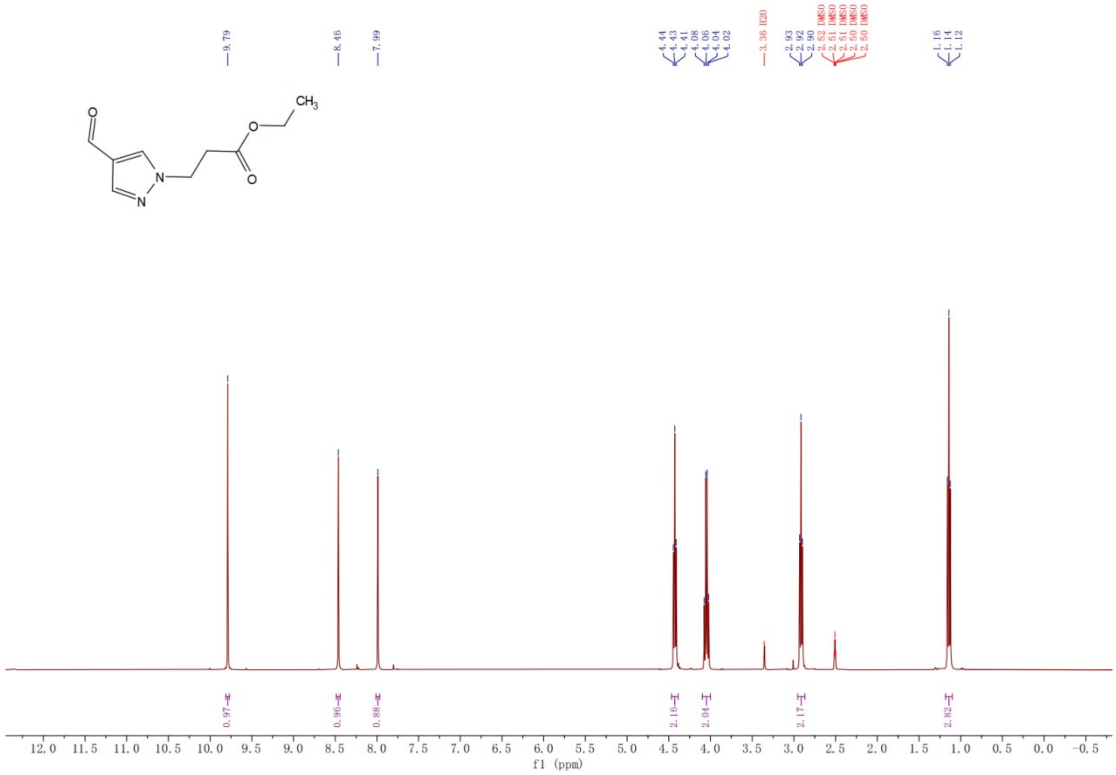

9.79
8.46
7.99
4.44
4.43
4.41
4.08
4.06
4.04
4.02
3.36 H2O
2.93
2.92
2.90
2.52 DMSO
2.51 DMSO
2.51 DMSO
2.50 DMSO
2.50 DMSO
1.16
1.14
1.12
CH3
O
N
0.97
0.96
0.88
2.16
2.04
2.17
2.82
f1 (ppm)

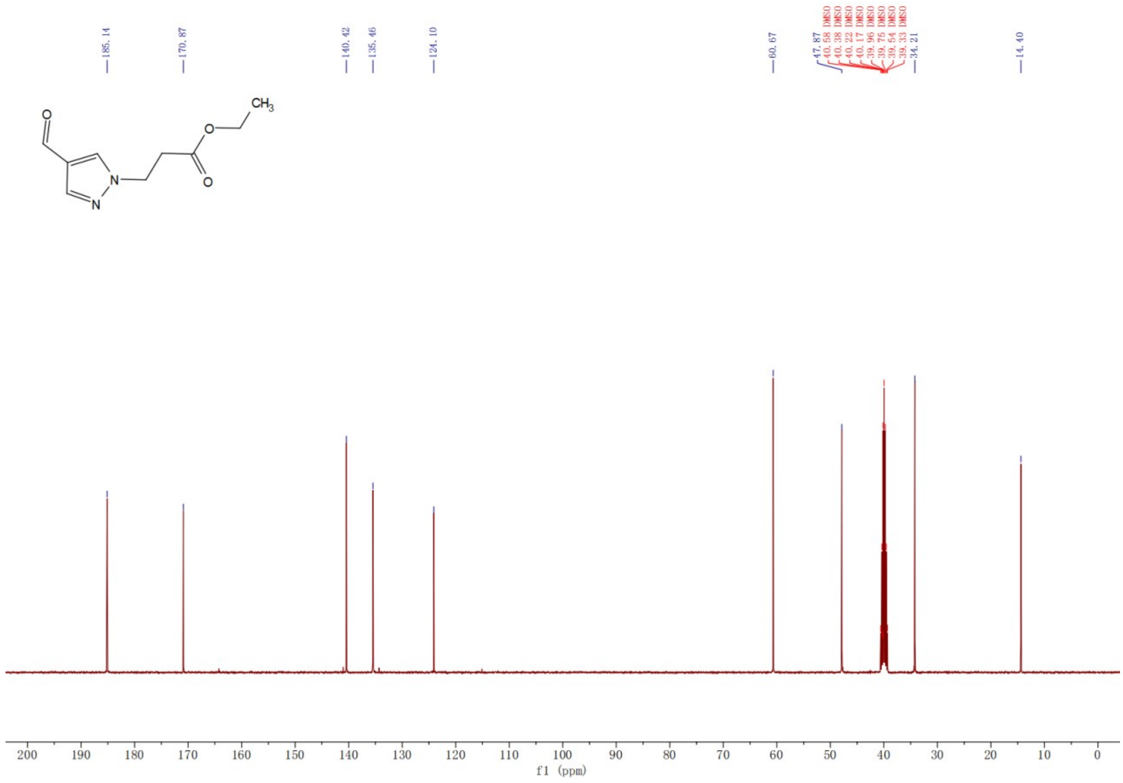

185.14
170.87
140.42
135.46
124.10
60.67
47.87
40.58 DMSO
40.38 DMSO
40.22 DMSO
40.17 DMSO
39.96 DMSO
39.75 DMSO
39.54 DMSO
39.33 DMSO
34.21
14.40
CH3
O
N
f1 (ppm)

3z

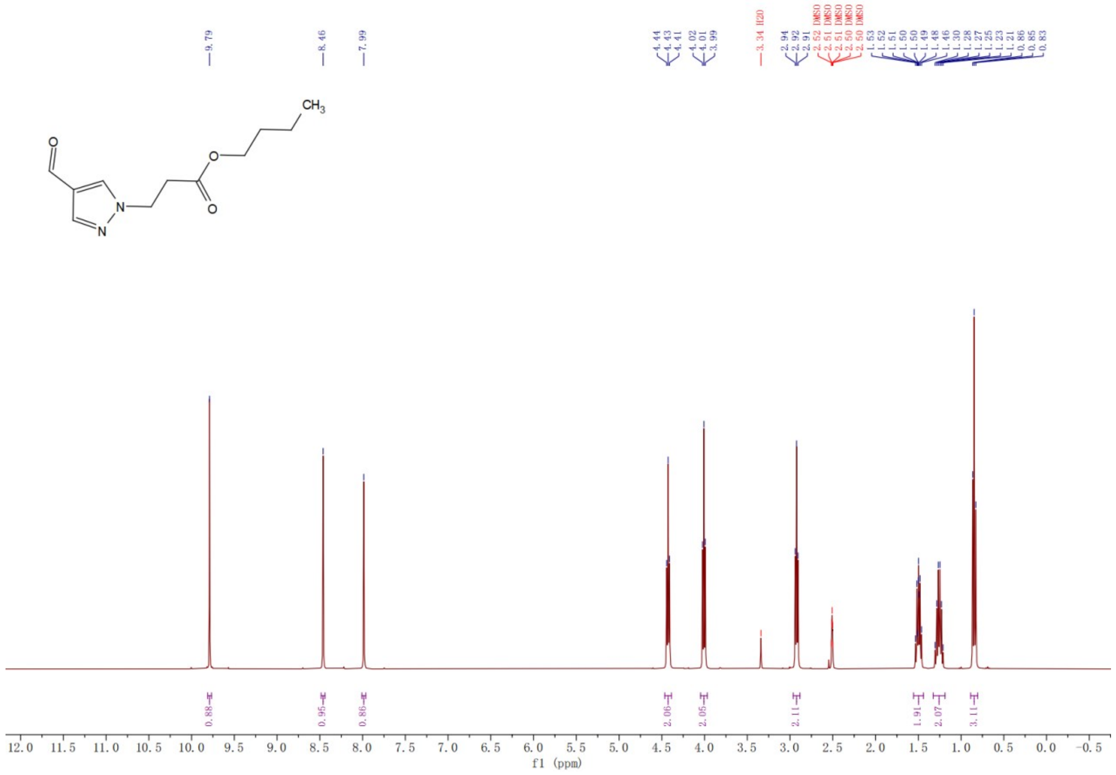

9.79
8.46
7.99
4.44
4.43
4.41
4.02
4.01
3.99
3.34 H2O
2.94
2.92
2.91
2.52 DMSO
2.51 DMSO
2.51 DMSO
2.50 DMSO
2.50 DMSO
1.53
1.52
1.51
1.50
1.49
1.48
1.46
1.30
1.28
1.27
1.25
1.23
1.21
0.86
0.85
0.83
0.88
0.95
0.86
2.06
2.05
2.11
1.91
2.07
3.11
f1 (ppm)

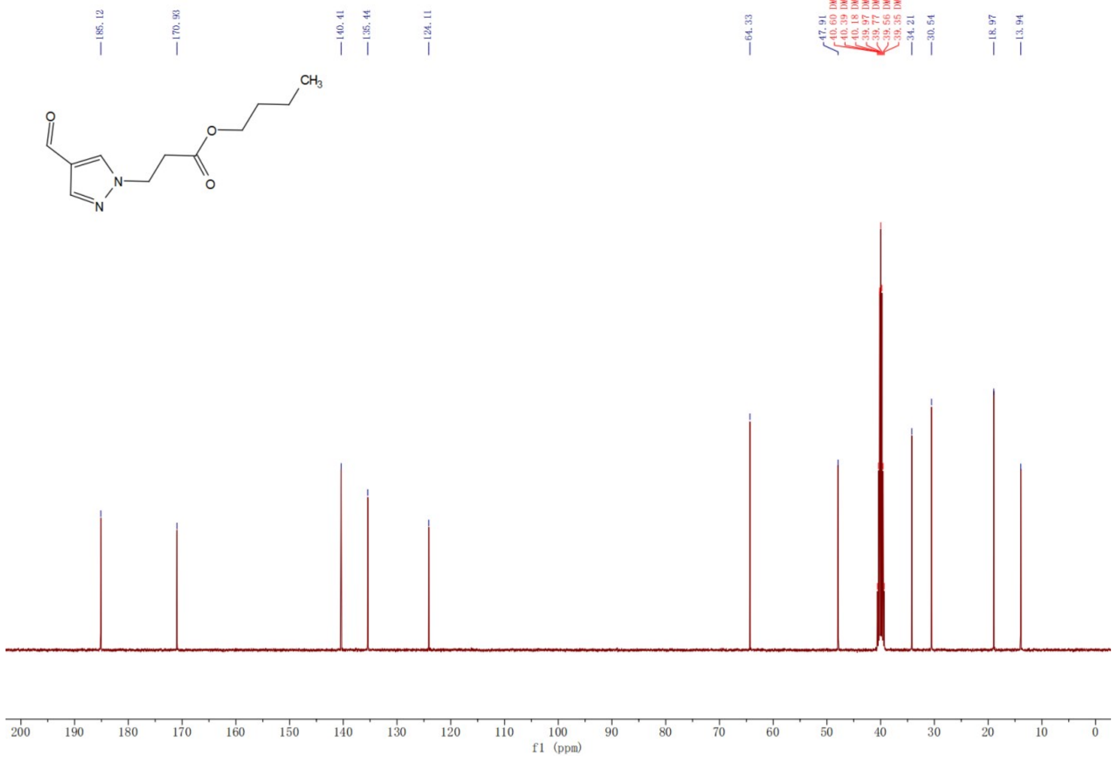

185.12
170.93
140.41
135.44
124.11
64.33
47.91
40.60 DMSO
40.39 DMSO
40.18 DMSO
39.97 DMSO
39.77 DMSO
39.56 DMSO
39.35 DMSO
34.21
30.54
18.97
13.94
f1 (ppm)

3aa

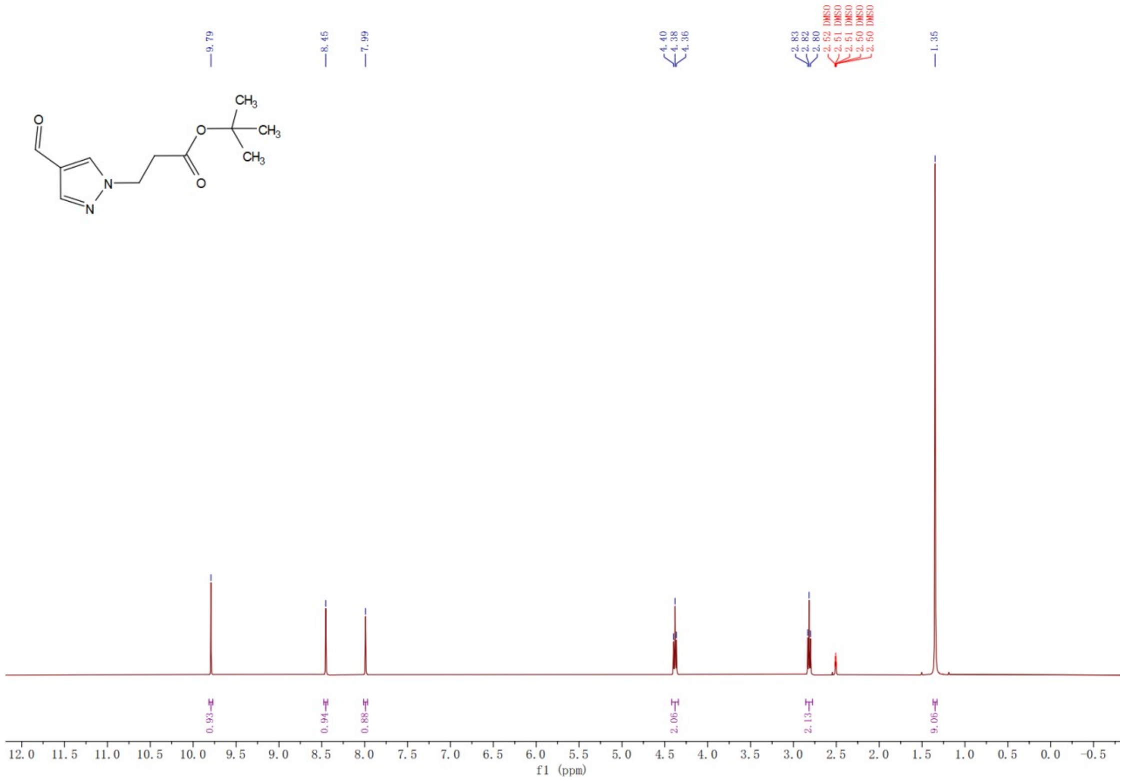
9.79
8.45
7.99
4.40
4.38
4.36
2.83
2.82
2.80
2.52 DMSO
2.51 DMSO
2.51 DMSO
2.50 DMSO
2.50 DMSO
1.35
0.93
0.94
0.88
2.06
2.13
9.06
f1 (ppm)

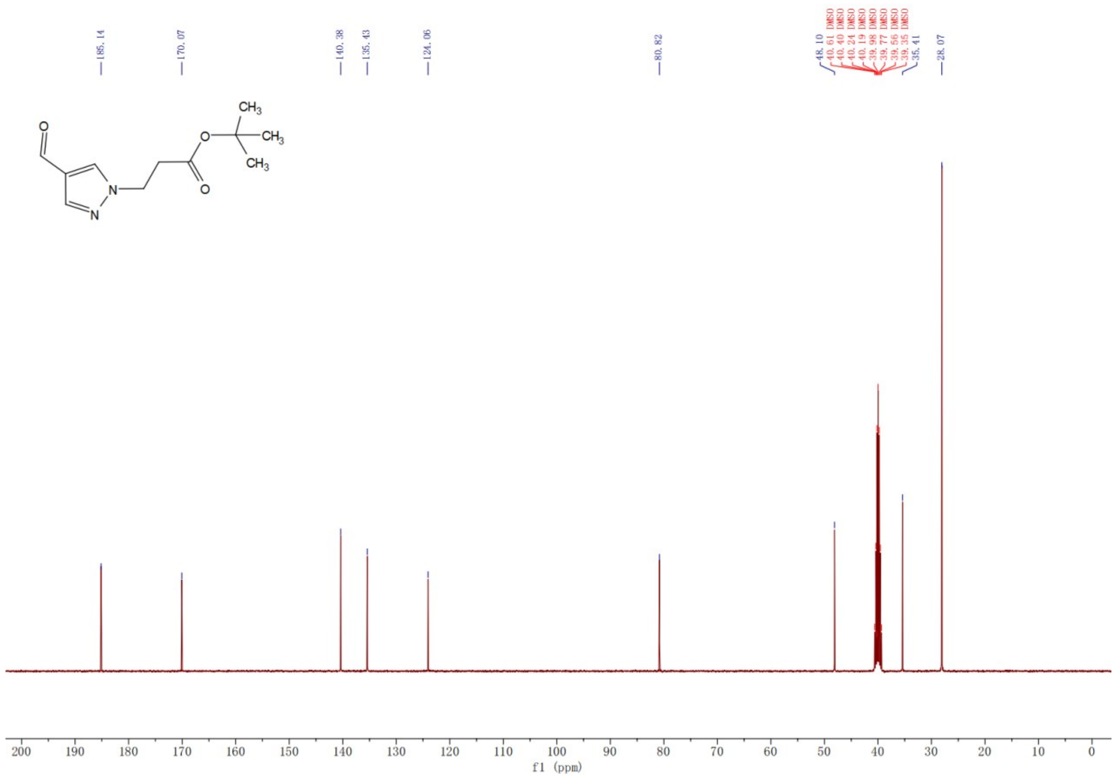
185.14
170.07
140.38
135.43
124.06
80.82
48.10
40.61 DMSO
40.40 DMSO
40.24 DMSO
40.19 DMSO
39.98 DMSO
39.77 DMSO
39.56 DMSO
39.35 DMSO
35.41
28.07
f1 (ppm)

3ab

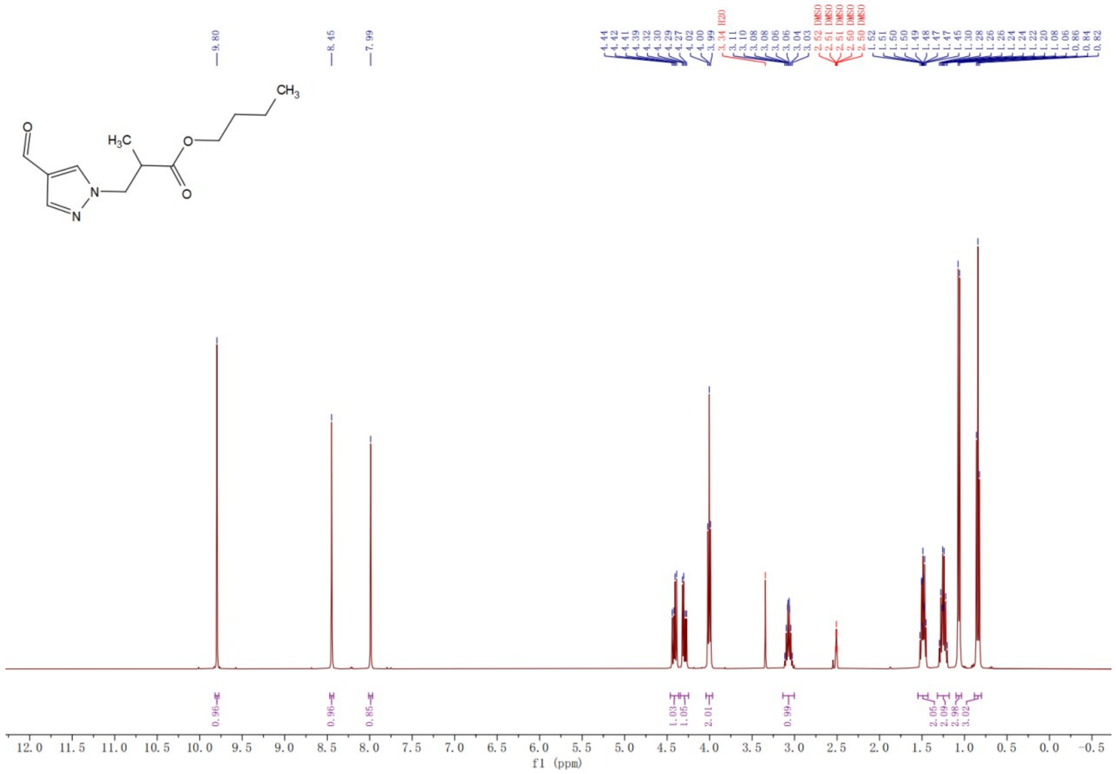
9.80
8.45
7.99
CH3
H3C
O
N
f1 (ppm)

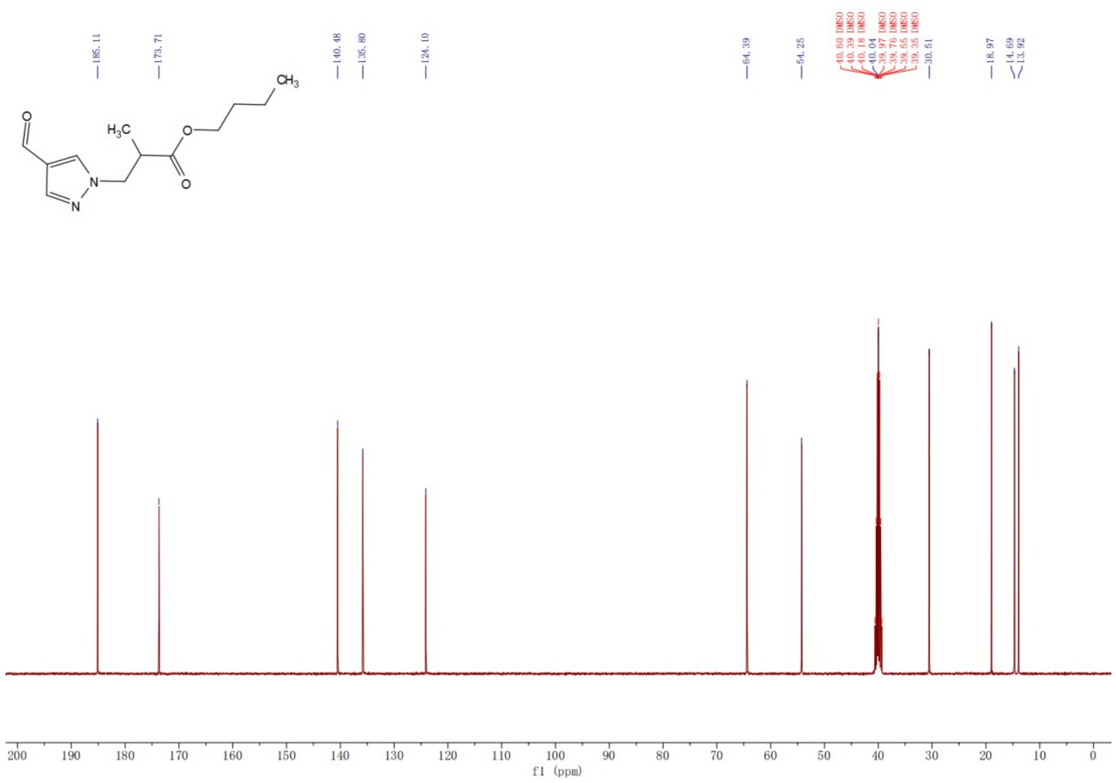
185.11
173.71
140.48
135.80
124.10
64.39
54.25
30.51
18.97
14.69
13.92
f1 (ppm)
